# Assembly of the 373k gene space of the polyploid sugarcane genome reveals reservoirs of functional diversity in the world's leading biomass crop

**DOI:** 10.1093/gigascience/giz129

**Published:** 2019-11-29

**Authors:** Glaucia Mendes Souza, Marie-Anne Van Sluys, Carolina Gimiliani Lembke, Hayan Lee, Gabriel Rodrigues Alves Margarido, Carlos Takeshi Hotta, Jonas Weissmann Gaiarsa, Augusto Lima Diniz, Mauro de Medeiros Oliveira, Sávio de Siqueira Ferreira, Milton Yutaka Nishiyama, Felipe ten-Caten, Geovani Tolfo Ragagnin, Pablo de Morais Andrade, Robson Francisco de Souza, Gianlucca Gonçalves Nicastro, Ravi Pandya, Changsoo Kim, Hui Guo, Alan Mitchell Durham, Monalisa Sampaio Carneiro, Jisen Zhang, Xingtan Zhang, Qing Zhang, Ray Ming, Michael C Schatz, Bob Davidson, Andrew H Paterson, David Heckerman

**Affiliations:** 1 Departamento de Bioquímica, Instituto de Química, Universidade de São Paulo, Av. Prof. Lineu Prestes, 748, São Paulo, SP 05508-000, Brazil; 2 Departamento de Botânica, Instituto de Biociências, Universidade de São Paulo, Rua do Matão, 277, São Paulo, SP 05508-090, Brazil; 3 Cold Spring Harbor Laboratory, One Bungtown Road, Koch Building #1119, Cold Spring Harbor, NY11724, United States of America; 4 Department of Energy Joint Genome Institute, 2800 Mitchell Drive, Walnut Creek, CACA94598, United States of America; 5 Departamento de Genética, Escola Superior de Agricultura Luiz de Queiroz, Universidade de São Paulo, Avenida Pádua Dias, 11, Piracicaba, SP 13418-900, Brazil; 6 Laboratório Especial de Toxinologia Aplicada, Instituto Butantan, Av. Vital Brasil, 1500, São Paulo, SP05503-900, Brazil; 7 Departamento de Microbiologia, Instituto de Ciências Biomédicas, Universidade de São Paulo, Av.Professor Lineu Prestes, 1734, São Paulo, SP 05508-900, Brazil; 8 Microsoft Research, One Microsoft Way, Redmond, WA 98052, United States of America; 9 Plant Genome Mapping Laboratory, University of Georgia, 120 Green Street, Athens, GA 30602-7223,United States of America; 10 Department of Crop Science, Chungnam National University, 99 Daehak Ro Yuseong Gu, Deajeon,34134, South Korea; 11 Departamento de Ciências da Computação, Instituto de Matemática e Estatística, Universidade de São Paulo, Rua do Matão, 1010, São Paulo, SP 05508-090, Brazil; 12 Departamento de Biotecnologia e Produção Vegetal e Animal, Centro de Ciências Agrárias, Universidade Federal de São Carlos, Rodovia Washington Luis km 235, Araras, SP 13.565-905, Brazil; 13 FAFU and UIUC-SIB Joint Center for Genomics and Biotechnology, Fujian Agriculture and Forestry University, Shangxiadian Road, Fuzhou 350002, Fujian, China; 14 Department of Plant Biology, University of Illinois at Urbana-Champaign, 201 W. Gregory Dr. Urbana, Urbana, Illinois 61801, United States of America; 15 Departments of Computer Science and Biology, Johns Hopkins University, 3400 North Charles Street,Baltimore, MD 21218-2608, United States of America

**Keywords:** allele, bioenergy, biomass, genome, polyploid

## Abstract

**Background:**

Sugarcane cultivars are polyploid interspecific hybrids of giant genomes, typically with 10–13 sets of chromosomes from 2 *Saccharum* species. The ploidy, hybridity, and size of the genome, estimated to have >10 Gb, pose a challenge for sequencing.

**Results:**

Here we present a gene space assembly of SP80-3280, including 373,869 putative genes and their potential regulatory regions. The alignment of single-copy genes in diploid grasses to the putative genes indicates that we could resolve 2–6 (up to 15) putative homo(eo)logs that are 99.1% identical within their coding sequences. Dissimilarities increase in their regulatory regions, and gene promoter analysis shows differences in regulatory elements within gene families that are expressed in a species-specific manner. We exemplify these differences for sucrose synthase (SuSy) and phenylalanine ammonia-lyase (PAL), 2 gene families central to carbon partitioning. SP80-3280 has particular regulatory elements involved in sucrose synthesis not found in the ancestor *Saccharum spontaneum*. PAL regulatory elements are found in co-expressed genes related to fiber synthesis within gene networks defined during plant growth and maturation. Comparison with sorghum reveals predominantly bi-allelic variations in sugarcane, consistent with the formation of 2 “subgenomes” after their divergence ∼3.8–4.6 million years ago and reveals single-nucleotide variants that may underlie their differences.

**Conclusions:**

This assembly represents a large step towards a whole-genome assembly of a commercial sugarcane cultivar. It includes a rich diversity of genes and homo(eo)logous resolution for a representative fraction of the gene space, relevant to improve biomass and food production.

## Background

Sugarcane is the world's most cultivated crop in tonnage (more than rice, maize, and wheat) [1] and is considered the most sustainable of energy crops [2], with high potential to mitigate climate change without affecting food security [3]. Already produced in >100 countries, high productivity of sugar, bioethanol, and bioelectricity [4] make it a highly expandable green alternative to petroleum [5–7]. The International Energy Agency projects a 150 EJ (17% of energy demand) contribution of bioenergy by 2060, delivering 18% of the emission reductions needed to achieve the 2DS (2°C scenario). Sugarcane bioenergy production by 2045 could displace up to 13.7% of crude oil consumption and 5.6% of the world's CO_2_ emissions relative to 2014. This can be achieved without using forest preservation areas or land necessary for food production systems. Additionally, the myriad of products that can derive from sugarcane biomass [8] further enhance opportunities for sugarcane in a portfolio of technologies needed to transition to a low-carbon “bioeconomy.”

Opportunities to accelerate breeding progress and enrich knowledge of the fundamental biology of this important plant motivate efforts to produce a high-quality reference genome, a challenge that is unusually complex. Unlike wheat cultivated species known to be either tetraploid (AABB) or hexaploid (AABBDD), the *Saccharum* (sugarcane) genus is considered to be a species complex. A recent study [9] proposed independent polyploidization events within *Saccharum* after divergence from the last ancestor shared with *Sorghum*, superimposed upon an additional whole-genome duplication since the diversification of grasses. As a consequence, the sugarcane genome is redundant and harbors genes in multiple functional copies. Adding further complexity, sugarcane cultivars are polyploid/aneuploid interspecific hybrids, typically with 10–13 sets of their 10 basic chromosomes, 80–85% from *Saccharum officinarum* (2n = 80), which is known for its sweetness, 10–15% from *Saccharum spontaneum* (2n = 40–128) known for its robustness, and ∼5% with recombined chromosomes between those 2 progenitors [10,11]. The ploidy, hybridity, and sheer size of the genome, estimated to have >10 Gb, pose a great challenge for sequencing [12]. Recently released sequences of the modern cultivar R570 yielded a mosaic monoploid reference (382 Mb single tiling path) [13] and an *S. spontaneum* AP85-441 haploid assembly (3.13 Gb) [14].

Worldwide sugarcane yield (∼84 ton/ha) is currently only ∼20% of the theoretical potential (∼381 ton/ha), spurring great interest in conventional or molecular breeding approaches to improve it. However, progress by conventional breeding towards closing the gap between current and potential yield has been slow, with gains on the order of 1.0–1.5% a year [15]. Sugarcane commercial cultivars distribute roughly one-third of their carbon into sucrose and two-thirds into tops and stems, which, due to high lignin content, are burned to fuel boilers, contributing to the favorable energy balance of industrial processes [16]. Because sugarcane can accumulate large amounts of sucrose in its stems, up to ∼650 mM [17], it is important to study sucrose metabolism and the key players in its regulation. Also, of interest is the revealing of regulators of cell wall biosynthesis. Altering these pathways may help shift carbon partitioning from sucrose storage to biomass accumulation, rich in fiber content, mostly composed of secondary cell walls formed by cellulose, hemicellulose, and lignin [18]. The latter compound is a hydrophobic polymer that provides strength and rigidity to the plant but also is responsible for cell wall recalcitrance, which is the natural plant resistance to hydrolytic attacks that hampers cellulosic ethanol production [19].

## Results

### The SP80-3280 assembly reveals a gene space of 373,869 genes

Here, we report a representative gene space assembly of the genome sequence of SP80-3280 (GenBank accession number QPEU01000000), the cultivar used in Brazilian breeding programs with the largest collection of transcriptomic data available [20]. In the assembly of 4.26 Gb, 373,869 putative genes and promoter regions were predicted. For a large fraction of the gene space, an average of 6 sugarcane haplotypes, putatively homo(eo)logs, were identified. This is the first release of an assembly of such a giant hybrid polyploid genome with part of the putatively homo(eo)logs resolved and their potential regulatory regions.

The assembly was constructed using 26 libraries sequenced using Illumina Synthetic Long-Read technology, obtaining 19 Gb, ∼19× haploid genome coverage (∼1.9× genome coverage) with >99% of bases having >99% accuracy (Supplemental Fig. S1), which ensure the sequence quality of genes (to be predicted) and intergenic regions (which include the 5′ and 3′ region of genes). The final assembly includes 450,609 contigs (267,287 unitigs + 183,322 singletons), with mean length of 9,452 bp and NG50 of 41,394 bp (Table 1), adding >3 Gb of sequence not previously reported (Supplemental Table S1) [21]. The gene space described here might be explored through a GBrowse environment [22].

**Table 1:** Genome sequencing: technology and assembly details and gene prediction features

Description	Genomic DNA	BAC clones
**Sequencing and assembly data**		
Sequencing data	26 Illumina synthetic long-read libraries	Single-end Roche 454 of BAC library clones
Total sequence (Gb)	19	6.6
Genome coverage	1.9×	0.66×
Read length minimum/maximum/mean (bp)	1,500/22,904/4,930	8/2,611/368.5
Assembler software	Celera Assembler (Overlap Graph)	PHRAP/CONSED
Total reads used in assembly	3,857,849	17,894,306
Total assembly size	4.26 Gb	49.6 Mb
Number of unitigs/contigs + singletons	450,609	463
Contigs length minimum/maximum/mean (bp)	1,500/468,011/9,452	11,723/235,533/107,129
NG50 (bp)	41,394	109,618
N50 (bp)	13,157	N/A
**Gene prediction features**		
No. genes	373,869	3,550
No. transcripts	374,774	
No. exons	1,035,764	13,132
Mean GC content (%)	43.20	44.99
Mean No. exons per gene	2.8	3.7
Mean exon size (bp)	291	271.8
Median exon size (bp)	171	154
Mean intron size (bp)	352.6	539.2
Median intron size (bp)	132	139
Mean gene size (bp) with UTR	1,437.80	2,429.20
Median gene size (bp) with UTR	806	1,260.50
Mean gene size (bp) without UTR	1,318.80	2,351.30
Median gene size (bp) without UTR	771	1,199.50
Mean gene density (kb per gene)	11.4	14

GC: guanine-cytosine; UTR: untranslated region.

Comparisons to different sets of genes were performed: (i) among 39,441 sorghum transcripts, 39,207 (99.4%) matched the assembly, at least partially; of these, 71.1% matched ≥1 sugarcane contig with ≥90% coverage (Supplemental Fig. S2); (ii) the assembly completely covers 217 (87.5%) of the 248 ultra-conserved CEGMA [23] proteins and partly covers 18 (7.3%), with only 13 (5.2%) not detected (Supplemental Table S2); (iii) among 1,440 genes in the BUSCO [24] Plantae lineage, the assembly completely covers 1,309 (90.9%) and partially covers 53 (3.7%) (Supplemental Table S3). By including tBLASTn of the 78 (5.4%) missing Plantae lineage BUSCO genes, only 8 (0.5%) are absent; (iv) assembled chloroplast (NC_0 05878.2) and mitochondrial (LC107874.1and LC107875.1) genomes were >99% similar (at gene level) to published *Saccharum* genomes [25,26]; and (v) 92.8% of 134,840 SP80-3280 expressed sequence tags (ESTs) match the assembled gene space sequence.

The assembly revealed 373,869 putative genes with 374,774 transcripts (Table 1), far more than the 72,269 unigenes inferred from 6 sugarcane genotypes [27]; 85,151 transcripts of sugarcane genotypes with contrasting lignin contents [28]; and 195,765 transcripts inferred from *de novo* assembly of ORFeomes from *S. officinarum, S. spontaneum*, and SP80-3280 [29].

**Figure 1:**
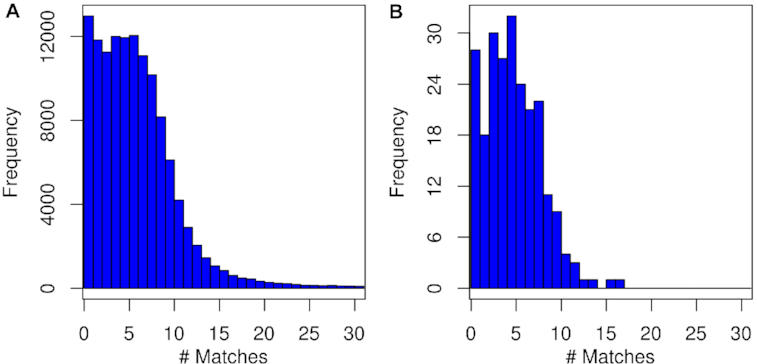
Frequency histogram of expressed sequence tags (ESTs) and CEGMA region alignment on sugarcane genome assembly. For 125,072 aligned ESTs, 106,133 (84.8%) show 2–30 matches on the genome (**A**), while for CEGMA regions, 205 (87.2%) range from 2 to 17 matches on the genome (**B**). SPALN v 2.3.3 [32] was used for alignment.

Among the predicted transcripts, 302,627 (80.7%) aligned to a Uniref50 protein [30], and 195,651 were annotated with 10,362 GO terms [31] (Supplemental Fig. S3). Our previously published SP80-3280 ORFeome was reassembled using the genome as a reference, revealing 269,050 genes and 275,807 transcripts from leaves and immature and intermediate internodes (Supplemental Table S4). Furthermore, a set of 134,840 SP80-3280 ESTs from a Sugarcane EST Project (SUCEST) [20] were mapped to assembled contigs and compared to predicted genes, in order to further estimate the homo(eo)logous abundance of the predicted gene space. A total of 125,072 ESTs (92.8%) have ≥1 match in the assembly, which is in accordance with similar analysis of other plant genomes [33], and only 6.8% of the aligned ESTs (8,499 out of 125,072) do not correspond with predicted genes. This result resembles the BUSCO results, for which only 5.4% of conserved genes could not be identified in the assembly. Although 10.4% of mapped ESTs (12,966) have a unique hit, which may represent sequencing/assembly issues or genes loss, 84.8% of ESTs (106,133) show 2 to 30 matches on the genome, reflecting the presence of the majority of putative homo(eo)logs (Fig.   1A). This result is similar to the search for CEGMA matches against the genome itself using BLASTn. From 235 sequences completely or partially covering CEGMA proteins, 205 have mostly 2–8 (up to 17) matches on the genome (Fig. 1B).

**Figure 2:**
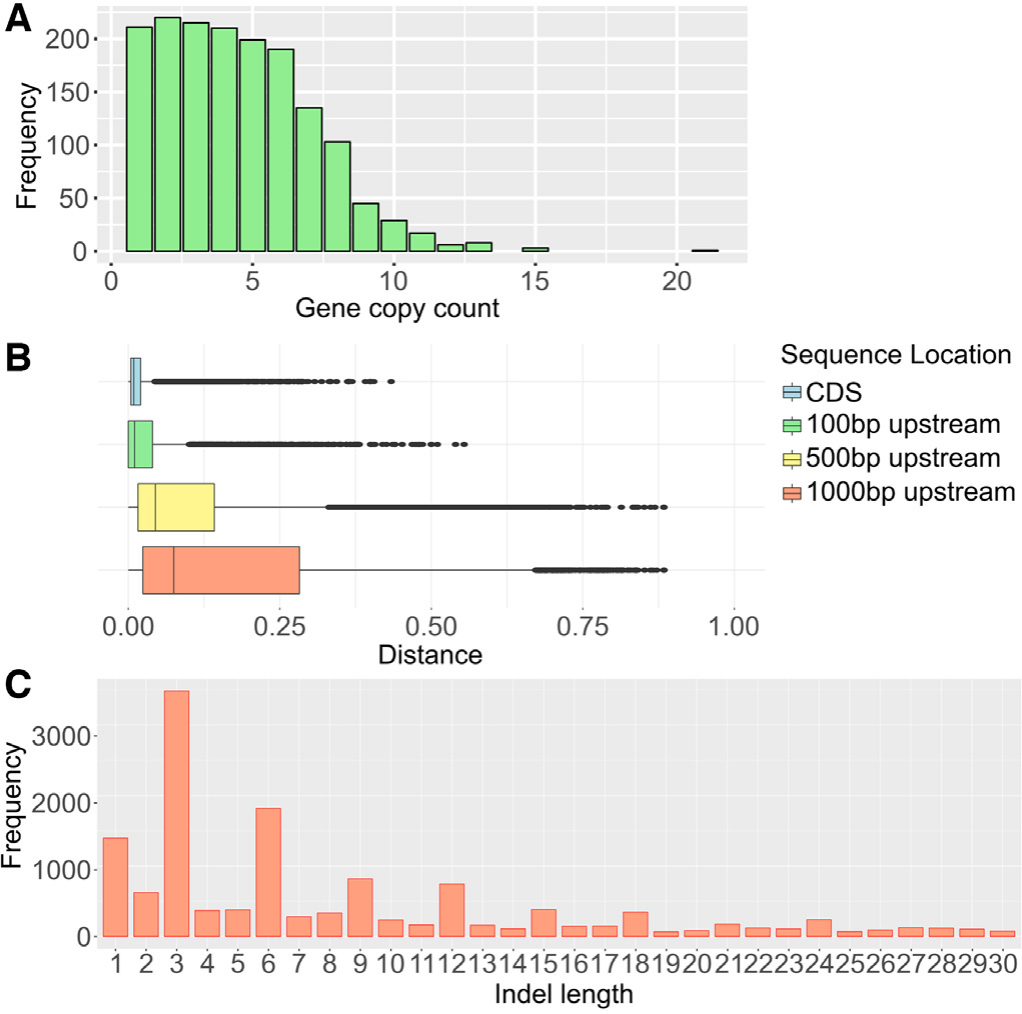
Gene copy number estimation. (**A**) Distribution of copy counts for putative single-copy genes in diploid grasses. From the 2,051 single-copy genes in sorghum, rice, and *Brachypodium*, 1,592 single-copy genes matched to ≥1 sugarcane predicted gene. More than 99.9% of the aligned single-copy genes are present between 1 and 15 times in the sugarcane assembly. (**B**) Copy differentiation between sugarcane coding sequences (CDSs) and upstream regions (sequences of 100bp upstream of the CDS, sequences of 500bp upstream of the CDS and sequences of 1000bp upstream of the CDS), based on pairwise sequence alignment of gene clusters. Genetic dissimilarity increases with increasing distance from the translation start site. (**C**) Indel length distribution in sugarcane putative homo(eo)logs. Frame-preserving indels are more common than frameshifts for this set of genes.

To verify how the assembled gene space reflected the expected content of homo(eo)logous genes, the gene content was compared to those of other grasses. Single-copy genes in diploid grasses (sorghum, rice, and *Brachypodium*) are present in up to 15 copies in sugarcane, mostly with 2–6 copies (total of 1,592 coding sequences [CDSs] in sugarcane) (Fig. 2A). Dissimilarities among putative homo(eo)logs increase from the coding region to the promoter region, with median divergence of 0.90% between CDSs, 1.03% for the 100 nucleotides (nt) upstream, 4.47% for 500 nt, and 7.50% for 1,000 nt (Fig. 2B). Frame-preserving indels are more abundant than frameshifts (Fig. 2C), and short-frameshift indels were relatively less frequent in the sugarcane exons than in sorghum [34].

**Figure 3:**
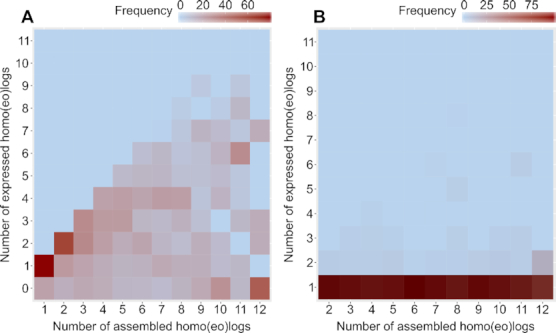
Homo(eo)log expression: The percentage frequency of sugarcane genes plotted against the total number of homo(eo)logs per gene and the number of expressed homo(eo)logs per gene. Genes with complementary DNAs aligned with FPKM > 1 were considered expressed. Plots show sense (**A**) and antisense (**B**) transcripts. Reads from Ion PGM Sequencing were used, and strand orientation is maintained [29].

The SP80-3280 gene series that correspond to single-copy genes in diploid grasses showed expression of sense copies for multiple homo(eo)logs (Fig. 3A), with very few copies transcribed in antisense orientation (Fig. 3B) based on alignment with the SP80-3280 complementary DNA reads [29] from leaves and immature and intermediate internodes. For some genes, not all copies are expressed in SP80-3280 (Fig. 3A and Supplemental Fig. S4A). In addition, the increase in the number of expressed copies is not accompanied by an increase in the level of expression (Supplemental Fig. S4B).

As an example of the complexities in data mining of such an intricate gene space for future reference, we offer an example using 2 well-known genes involved in sucrose and lignin biosynthesis.

### Gene family analysis of SuSy and PAL shows differences in their regulatory regions in SP80-3280 and *S. spontaneum*

**Figure 4:**
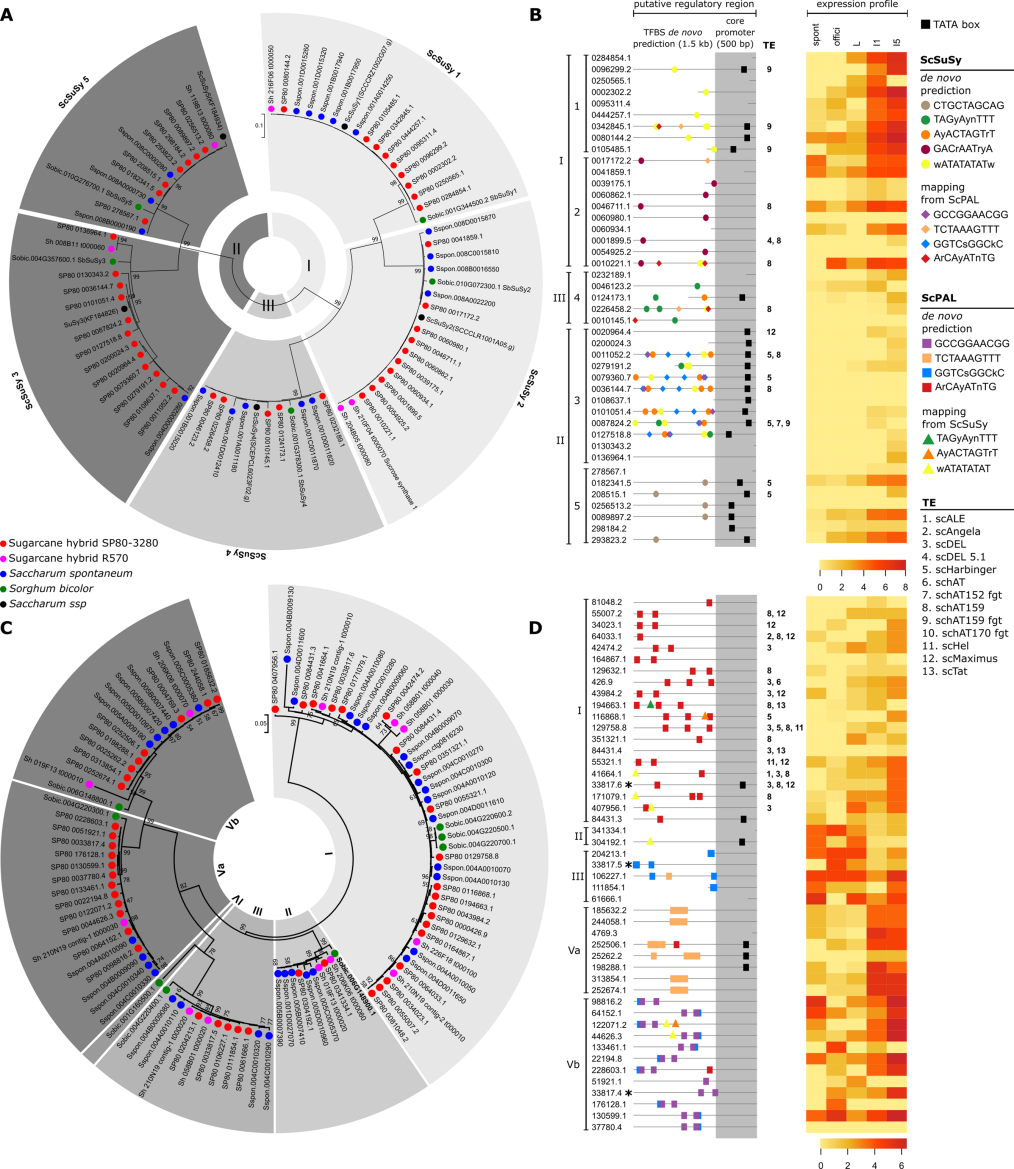
Phylogeny, putative regulatory regions, and expression of sucrose synthase (SuSy) and phenylalanine-ammonia lyase (PAL) gene family. Phylogenetic analysis of (**A**) SuSy and (**C**) PAL genes from SP80-3280, R570, *S. spontaneum*, and sorghum. SuSy sequences from *Saccharum* ssp. [36] were also included. For both SuSy and PAL, nucleotide sequences (CDS) were aligned with CLUSTALW [37] software in MEGA 7.0 [38] and maximum likelihood trees were constructed with 1,000 bootstraps. Core promoter analysis (gray columns in B and D) using TSSPlant [39] suggests ScSuSy2 (**B**) and most ScPAL (**D**) as TATA-less (absence of black squares). Transcription factor binding site (TFBS) prediction (colored symbols in **B** and **D**) using MEME [40] and MotifSampler [41] suggests specific motif for each group (ScSuSy1, ScSuSy2, and ScSuSy5 and PAL I, PAL III, PAL Va, and PAL Vb). The three SP80-3280 PAL genes marked with an asterisk in **D** are present in the same contig. Transposable elements (TEs) were identified within 10 kb upstream from the gene (**B** and **D**). Heat map analysis of RNA-Seq data [29] (expression profile in **B** and **D**) shows more pronounced expression in SP80-3280 internodes (I1 and I5) of ScSuSy1, ScSuSy2, ScSuSy5, and PAL from group V. RNA-Seq of leaf tissues (L) indicates more pronounced expression of ScPAL from groups II and III. ScSuSy3 presents high numbers of TFBS and TE and low expression in all samples.hylogeny, putative regulatory regions, and expression of sucrose synthase (SuSy) and phenylalanine-ammonia lyase (PAL) gene family. Phylogenetic analysis of (**A**) SuSy and (**C**) PAL genes from SP80-3280, R570, *S. spontaneum*, and sorghum. SuSy sequences from *Saccharum* ssp. [36] were also included. For both SuSy and PAL, nucleotide sequences (CDS) were aligned with CLUSTALW [37] software in MEGA 7.0 [38] and maximum likelihood trees were constructed with 1,000 bootstraps. Core promoter analysis (gray columns in B and D) using TSSPlant [39] suggests ScSuSy2 (**B**) and most ScPAL (**D**) as TATA-less (absence of black squares). Transcription factor binding site (TFBS) prediction (colored symbols in **B** and **D**) using MEME [40] and MotifSampler [41] suggests specific motif for each group (ScSuSy1, ScSuSy2, and ScSuSy5 and PAL I, PAL III, PAL Va, and PAL Vb). The three SP80-3280 PAL genes marked with an asterisk in **D** are present in the same contig. Transposable elements (TEs) were identified within 10 kb upstream from the gene (**B** and **D**). Heat map analysis of RNA-Seq data [29] (expression profile in **B** and **D**) shows more pronounced expression in SP80-3280 internodes (I1 and I5) of ScSuSy1, ScSuSy2, ScSuSy5, and PAL from group V. RNA-Seq of leaf tissues (L) indicates more pronounced expression of ScPAL from groups II and III. ScSuSy3 presents high numbers of TFBS and TE and low expression in all samples.

Sucrose synthases (SuSy) catalyze the reversible breakdown of sucrose into uridine diphosphate glucose and fructose in carbon partitioning [35]. In agreement with previous work on sugarcane progenitors [36] (*S. officinarum, Saccharum robustum*, and *S. spontaneum*), 43 ScSuSy (sugarcane sucrose synthase) CDSs identified in the SP80-3280 assembly branch out in phylogenetic inferences as 5 SuSy genes (hereafter ScSuSy1–5) organized in 3 groups: I (ScSuSy1 and 2), II (ScSuSy3 and 5), and III (ScSuSy4) (Fig. 4A). Sorghum shares these 5 SuSy genes, indicating that they evolved before the sugarcane/sorghum divergence. RNA-sequencing (RNA-Seq) data from leaves and internodes of SP80-3280 (Ion PGM Sequencing) [29] shows expression of 34 of the 40 ScSuSy members, suggesting that ScSuSy1–2 (Group I) and ScSuSy5 might control carbon flux from source to biomass conversion in stems, as they show higher expression in internodes than in leaves (Fig. 4B).

Different members of the SuSy gene family may play different functional roles, and in sugarcane this was observed as different expression levels related to different transcription factor binding sites (TFBSs) identified. We identified 5 different top-ranked TFBSs (with the highest score) in the ScSuSy1–5 members. Three of them are related to auxin and abscisic-acid hormone signaling (ScSuSy1, 3, 5). For ScSuSy1 genes, the TFBS analysis predicted the motif wATATATATw (MA1184.1) that is associated with RVE1, a morning-phased transcription factor integrating the circadian clock and auxin pathway genes that bind to the evening element of promoters [42]. For ScSuSy2 genes, we found the motif GACrAATryA (MA1374.1) that is associated with INDETERMINATE DOMAIN transcription factor, which regulates photoperiodic flowering by modulating sugar transport and metabolism [43]. For ScSuSy3 genes, we found the AyACTAGTrT (MA0930.1) motif in 64% of its SP80-3280 copies and in all copies in the *S. spontaneum* and R570 monoploid genomes. It is associated with abscisic acid (ABA)-responsive elements (ABREs) that regulate stress response via ABA signaling. For ScSuSy4 genes, we found the TAGyAynTTT (MA1012.1) motif that is probably involved in regulation of the photoperiod and vernalization pathways. Finally, for ScSuSy5 genes, we found a CTGCTAGCAG (MA0564.1) conserved motif exclusively for ScSuSy5 genes in SP80-3280. This motif allows binding with an element associated with ABI3, which participates in ABA-regulated gene expression. Previous studies from our group had already pointed out ABA- and sucrose-induced genes associated with higher sucrose content in sugarcane [44].

SuSy produces the substrate for cellulose biosynthesis (uridine diphosphate glucose) and is commonly associated with cell wall and cellulose synthesis [45,46]. In view of the myriad of possibilities to convert lignocellulosic compounds into chemicals and fuels, defining phenylpropanoid biosynthesis pathway members in sugarcane is of great interest. Phenylalanine ammonia-lyase (PAL) is the first enzyme in phenylpropanoid biosynthesis [47–49], and silencing its expression has been associated with a reduction in lignin content [47–49]. Lignin is a major component of plant cell walls [18] and sugarcane PAL expression is responsive to the ethylene-releasing ripener (ethephon) in both leaf and internode [50].

Mapping of predicted proteins from SP80-3280 against the SUCEST-FUN Cell Wall Catalogue [51] (731 transcripts of 20 protein categories) identified 3,054 similar proteins (Supplemental Table S5), including 47 PAL copies. Based on a Maximum Likelihood gene tree that includes sorghum, *S. spontaneum*, and mosaic monoploid R570 PAL sequences reveals 5 clusters (Fig. 4C), each containing ≥1 representative with a sorghum ortholog. *S. spontaneum* has 33 putative PAL genes, somewhat more than expected considering that the sequenced genotype is a tetraploid. The higher number may be due to expansion of PAL members in group I that occurred also for sorghum and the sugarcane hybrid genomes of R570 and SP80-3280. Group V has a higher number of SP80-3280 PAL members, and all except 1 (ID 37 780.4) showed expression evidence (Fig. 4D).

Regarding TFBS prediction within PAL regulatory sequences, we identified 4 different top-ranked TFBSs. For PAL I, an ArCAyATnTG (MA0930.1) element was predicted, which is associated with ABF3, a transcription factor involved in ABA and stress responses and acting as a positive component of glucose signal transduction. For PAL III, we found the element GGTCsGGCkC (MA0992.1), an element associated with AP2/ERF, a transcription factor involved in the regulation of gene expression by stress factors and by components of stress signal transduction pathways. For PAL Va, we found the element TCTAAAGTTT (MA0064.1), which is associated with PBF, a transcription factor involved in ABA, stress response, and components of stress signal transduction pathways. Finally, for PAL Vb, we found the motif GCCGGAACGG (MA1009.1). This element is associated with ARF3, a transcription factor involved in auxin and ABA-regulated gene expression. In summary, our results corroborate reported findings [44] that reveal that PAL genes were induced by ABA.

In addition to PAL members' expansion in group I, the CCR (cinnamoyl-CoA reductase), COMT (caffeic acid 3-O-methyltransferase), and 4CL (4-coumarate-CoA ligase) gene families, also related to phenylpropanoid biosynthesis, have much higher numbers of genes (620, 453, and 375, respectively) in sugarcane than sorghum [52] (44, 41, and 15, respectively). This is another challenge and opportunity for future functional characterization (Supplemental Table S6).

The sheer number of sugarcane genes found so far, the large size of multi-gene families, and the evidence that not all homo(eo)logs are expressed point to a very complex role of regulation in the determination of phenotypic differences. Consistent with the gene copy-richness of sugarcane, we inferred 15,737 transcription factors from 57 families (Supplemental Table S7), versus ∼2,000 previously estimated [53]. The classification of core promoters and identification of TFBSs in proximal promoters was performed *in silico*, and the percentage of core promoter regions with a TATA-box element was 47.72% and 12.76% for SuSy and PAL genes, respectively.

The TFBS identification pointed to a wealth of regulatory elements differentially distributed among members of the same gene family, i.e., SuSy and PAL (Fig. 4B and 4D and Supplemental Table S8). In addition, using gene expression data of SP80-3280 plants grown in field conditions for 13 months, we have found evidence of a co-expression module, enriched for phenylpropanoid and lignin biosynthesis gene ontology terms (Supplemental Fig. S5A). This module comprises 116 transcripts, including 1 PAL (Supplemental Fig. S5B), whose expression is higher in internodes 5 and 9 than in leaves and immature internode (Supplemental Fig. S5C). It was possible to identify the TFBSs, predicted as putative regulators of the PAL gene family (Fig. 4D) within the upstream region of these co-expressed genes, suggesting that ABF, ERF, ZF-HD/C2H2, and ARF3 (Supplemental Fig. S5D) may also regulate other genes involved in lignin biosynthesis and metabolism. The most significant motifs found for each gene family (SuSy and PAL) were mapped to the promoter region of the remaining sequences from both SP80-3280 and R570 hybrids and *S. spontaneum* (Supplemental Tables S8 and S9). Interestingly, only ScSuSy2 and ScSuSy3 motifs mapped in all species, suggesting that SP80-3280 holds particular regulatory elements involved in sucrose synthesis. Conversely, SP80-3280 and *S. spontaneum* share all predicted motifs for PAL genes (Supplemental Table S9), suggesting that this gene family may be derived from the *S. spontaneum* ancestor.

### Transposable element insertions may affect SuSy and PAL expression

Fewer transposable elements (TEs) were identified in SP80-3280 gene space than in the AP85-441 *S. spontaneum* and mosaic monoploid R570 assembly, probably due to repetitive regions collapsing in the assembly even with the use of long synthetic-read sequencing (Supplemental Fig. S6, Supplemental Table S10). All previously described TE families are represented in the 3 genome assemblies, disclosing few cultivar-specific amplifications. The 2 modern cultivars (SP80-3280 and R570) have fewer TE counts than the *S. spontaneum* progenitor in normalized monoploid genomes. Long terminal repeat (LTR) retrotransposons are large contributors to genome composition at the chromosome assembly level. However, scMaximus (Copia) and scDel (Gypsy) LTR-retrotransposon families are similarly represented in both gene space and chromosome assemblies, supporting their presence in transcriptionally active regions [54]. We also note that scCACTA transposons are more represented at the gene space assembly than schAT while the scMutator family is similarly represented in both.

Functionally important TE insertions were identified in the ScSuSy gene family (Fig. 4). ScSuSy2 copies have a contrasting pattern, most *S. spontaneum* having TE insertions while most SP80-3280 homo(eo)logs do not—although SP80-3280 and *S. spontaneum* share 1 ancient insertion of schAT159 at similar distances from the ATG. ScSuSy3 genes are polymorphic between species and within SP80-3280, with 6 copies having no TE and 5 in which different TEs may affect expression. In particular, scga7_uti_cns_00 20964:7575–17 575 (-) harbors a full LTR at 280 bases from the ATG. Most ScSuSy4 copies have no TE insertion, but interestingly, as described for ScSuSy2, SP80-3280 (scga7_uti_cns_022 6458:7638–16 073 [-]) and *S. spontaneum* (Chr1B:33 406 669–33 416 669 [-]) share 1 ancient schAT159 insertion. Finally, ScSuSy1 has similar patterns of TE presence and absence in both genomes, and ScSuSy5 genes have no insertions in the promoter regions of either *S. spontaneum* or SP80-3280. Furthermore, PAL genes from group I exhibit most of the copy variation and harbor TEs inserted near the promoter region. Only 2 copies from SP80-3280 and *S. spontaneum* lack TE insertion in PALs from group I.

### Sugarcane and sorghum polymorphisms support recent allotetraploidy and suggest candidate genes for morphological and physiological differences between these taxa

**Figure 5:**
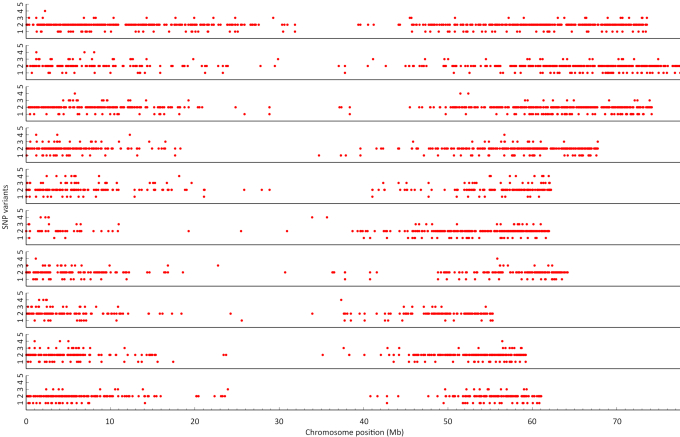
SNVs. Alignment of sugarcane contigs to the genic regions of sorghum chromosomes (chromosome 1 is on top and 10 is at the bottom). X and Y axes indicate physical distance on each chromosome (Mb) and the number of SNVs compared to the sorghum reference genome, respectively. Each dot indicates sorghum genes matching ≥2 sugarcane contigs.

Despite a common foundation for evolving high sugar content with similar SuSy genes (ScSuSy1–5), sugarcane and closely related sorghum have taken different paths since sharing ancestry. We identified 10,586 natural single-nucleotide polymorphism variations (SNVs) between sorghum and sugarcane in 4,140 unique genes, mostly bi-allelic (80.8%), but 6.2% tri-allelic and 0.97% tetra-allelic (Fig. 5). The overwhelming predominance of bi-allelic variations indicates that many sorghum genes are represented by 2 discernible sugarcane copies, supporting the theory of allotetraploidization shortly after divergence from sorghum ∼3.8–4.6 MYA [55], creating 2 sugarcane “subgenomes.” Recently published results from Vieira et al. [56] demonstrate that sugarcane meiotic chromosomes behave as bivalents, supporting this inference. Autotetraploidization after *Saccharum* speciation ∼3.1–3.8 MYA may have further contributed to allelic richness within each sugarcane subgenome. The preservation of as many as 4 functionally different alleles at a locus, with cases observed on all except 1 chromosome (Chr 10; Fig. 5), is consistent with the well-known heterozygosity of sugarcane cultivars and associated susceptibility to inbreeding depression. However, genes for which sugarcane has only 1 allele are more abundant than 3- or 4-allele genes, perhaps reflecting cases in which a single gene copy is sufficient or in which occasional exchanges between subgenomes have homogenized multiple homo(eo)logs.

Furthermore, 1,334 SNVs that differentiate sugarcane from sorghum in 585 single-copy genes in diploid grasses include frameshifts, premature termination, erroneous splicing, loss of stop codons, and incorrect translation initiation (Supplemental Fig. S7 and Supplemental Table S11). These genes are significantly enriched in transcription, DNA-dependent cell organization and biogenesis functional categories in the nucleus and endoplasmic reticulum (Supplemental Table S12) comprising a rich slate of candidates for causes of morphological and physiological differences between these taxa.

### The gene space contribution towards a chromosome-level assembly of a sugarcane commercial hybrid

Notwithstanding the fragmented nature of our assembly, we explored how it could contribute beyond the gene space toward a whole-genome assembly of the hybrid sugarcane genome. Previous analysis of grass genomes revealed extensive conservation of gene order overlaid with a background of small-scale chromosomal rearrangements and numerous localized gene deletions, insertions, and duplications [57]. Recently published estimates of the levels of gene synteny between *Sorghum bicolor* and the sugarcane cultivar R570 found that 83% of the genes are arranged co-linearly in the 2 genomes [13]. In our assembly of SP80-3280, 79,094 (17.6%) contigs had ≥2 predicted genes and could therefore be used to compare the order of genes in SP80-3280 to those of sorghum. To avoid the need to resolve multiple comparisons to duplicated regions in the sorghum genome, we generated a sequence similarity-based clustering of all CDSs from both genomes and used the genes in clusters with only 1 sorghum gene as anchors to evaluate synteny (Supplemental Fig. S8). We found that 9,319 (2.1%) SP80-3280 contigs had ≥2 synteny anchors and 85% (7,906; 1.8% of all contigs) of these contigs were fully syntenic (Supplemental Fig. S9A and B), i.e., had all genes in the same order and orientation in SP80-3280 contigs and the sorghum chromosomes (Supplemental Table S13). To evaluate the effect of SP80-3280 assembly fragmentation on the number of segments with conserved gene order (“syntenic blocks”) per contig, we used a Monte Carlo method to simulate the fragmentation of the chromosomes and contigs of the *Saccharum* R570 and *S. spontaneum* genomes. We performed 1,000 rounds of simulation for each genome and, at each round, sampled 10,000 random fragments from each of these 2 genomes, while simultaneously sampling the same number of contigs from SP80-3280’s assembly. Sampled contigs and contig fragments were constrained to follow the distribution of the number of genes per contig observed for the full SP80-3280 assembly. The number of syntenic blocks on each fragment was then evaluated, and the relative frequency of contigs/fragments per number of syntenic blocks is shown in Supplemental Fig. S10C. We observed that contigs and fragments harboring a single syntenic block are sampled at similar frequencies in all genomes analyzed. While an increase in sequencing coverage would lead to improved estimates of co-linearity, our analysis of the small subset of contigs with ≥2 marker genes suggests that levels of genomic rearrangement in SP80-3280 are similar to those expected anywhere in the genomes of the other 2 *Saccharum* species.

**Figure 6:**
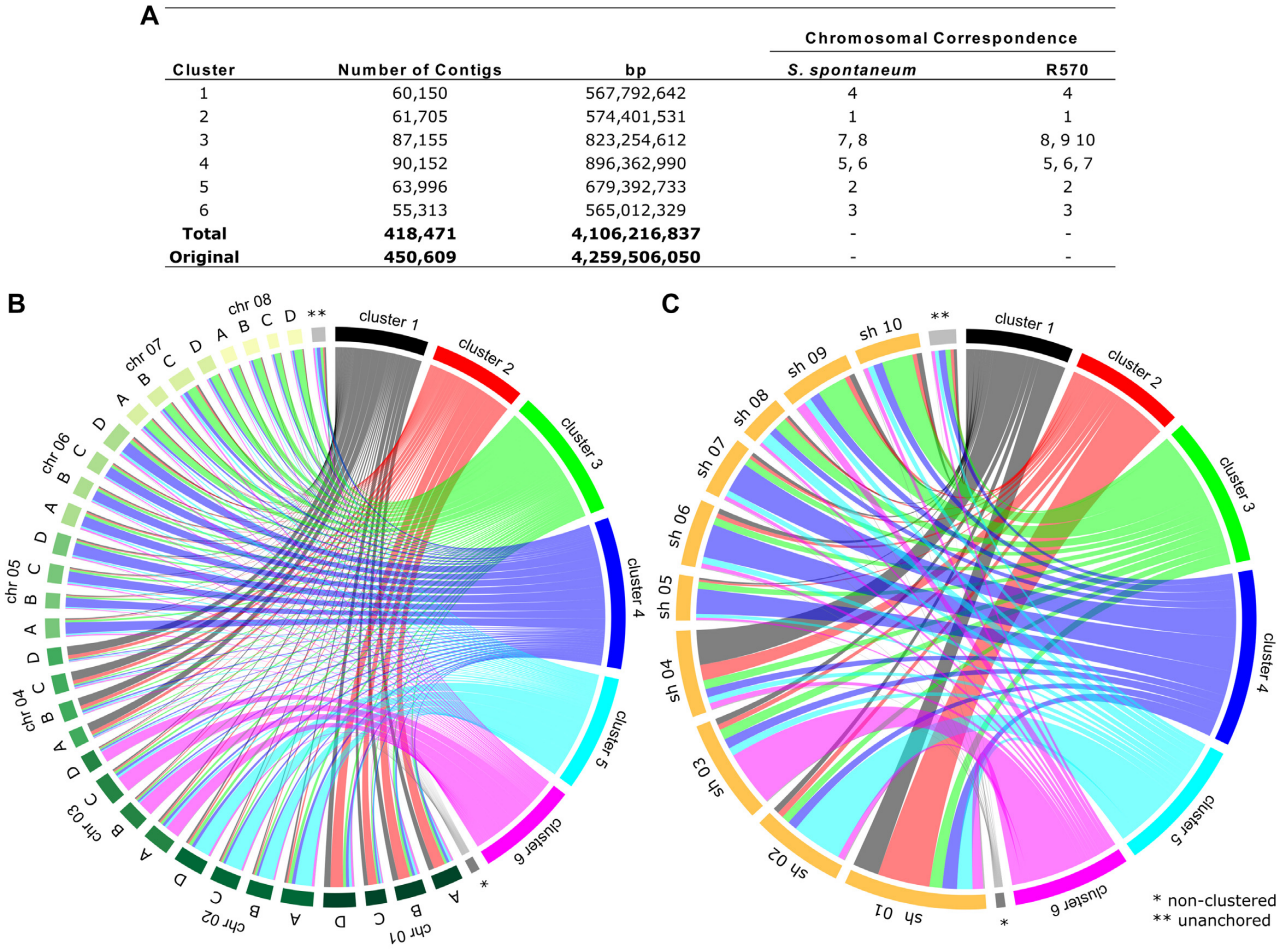
Pseudoassembly of contigs. Multiple correspondence analysis (MCA) with hierarchical clustering of the SP80-3280 assembly against the *S. spontaneum* tetraploid AP85-441 homo(eo)log-resolved assembly [14] and the R570 [13] monoploid genome. **A**: SP80-3280 contigs best hits against AP85-441 and R570 chromosomes and corresponding size of the preliminary scaffolds; cluster = hierarchical cluster from the MCA. **B** and **C**: Circos plot of the proportion of proteins from SP80-3280 (classified into 1 of the 6 clusters or as “non-clustered”) that align to the AP85-441 (chr 01-08) and R570 (sh 01-10) putative chromosomes, respectively.

Finally, to allocate the gene space into potential physical groupings we aligned the SP80-3280 TE masked BWA-SW to chromosome level assemblies of the *S. spontaneum* tetraploid AP85-441 genome [14] and the R570 [13] monoploid genome data. Multiple correspondence analysis (MCA) with hierarchical clustering of the sequences enabled us to allocate the gene space contigs into 6 clusters, an important contribution to future scaffolding efforts. From the total of 450,609 contig sequences, 418,471 (92.86%) produced a BWA-SW alignment against the *S. spontaneum* [14] and R570 [13] assemblies (Fig. 6A) and protein alignment among these 3 species is consistent with MCA results (Fig. 6B and C). Contigs were also mapped against a collection of 778 targeted sequenced bacterial artificial chromosomes (BACs) of which 347 are from SP80-3280 and 431 from R570. All BACs had a corresponding contig match against the assembly. This collection shows that centromeric regions and non-TE multigene families are the most covered (64×). An R gene locus (I2C-2) found in cluster 3 of SP80-3280 and in chromosome 9 of R570 was verified for co-location with a Ca^+^-dependent kinase, a *dog1* (delay of germination 1), and an aminotransferase. The co-location was confirmed in R570 and SP80-3280 BACs showing up to 8 copies of each gene (Supplemental Fig. S10).

## Discussion

This assembly presents 373,869 genes. The gene space described here represents a significant step in understanding the haplotype origin of the hybrid genome. Approximately 12.25% of the SP80-3280 genome sequence is of *S. spontaneum* origin [14], supporting previous studies [10,11]. The comparison against different sets of genes (sorghum, CEGMA, BUSCO, mitochondrial, and chloroplast) shows that the gene space assembly contains the majority of the genes queried in ≥1 copy. The total of predicted genes (373,869) is ∼10×, 14×, and 13× higher than those for monoploid genome assemblies of *S. spontaneum* [14], sugarcane R570 [13], and sorghum [58], respectively. We also detected that single-copy genes in diploid grasses are present in mostly 2–6 copies (up to 15) copies. These findings agree with the predicted 8–14 copies for *S. spontaneum*, depending on the cytotypes, and for modern sugarcane varieties [59]. The total number of predicted genes, the high quality of alignments, and the detection of >1 copy for single-copy genes in diploid grasses indicates that the assembly provides homo(eo)logous resolution for a large fraction of the gene space (∼87%).

Although for sugarcane modern varieties we expect ≥8 copies of each chromosome, it is possible that each homolog does not contain a copy of every gene, because of potential gene loss. In addition, it is also possible that some homeologs were not identified in our assembly because of assembly or sequencing difficulties in regions with highly repetitive sequences. Single-copy genes from diploid grasses correspond to mostly 2–6 copies (up to 15) of sugarcane genes in our SP80-3280 assembly, and nucleotide differences are present mainly in the upstream regulatory region. This highlights the importance and complexity of studying homo(eo)log expression in sugarcane and adds great value to the development of molecular markers for breeding in gene promoter regions. The differences in gene upstream sequences may potentially affect the expression level among the copies and across the studied tissues. This was also reported for the polyploids cotton [60] and wheat [61]. Expression differences among homo(eo)logs in polyploid species may play a crucial role in increasing adaptability to environmental stresses (such as salinity [62], heat, and drought [63]) and in improving performance of new cultivars. These differences highlight the importance of our assembly, which discriminates homo(eo)logs for most genes, e.g., providing important information for the selection of target sequences (genes or promoters) to produce transgenic sugarcane plants. With the homo(eo)logs identified, one could discard a sequence that is not expressed or use genome editing tools to modify a target sequence to increase its expression. It is also possible to identify the progenitor contributing a homo(eo)log (e.g., *S. spontaneum, S. officinarum*, or a parent in a cross) and select the homo(eo)log from the progenitor that has the phenotype of interest.

In an attempt to organize the contigs, we allocate them in 6 clusters using MCA with hierarchical clustering of the sequences. The majority of proteins predicted from chromosomes 1, 2, 3, and 4 (in both *S. spontaneum* and R570) have their best matches located in SP80-3280 contigs from clusters 2, 5, 6, and 1, respectively (Fig. 6B and C). On the other hand, clusters 3 and 4 contain contigs matching to multiple chromosomes, including those in which chromosomal rearrangement events were demonstrated in comparison to sorghum: SsChr5, SsChr6, and SsChr7 from *S. spontaneum* [14] and 6 R570 hom(oe)ology groups, HG5–HG10 [13].

Assembling the genome of a polyploid interspecific hybrid is of especially high value for breeders. The assembly, gene prediction, and annotation provided can bridge long-standing knowledge gaps, allowing them a more efficient use of genomic tools. Sugarcane's large autopolyploid genome, predominant clonal propagation, and need for extensive phenotyping to determine breeding values have contributed to the relatively slow (∼1% per year at most) rate of progress in improvement of sugarcane [64] and perhaps other autopolyploids. The demonstration that most of its many homo(eo)logs are expressed, often with tissue specificity, and that TFBSs and TE insertions differ among homo(eo)logs, suggests complex constraints that may necessitate unusual richness of information to enable effective decisions to be made about selecting some homo(eo)logous alleles at the expense of others in autopolyploid breeding populations. These principles may apply widely to many plants with large polyploid genomes, including many of those most efficient at converting solar radiation to biomass.

The present work discloses a large collection of gene space homo(eo)log diversity, taking advantage of novel sequencing technologies, adding >3 Gb of sequence not previously reported, in addition to genome annotation, data-mined homo(eo)logs, and explored regulatory regions of SuSy and PAL. The presented gene space of the sugarcane genome is a fundamental step towards a high-quality chromosome resolved assembly from a current commercial hybrid. The genome sequence released for this interspecific polyploid supports its recent allotetraploid nature and reveals differences in promoter regions associated with a diverse gene expression pattern and TEs, contributing to fine tuning of the sugarcane genome.

## Methods

### Plant material

Leaves from SP80-3280 were collected and frozen in liquid nitrogen. Genomic DNA was extracted using DNeasy Plant Mini Kit (Qiagen, Hilden, Germany) following the standard protocol. DNA integrity was analyzed using the Agilent High Sensitivity DNA Analysis Kit (Agilent Technologies, Santa Clara, CA, USA) and Agilent 2100 Bioanalyzer Instrument. Quantification was done using Quant-it^TM^ PicoGreen® dsDNA Assay Kit (ThermoFisher Scientific, Waltham, MA, USA) and SpectraMax M2 microplate reader (Molecular Devices, San Jose, CA, USA).

### Sequencing Illumina long-reads and assembly

We used Illumina Synthetic Long-Read sequencing technology (Illumina, San Diego, CA, USA), which provides very accurate long reads with a mean read length of ∼5 kb, thus being able to represent polymorphisms across all copies of chromosomes. Genomic DNA was sheared into 5–10 kb fragments and diluted in a 384-well plate. DNA fragments were ligated with PCR primers and specific sequences, which identify the 5′ and 3′ ends. The fragments from each well were amplified, fragmented, and barcoded with unique indices to create a TruSeq Synthetic Long-Read DNA library. In total, 26 libraries were made. The short fragments created in the second step of fragmentation were pooled and sequenced on the HiSeq instrument at the Illumina Service Genome Network. The reads from each of the 384 wells were pre-processed to correct sequencing and PCR errors. Contigs were produced from the paired-end information and further scaffolded together to resolve repeats and fill in gaps. In this step, the software removes fragments containing inconsistent bases at a higher rate than expected from sequencing error rate. More details on the informatics pipeline for short-read scaffolding into long reads are available in the Fast Track Services Long Reads Pipeline User Guide [65].

To assemble sequences we used a 2-step approach: (i) the Celera Assembler [66] (CA) was used for overlap computation and layout building; (ii) the tig-sense module of the HBAR-DTK from Pacific Biosciences (Pacific Biosciences, Menlo Park, CA, USA) [67] was used to construct consensus sequences. This was motivated by the fact that the CA, which uses the overlap-layout-consensus method, is more robust than de Bruijn graph approaches. However, some adjustments needed to be made. CA, designed for Sanger reads, only accepts quality scores between 0 and 40. Because synthetic long reads are very accurate and some of the base qualities exceeded this upper bound, we set the quality scores >Q40 as Q40 to allow them to be appropriately parsed. The consensus module was also adapted for the analysis of big complex genomes. The substantial number of contigs generated initially (∼450,000, half of them singletons) resulted in several files in a folder that hindered I/O operations. Thus, we (i) modified tig*-*sense to automatically create subdirectories that contained not more than 1,000 contig FASTA files, reducing delays for file lookup; (ii) divided contig processing into non-singletons and singletons, prioritizing non-singleton contigs; and (iii) created a work history so that the program could be resumed after a halt. Overall, these modifications allowed us to reduce the running time of the consensus pipeline by 1 or 2 orders of magnitude. To identify problematic regions, after the assembly step, we have assessed the assembled contigs using a read coverage analysis by mapping reads back to contigs. After sorting contigs from highest coverage to lowest, we found that only 0.1 Gb of contigs had very high coverage (Supplemental Fig. S11).

### Sequencing BAC clones and assembly

A total of 780 independent BACs were sequenced using Roche 454 (Roche, Basel, Switzerland) sequencing technology. Each BAC clone was tagged with a unique barcode, and sets of 12 BACs were pooled in 1 gasket. We assembled BACs individually as described [68] and obtained a total of 49.6 Mb of assembled sequence, with a mean length of 107 kb. The BAC data include 317 R570 BACs [68], 116 additional R570 BACs, and 347 from SP80-3280.

### Assembly validation

#### Comparison with sugarcane BACs

Assembled contigs were aligned against a set of 780 BACs with BWA-MEM (BWA, RRID:SCR_010910), using default parameters. Alignment data were processed for coverage with the aid of SAMTOOLS (SAMTOOLS, RRID:SCR_002105) v1.1 and BEDTools (BEDTools, RRID:SCR_006646) v2.25, and selected matches were ≥10 kb long and covered ≥90% of the contig. Additionally, the unassembled synthetic long reads were aligned to the same set of BACs to check for discrepancies among contigs and long reads, which could be indicative of regions that were not assembled.

#### Comparison with sorghum CDS

The set of 39,207 annotated sorghum CDSs, release version v2.1, were downloaded from Phytozome [69]. These were aligned against the assembled contigs with BLASTn (v2.2.30+) using default parameters. For each sorghum CDS, we identified the longest fraction of the CDS contained within a single unitig. Only hits with ≥80% identity at the nucleotide level were considered for computing coverage. For any CDS with multiple high-scoring segment pairs against the same contig that passed the filtering criteria, we used the union of such hits, excluding any potential overlap. Given that most contigs contained only 1 or 2 genes, we expect very little influence of spurious hits to different gene regions.

#### Comparison with CEGMA

A total of 248 ultra-conservative core eukaryotic genes classified by Korf Lab [23] were assessed in our sugarcane assembly with “-g” and other default options of CEGMA (CEGMA, RRID:SCR_015055) v2.5. To assess the presence of putative homo(eo)logs for CEGMA regions identified on the assembly, the sequences were retrieved according to the coordinates provided on CEGMA output. Sequences were aligned back to the genome using BLASTn with default parameters. Matches with identity and query coverage >90% were considered for calculation of alignment frequency.

#### Comparison with BUSCO

The assembly was assessed for the presence of the 1,440 core genes from the Plantae lineage of BUSCO (BUSCO, RRID:SCR_015008) [24]. BUSCO performs gene prediction and orthogonality assessment using Augustus (Augustus: Gene Prediction, RRID:SCR_008417) [70] and HMMER3 (HMMER, RRID:SCR_005305) [71]. Because these steps demand huge resources, we partitioned sugarcane contigs (4.3 Gb) into 6 groups with similar length and processed BUSCO in parallel. After we merged results, we applied the orthogonality assessment algorithm once again as thresholds that BUSCO exploits to discern actual single-copy orthologs from paralogs.

#### Comparison of the mitochondrial and chloroplast genomes

To reconstruct the SP80-3280 mitochondrial and chloroplast genomes, we have used as reference the complete genomes of *Saccharum* hybrid chloroplast (NC_0 05878.2) [25] and the *S. officinarum* mitochondrial chromosome 1 (LC107874.1) and chromosome 2 (LC107875.1) [26], downloaded from NCBI. The SP80-3280 genome contigs were aligned using BLASTn against their respective references, and the best hits were selected on the basis of cutoff e-value ≤ 1 × 10^−15^, with contig coverage ≥90% and identity ≥70%. The BLASTn alignment results identified 2,482 and 909 contigs for the 2 mitochondrial chromosomes, respectively, and 51,768 contigs for the chloroplast genome. To reconstruct the consensus sequences and perform the genome annotation we used the CLC Genomics Workbench tools (CLC Genomics Workbench, RRID:SCR_011853) [72]. The contigs used for genome reconstruction presented mean size of 4 kb, with coverage depth >20×.

Using the CLC Tools and the Genome Finishing Module, the selected contigs were aligned to their respective references and consensus sequences extracted, filling the gaps with N's. The reconstructed consensus sequence aligned against the chloroplast genome presented 99.99% and 99.99% of coverage and identity, respectively, and there were identified only 6 mismatches and 2 gaps, most of them located in intergenic regions and in one of the rRNA23S copies with protein frame preservation.

The alignment against mitochondrial chromosomes 1 and 2 presented 99.85% and 99.93% of coverage and 99.90% and 99.94% of identity, respectively. The consensus sequences were annotated using their respective NCBI references with the CLC tool “Annotate from Reference,” where all genes, transfer RNAs, ribosomal RNAs (rRNAs), and miscellaneous features were totally transferred. For the mitochondrial chromosome 1, a total of 237 mismatches and 63 gaps were identified, most of them present in intergenic regions and only 2 mismatches in 2 rRNA genes, with protein frame preservation. And for chromosome 2, we identified a region composed by 19 nucleotides inside a repetitive AT region. In addition, the reconstructed chromosome has 57 mismatches and 16 gaps, all of them present in intergenic regions.

#### Comparison with sugarcane ESTs

A set of 134,840 ESTs from leaf, internode, and root samples exclusively from SP80-3280 [20] were aligned to the contig sequences using SPALN v 2.3.3 [33] applying the mapping and alignment algorithm (-Q 5) and admitting all possible matches for each sequence (-M 1000). Coordinates of aligned ESTs were compared to gene annotation using the BEDTools intersect utility [73]. Alignments might be explored through a GBrowse environment [22].

### Genome annotation

#### Gene prediction

Contigs were annotated using a pipeline developed in house, previously used for BAC annotation. TE discovery and masking was done using LTR harvest, LTR digest, CrossMatch against *Utricularia gibba* TE database, and RepeatMasking [74] of Viridiplantae [75] and previously known sugarcane TEs [54].

Genes were discovered and annotated using masked contig sequences. *De novo* predictions were done with Augustus [70], Glimmer HMM (GlimmerHMM, RRID:SCR_002654) [76], GeneMark HMM [77], SNAP (SNAP, RRID:SCR_007936), and PASA (PASA, RRID:SCR_014656) [78] with rice models and sugarcane EST and RNA-Seq data [29]. Alignments were also generated against reference protein databases (sorghum, known sugarcane, and Phytozome) using Exonerate [79] and BLAST [80] (v2.2.30+). Both *de novo* and alignment evidence were used for consensus annotation with EVidenceModeler (EVidenceModeler, RRID:SCR_014659) [81] with greater weight given to experimental and alignment information. Functional assignment was derived from protein database best hits and InterProScan 5 (InterProScan, RRID:SCR_005829) [82] results.

#### GeneOntology annotation

For functional annotation of predicted proteins from SP80-3280, all sequences were aligned to UniRef50 clusters, a dataset of representative sequences clustering high-similarity proteins from UniProtKB [30], using BLASTp (v2.2.30+, e-value 1 × 10*^−^*^5^). Sequences that failed to align in this first approach were also searched against the RefSeq non-redundant protein database. Gene Ontology mapping and annotation of sequences with positive BLAST results was performed using the Blast2Go (Blast2GO, RRID:SCR_005828) framework [83].

### Reference-guided RNA-Seq assembly

We used Trinity (Trinity, RRID:SCR_013048) version 2.0.6 for reassembly of the Sugarcane ORFeome [29] using the genome as a reference, with a minimum contig length of 250 bp (genome_guided_max_intron 3000, genome_guided_min_coverage 5, genome_guided_min_reads_per_partition 10) to identify transcript models. SP80-3280 RNA-Seq reads from 3 tissues (leaves and immature and intermediate internodes) were used for alignment against the reference genome and partitioned into read clusters, which were then individually assembled using Trinity genome-guided methods. Trinity and genome-guided methods used a fixed *k*-mer size of 25 nt. In this new assembly, 269,050 genes and 275,807 transcripts were recovered. The quantity of transcripts recovered by the reference-guided assembly was higher, and thus closer to the number of predicted genes (374,774), than the *de novo* assembly. Transcript expression level was estimated by FPKM (fragments per kilobase of exon model per million reads mapped).

### Identification of putative homo(eo)logs and count estimation

We downloaded the *S. bicolor* genome assembly v2.1 from Phytozome and took 2,051 single-copy genes according to Han et al. [84], which were also present as single copies in the genomes of *Oryza sativa* and *Brachypodium distachyon*. We aligned the CDSs of these sorghum genes to the CDSs of predicted sugarcane genes from the SP80-3280 assembly, using BLASTn (v2.2.30+, e-value 1 × 10^−^^6^). We filtered alignments with ≥80% nucleotide identity, based on Wang et al. [57], covering ≥70% of both the sugarcane and sorghum sequences. Sugarcane gene models aligned to the same single-copy sorghum gene were denoted as putative homo(eo)logs. Finally, we counted the number of copies for each gene.

We clustered all putative homo(eo)logs based on each single-copy sorghum gene to get estimates of sequence differentiation. We aligned the CDSs for each pairwise combination in each gene cluster, using BLAT (BLAT, RRID:SCR_011919) v35 [85] (*–*minIdentity = 0 –minScore = 60). One of the clusters had 21 putative homo(eo)logs, which is higher than the number of chromosome copies expected for sugarcane and was discarded from the analysis. Next, we parsed the alignments to obtain estimates of copy differentiation considering both SNPs and indels. We gathered distance estimates from all pairs, from all clusters, to obtain dissimilarity distributions.

### Putative homo(eo)log characterization

#### Upstream region analysis

We also assessed the dissimilarity levels of regions upstream (potential promoter regions) of the predicted sugarcane putative homo(eo)logs. We initially collected 3 different sequence ranges (100, 500, and 1,000 bp) upstream of the predicted gene start site. Next, we aligned these upstream sequences for each pairwise combination in each cluster, again using BLAT v35 [85] (–minIdentity = 0 –minScore = 30). Finally, for each distance range, we parsed the alignments and computed the dissimilarity level considering both mismatches and gaps to obtain a distance matrix for the upstream region of each cluster. To avoid partial alignments of the upstream sequences, only alignments up to 20% shorter or longer than the expected sequence length were considered. Note that the dimension of the distance matrix varied between gene clusters, according to the distribution of cluster sizes shown in Fig. 2A.

#### Insertions and deletions between gene copy coding sequences

To investigate the occurrence of frameshift mutations between putative homo(eo)logs, we built multiple alignments of its CDSs for each cluster, with MUSCLE (MUSCLE, RRID:SCR_011812) v3.8.31 [86], using default parameters. We then computed the length distribution of insertions and deletions in the CDSs to differentiate between frame-preserving and frameshift insertions and deletions (indels). We parsed the CDS alignment for each pairwise combination of putative homo(eo)logs and counted the number of occurrences of gaps of a given length. We then pooled counts from all copy combinations to get a joint estimated distribution.

#### Tissue-specific homo(eo)log expression analysis

We used RNA-Seq data [29] from leaves (L) and immature (I1) and intermediate (I5) internodes of SP80-3280 to find the expression of putative tissue-specific homo(eo)logs. These reads were initially aligned to the sugarcane genome assembly using TopHat2 (TopHat, RRID:SCR_013035) [87] version 2.0.9 (library-type fr-firststrand). We allowed reads to be aligned to up to 20 contigs of the genome assembly to identify alignments to different homo(eo)logs (–max-multihits 20) and supplied TopHat2 with the putative homo(eo)logs' annotation as a GTF file (–GTF CDSMapping-homo(eo)logs.gtf) in order to direct TopHat2 to align the reads to this transcriptome first.

Besides the TopHat2 alignment, we used the RSEM (RSEM, RRID:SCR_013027) tool rsem-calculate-expression (version 1.2.31) to quantify the expression of predicted genes (bowtie2, fragment-length-mean, fragment-length-sd, and calc-ci parameters). An in-house Perl script was used to estimate the mean length and standard deviation for each RNA-Seq library. The main output of Tophat2 BAM formatted file [88] accepted_hits.bam was used with RSEM to estimate the transcriptome expression profile. We developed in-house Perl and R language (version 3.3.2) scripts to find the number of putative expressed homo(eo)logs for each single-copy gene in diploid grasses, using the information from genome annotation file (GFF format), showing the gene structure, the transcriptome annotation, and respective transcript per million (TPM) abundance. The previous information allowed the creation of the homo(eo)logs GFF file. We also applied TopHat2 to find the number of putative homo(eo)logs expressed only in antisense orientation, using the same protocol described above, and the antisense reads of RNA-Seq previously identified by Nishiyama et al. [29].

### ScSuSy and ScPAL gene family analysis

We used the sugarcane and sorghum SuSy protein sequences reported by Zhang et al. [36] as query for a BLASTx (v2.2.30+) search in the predicted proteins from SP80-3280, *S. spontaneum*[14], and R570 genome assemblies [13]. Putative SuSy genes were then filtered by query coverage ≥80% of ≥1 of the 5 ScSuSy from Zhang et al. [36] and by PFAM [89] domain search, considering only those containing both the conserved sucrose synthase and glucosyl-transferase 1 domains.

Based on BLAST and keyword search (“Phenylalanine ammonia-lyase,” “PAL,” and “EC:4.3.1.24”) in 2 databases (Plant GDB, http://www.plantgdb.org/ and Phytozome [69]) we found 8 different PAL genes in the sorghum genome, the same number previously reported [90]. For sugarcane, PAL genes were retrieved from an EST Cell Wall catalogue [51], which was used as query together with sorghum PAL genes for a BLASTx (v.2.2.30+) search to identify PAL genes in the predicted proteins from *S. spontaneum* [] and R570 genome assemblies [13]. Putative PAL genes were then filtered by query coverage ≥80% of the sorghum PAL genes and by PFAM [89] domain search, considering only those containing the Aromatic amino acid lyase domain. Also, sequences not containing the PAL conserved amino acid motif alanine-serine-glycine [91,92] and an essential Tyr110 [93] were excluded.

For both SuSy and PAL, nucleotide sequences (CDS) were aligned with clustalw [37] software in MEGA (MEGA Software, RRID:SCR_000667) 7.0 [38] and maximum likelihood trees were constructed with 1,000 bootstraps and Gaps/missing data treatment “use all sites.” Expression heat map was constructed using log_2_ TPM from previous RNA-Seq data [29].

### Cell wall−related genes

For the identification of cell wall−related genes in the sugarcane genome we used the Sugarcane SAS Cell Wall catalogue [51] as a reference. The search was carried out using tBLASTn (v2.2.30+, e*-*value 1 × 10^−^^6^). These were manually re-annotated to produce a sugarcane cell wall catalogue with 3,054 sequences, classified in 10 cell wall categories.

### Transcription factor analysis

For the identification and classification of sugarcane predicted proteins into transcription factor families, we used the classification rules and tools described in GRASSIUS [53]. The search was carried out using HMMER v3.1b1 [94], and all significant hidden Markov model (HMM) hits with e-value < 1 × 10^−3^ were kept.

### Promoter region analysis

#### Transcription start site and promoter region classification

We evaluated promoter regions of genes associated with cell wall and sugar metabolism, ScPAL (sugarcane phenylalanine ammonia-lyase), and ScSuSy, respectively, as described above. A total of 47 ScPAL and 44 ScSuSy was used. To extract the candidate promoter region, we selected, when available, up to 1,500 nt upstream from the annotated start position of the gene, consisting of a core promoter (500 nt upstream of the start position) and proximal promoter (1,000 nt upstream of the core promoter). Next, we used TSSPlant [39] to predict the transcription start site (TSS) of the genes and the type of promoter (TATA-box, TATA-less). The software was set to report high score, sense-only TSSs.

#### TFBS in silico characterization

The annotation of TFBSs in the proximal promoter regions was performed in 2 steps: *de novo* prediction of TFBS motifs in smaller subsets of sequences and mapping the predicted TFBSs in the remaining promoter sequences. Sequences were partitioned in 10 subsets: 5 ScPAL groups and 5 ScSuSy groups. We then applied MEME (MEME Suite—Motif-based sequence analysis tools, RRID:SCR_001783) [40] and MotifSampler [41], with default parameters, to each of these datasets to determine putative TFBS motifs. Both were restricted to search for at most 6 motifs with ≤10 nt. MEME candidates were a subset of MotifSampler's. MotifSampler ran for 100 cycles; following the manual we selected, from the 10 top-ranked motifs, the first 5 that occurred ≥10 times in the different cycles. Each of the resulting 35 candidate motifs was searched in the JASPAR public database [95], with partial positive matches for all of them.

To evaluate the significance of the motifs we measured their frequency in promoter regions of each of the original gene families and compared them with the frequency of each of these motifs in the promoter regions of the other SP80-3280 predicted genes. We also mapped the motifs of each ScSuSy and ScPAL gene family, respectively, in the promoter region of the ScSuSy and ScPAL genes from *S. spontaneum* and R570. Candidate motifs were mapped with MotifLocator [41]. For characterizing background sequences, we trained a first-order Markov chain [41] on SP80-3280 coding regions that were previously shuffled using the fasta-shuffle-letters tool [40]. The parameters were set to full match of the motif in the target sequence and score 95% above the background.

### Co-expression analysis

A field experiment was conducted at the Agricultural Sciences Center of the Federal University of São Carlos in Araras (22.31602 S, 47.38929 W) in the state of Sao Paulo, Brazil. Trial plots of SP-3280 consisted of 4 rows 10 m long and spaced 1.35 m apart. The field experiment was initiated in October 2012 and extended until November 2013, representing the conditions under which “1-year” sugarcane crops are cultivated. With the aim of carrying out observations throughout growth and development, tissue samples of the +1 leaves (L1) and upper (I1), immature (I5), and mature (I9) internodes were collected from 2 plots (2 technical replicates) after 4, 8, 11, and 13 months of planting.

RNA was extracted for 4 biological replicates, 2 from each plot, using the TriZol method, treated with DNase I and purified. A pool of samples from leaves and a pool of internodes was used as a “reference sample” for hybridization experiments on a customized 4  ×  44 K oligoarray (Agilent Technologies, Santa Clara, CA, USA) for sugarcane (CaneRegNet), conducted following the recommendations proposed by Lembke et al. [107]. The oligoarrays were read using the GenePix 4000B scanner device (Molecular Devices) and the fluorescence data were processed by Feature Extraction software 9.5.3 (Agilent Technologies, Santa Clara, CA, USA).

Log_2_-transformed expression data were used for discovery and the analysis of co-expression modules, on the CEMiTool R package [96]. The adjacency matrix was calculated by estimating the Spearman's correlation coefficient between all pairs of genes and raised to a soft thresholding power (β) of 14. TopGO (topGO, RRID:SCR_014798) R package [97] was used for gene ontology enrichment analysis for each module, and node and edge files were generated for use with the Cytoscape (Cytoscape, RRID:SCR_003032) network visualization program [98].

### SNV analysis compared to genic regions in *Sorghum bicolor*

The 450,609 sugarcane contigs (183,322 singletons and 267,287 unitigs) were aligned to the sorghum genome sequence [58] using BWA MEM v0.7.10 [99] and contigs with mapping quality >20 were used for variant calling. SNVs were called using samtools v1.1 and bcftools v1.1 [88]. Using in-house Python scripts, extracted SNVs were screened when sugarcane contigs were located on the genic regions of the sorghum genome and ≥2 sugarcane contigs were aligned to the same sorghum gene. Then, the number of SNVs in each gene was counted according to 4-base changes.

SNVs that are homozygous in sugarcane were extracted for further analysis. SNVs mapping to coding regions, splicing sites, stop codons, and transcription initiation sites were classified as potential large-effect SNVs.

#### Functional enrichment test

*Arabidopsis* GO-slim gene annotation was used for functional enrichment analysis. GO-slim terms were assigned to sugarcane genes on the basis of sequence similarity inferred from best BLASTp (v2.2.30+) hit. We used a binomial distribution based on the proportion of a GO-slim term among all annotated genes in the sorghum genome as the null distribution. The binomial test was used to assess functional enrichment, with a significance threshold of *P* > 0.05.

### Conserved synteny blocks

DNA sequences for all CDSs from *S. spontaneum* [14], R570 [13], *S. bicolor* [100], and SP80-3280 were aligned using the BLASTn program. Results from BLAST searches, with e-value ≤ 10^−5^, were parsed using an in-house Python script to filter alignments covering ≥70% of the length of both the query and hit sequences. A second filter, requiring ≥80% identity, was also applied and the resulting pairs of queries and hit sequences were classified into putative orthologous groups using the union-find algorithm. We selected putative orthologous groups present in all 3 organisms but with only 1 *Sorghum* gene to be used as markers to detect blocks of conserved gene order (syntenic bocks) in comparisons of SP80-3280 and *S. spontaneum* against the genome of *S. bicolor*, thus avoiding the complications of a direct comparison of the 2 polyploid genomes (Supplemental Fig. S8). Another Python script was used to detect the syntenic blocks in both *Saccharum* genomes and to count the number of syntenic blocks in each contig. To evaluate the effect of genome fragmentation on our estimates of gene conservation, a Monte Carlo simulation of chromosome fragmentation was performed on the R570 and *S. spontaneum* genomes. We sampled 10,000 random regions of the R570 and *S. spontaneum* genomes, with fragment lengths constrained to follow the distribution of contig lengths observed for SP80-3280. We performed 1,000 rounds of these simulated fragmentations, every time allowing genomic fragments (and the genes within them) to be chosen randomly throughout the genome, with no bias to marker genes. We assessed the degree of conservation through the fraction of contigs with ≥2 marker genes that were found in the same order in the *Saccharum* genome fragments and in the *S. bicolor* genome.

### Chromosome synteny multiple correspondence analysis with clustering

We performed MCA with clustering of the best local alignment hit of masked contigs. Input data were the 450,609 contigs of the sugarcane synthetic long-read assembly and the masked genomic sequences of *S. spontaneum* [14] and R570 [13]. We used the masked sugarcane contig sequence produced by the annotation pipeline, excluding 69,879 sequences that were fully masked.

The contigs were aligned to the grass genomes using BWA-SW v0.7.12-r1044 [99]. We used an in-house Perl 5 script to retrieve the highest-scoring hit for each contig and generate a table for input into R v3.2.1 [101]. This table contained the chromosome hit, if any, for each contig against each reference genome.

We then used the FactoMineR (FactoMineR, RRID:SCR_014602) R package v1.31.3 [102], along with the missMDA missing data–handling auxiliary package v1.8.2 [103]. We performed MCA with these data; i.e., chromosome hit number information for each contig was treated as a set of categorical variables and represented in the 2 principal component dimensions. This was followed by hierarchical clustering in these 2 dimensions, as well as figure rendering, using the Hierarchical Clustering on Principal Components (HCPC) function of FactoMineR.

To identify the correspondence between *S. spontaneum* and R570 chromosomes and SP80-3280 clusters, protein sequence alignment between the cultivar variety and the ancestor and R570 was performed with BLASTp considering an e-value threshold of 1 × 10^−5^. The best hit with a minimum query coverage of 90% was selected for visual representation of the alignment results with Circos plot.

## Availability of Supporting Data and Materials

Genomic data are publicly available at NCBI under GenBank Bioproject PRJNA431722. Contig sequence, gene annotation, alignment with RNA-Seq reads, and SAS are also available in a genome browser framework [22]. The microarray data have been deposited in NCBI's Gene Expression Omnibus and are accessible through GEO Series accession number GSE124990. All data and scripts are also available at GigaDB [104] and in a Github repository [105].

## Additional Files

Fig S1: Illumina long-reads base quality distribution.

Fig S2: Distribution of the largest fraction of each sorghum gene contained in a single sugarcane unitig.

Fig S3: GO classification of predicted genes.

Fig S4: Putative homo(eo)logs expression.

Fig S5: Co-expression analysis.

Fig S6: Comparative TE genome contribution to gene-space and chromosome level assembly.

Fig S7: Distribution of the number of deleterious variations (1,334) and single copy genes (585) containing such variations based on the alignment of sugarcane (SP80-3280) contigs to the genic regions of sorghum chromosomes.

Fig S8: Syntenic block assignment.

Fig S9: Conservation of gene order.

Fig S10: Comparative genomics of I2C-2 locus.

Fig S11: Synthetic long read coverage plot.

Table S1 - Global statistics of two independent SP80-3280 assemblies .

Table S2 - CEGMA gene list.

Table S3 - Busco gene list.

Table S4 - Number of sugarcane transcripts identified through a de novo assembly and a reference-guided assembly.

Table S5 - Cell Wall Metabolism Analysis.

Table S6 - Phenylpropanoid biosynthesis analysis.

Table S7 - Transcription Factor analysis.

Table S8 – De novo TFBS prediction in ScSuSy genes: Mapping in SP80-3280 (SP80), S. Spontaneum (Spon), R570 and all ScSuSy gene putative homo(eo)logs.

Table S9 – De novo TFBS prediction in ScPAL genes: Mapping in SP80-3280 (SP80), S. spontaneum (Spon), R570 and all ScPAL gene putative homo(eo)logs.

Table S10 - Repetitive element analysis.

Table S11 - Single nucleotide variation (SNV) analysis between sorghum and sugarcane (SP80-3280) for single copy genes.

Table S12 - Function enrichment test of genes containing potential deleterious SNVs between sugarcane and sorghum (P<0.05).

Table S13 - Syntenic blocks among SP80-328, R570 and AP85-441 (S. spontaneum), detected using single-copy genes from Sorghum.

giz129_GIGA-D-19-00013_Original_Submission

giz129_GIGA-D-19-00013_Revision_1

giz129_GIGA-D-19-00013_Revision_2

giz129_GIGA-D-19-00013_Revision_3

giz129_Response_to_Reviewer_Comments_Original_Submission

giz129_Response_to_Reviewer_Comments_Revision_1

giz129_Response_to_Reviewer_Comments_Revision_2

giz129_Reviewer_1_Report_Original_Submission Nils Stein -- 3/10/2019 Reviewed

giz129_Reviewer_2_Report_Original_Submission Nathan Watson-Haigh -- 3/11/2019 Reviewed

giz129_Reviewer_2_Report_Revision_1 Nathan Watson-Haigh -- 6/24/2019 Reviewed

giz129_Reviewer_2_Report_Revision_2 Nathan Watson-Haigh -- 9/11/2019 Reviewed

giz129_Supplemental_Figures_and_Tables

## Abbreviations

4CL: 4-coumarate-CoA ligase; ABA: abscisic acid; ABRE: ABA-responsive element; BAC: bacterial artificial chromosome; BLAST: Basic Local Alignment Search Tool; BLAT: BLAST-Like Alignment Tool; bp: base pairs; BUSCO: Benchmarking Universal Single-Copy Orthologs; BWA: Burrows-Wheeler Aligner; CCR: cinnamoyl-CoA reductase; CDS: coding sequence; CEGMA: Core Eukaryotic Genes Mapping Approach; COMT: caffeic acid 3-O-methyltransferase; *dog1:* delay of germination 1; EST: expressed sequence tag; FPKM: fragments per kilobase of exon model per million reads mapped; Gb: gigabase pairs; GO: gene ontology; HBAR-DTK: Hierarchical-Based AssembleR Development ToolKit; HMM: hidden Markov model; indels: insertions and deletions; I2C-2: R gene locus; kb: kilobase pairs; LTR: long terminal repeat; Mb: megabase pairs; MCA: multiple correspondence analysis; nt: nucleotide; NCBI: National Center for Biotechnology Information; ORF: open reading frame; PAL: phenylalanine ammonia-lyase; PASA: Program to Assemble Spliced Alignments; RNA-Seq: RNA sequencing; rRNA: ribosomal RNA; ScPAL: sugarcane phenylalanine ammonia-lyase; ScSuSy: sugarcane sucrose synthase; SNV: single-nucleotide polymorphism variant; SPALN: Space-Efficient Spliced Alignment; SuSy: sucrose synthase; TE: transposable element; TFBS: transcription factor binding site; TPM: transcripts per million; TSS: transcription start site.

## Competing Interests

The authors declare that they have no competing interests.

## Funding

This work was funded by São Paulo Research Foundation (FAPESP) and Microsoft Research (FAPESP grant #2012/51062–3) and São Paulo Research Foundation (FAPESP grants #2016/17545-8, #2014/50921-8, #2008/52146-0, and #2008/52074-0) under the BIOEN Program. Additional funding included awards from the National Science Foundation ( DBI-1350041) and from the National Institutes of Health (R01-HG006677). Bioinformatic tools were run locally on the servers HELIX -IQ/Lab. Signal Transduction—and on the eScience Network—IME/FAPESP grant #2011/50761–2, CNPq, CAPES, NAP eScience—PRP—USP.

G.M.S. is a recipient of a CNPq Productivity Fellowship 304 360/2014–7; M.A.V.S. is a recipient of a CNPq Productivity Fellowship (308 197/2010–0); G.R.A.M. was supported by the FAPESP grant #2015/22993–7; J.W.G. was supported by the FAPESP Fellowships #2013/18322–4 and #2015/15346–5 and CNPq Fellowship 159094/2014–3; A.L.D. is a recipient of a FAPESP Fellowship #2017/02270–6; M.M.O. was a recipient of a CAPES Fellowship DS-1  454337; S.S.F. was supported by the FAPESP Fellowships #2013/23048–9 and 2016/06917–1; M.Y.N. was supported by a FAPESP fellowship #2013/07467–1; F.T.C. is a recipient of a FAPESP Fellowship 2017/02842–0; A.M.D. is a recipient of a CNPq Productivity Fellowship ( 309566/2015–0); A.H.P. is a recipient of funding from the International Consortium for Sugarcane Biotechnology; US National Science Foundation IOS-0115903, and Georgia Agricultural Experiment Station.

## Authors' Contributions

Project leaders: G.M.S., M.A.V.S., and D.H.

Sample collection and DNA extraction: C.G.L.

Genome sequencing and assembly: H.L., M.C.S., G.R.A.M., R.P., and B.D.

Genome assembly supervision: D.H.

Genome annotation: M.A.V.S., J.W.G., M.Y.N., and F.T.C.

*Saccharum spontaneum* genome assembly: J.Z., X.Z., Q.Z., and R.M.

BWA-SW analysis: J.W.G.

BAC sequencing and assembly: M.A.V.S., J.W.G., G.T.R.

Synteny analysis: A.M.D., R.F.S., and G.G.N.

Reference-guided RNA-Seq assembly: M.Y.N.

Tissue-specific allelic expression analysis: M.Y.N., C.G.L., and P.M.A.

Phylogeny analysis: S.S.F. and A.L.D.

SP80-3280 growth and maturation experiment: M.S.C., G.M.S., C.G.L., and A.L.D.

Co-expression analysis: A.L.D.

Regulatory region analysis (TE and TFBS): M.A.V.S., M.M.O., A.M.D., G.M.S., C.T.H., and A.L.D.

SNP variant (SNV) analysis: C.K., H.G., and A.H.P.

Organization and management of the author's contributions: C.G.L., A.L.D., G.M.S., and M.A.V.S.

Data availability (NCBI, GitHub, and SUCEST-fun): F.T.C.

All authors have read and approved the final version of the manuscript.
