# Supplementary material for: Assembly of the 373k gene space of the polyploid sugarcane genome reveals reservoirs of functional diversity in the world's leading biomass crop
Source: Gigascience. 2019 Nov 29;8(12):giz129. doi: 10.1093/gigascience/giz129 (PMC6884061; doi:10.1093/gigascience/giz129)
Supplement: giz129_GIGA-D-19-00013_Revision_3 [file giz129_giga-d-19-00013_revision_3.pdf]

GigaScience

Assembly of the 373K gene space of the polyploid sugarcane genome reveals reservoirs of functional diversity in the world's leading biomass crop

--Manuscript Draft--

**Manuscript Number:**	GIGA-D-19-00013R3
**Full Title:**	Assembly of the 373K gene space of the polyploid sugarcane genome reveals reservoirs of functional diversity in the world's leading biomass crop
**Article Type:**	Research

Funding Information:

FAPESP (2012/51062-3)	Professor Glaucia Mendes Souza
FAPESP (2008/52146-0)	Professor Glaucia Mendes Souza
FAPESP (2014/50921-8)	Professor Glaucia Mendes Souza
FAPESP (2008/52074-0)	Professor Marie-Anne Van Sluys
FAPESP (2011/50761-2)	Not applicable
National Science Foundation (DBI-1350041)	Not applicable
CNPq (304360/2014-7)	Professor Glaucia Mendes Souza
CNPq (308197/2010-0)	Not applicable
FAPESP (2015/22993-7)	Not applicable
FAPESP (2013/18322-4)	Not applicable
FAPESP (2015/15346-5)	Not applicable
CNPq (159094/2014-3)	Not applicable
FAPESP (2017/02270-6)	Not applicable
CAPES (DS-1454337)	Not applicable
FAPESP (2013/23048-9)	Not applicable
FAPESP (2016/06917-1)	Not applicable
FAPESP (2013/07467-1)	Not applicable
FAPESP (2017/02842-0)	Not applicable
CNPq (309566/2015-0)	Not applicable
National Science Foundation (IOS/0115903)	Not applicable
National Institutes of Health (R01-HG006677)	Not applicable
FAPESP (2016/17545-8)	Professor Marie-Anne Van Sluys

Abstract:

Background Sugarcane cultivars are polyploid interspecific hybrids of giant genomes, typically with 10-13 sets of chromosomes from two Saccharum species. The ploidy, hybridity and size of the genome, estimated to have in excess of 10 Gb, pose a great challenge for sequencing. Results Here we present a gene-space assembly of SP80-3280, including 373,869 putative genes and their potential regulatory regions. Their alignment to single copy genes of diploid grasses indicates that we could resolve 2-6 (up to 15) putative homo(eo)logs that are 99.1% identical within their coding

Powered by Editorial Manager® and ProduXion Manager® from Aries Systems Corporation

	sequences. Dissimilarities increase in their regulatory regions and gene promoter analysis shows differences in regulatory elements within gene families and are species-specific expressed. We exemplify these differences for sucrose synthase (SuSy) and phenylalanine ammonia-lyase (PAL), two gene families central to carbon partitioning. SP80-3280 have particular regulatory elements involved in sucrose synthesis not found in the ancestor S. spontaneum. PAL regulatory elements are found in co-expressed genes related to fiber synthesis within gene networks defined during plant growth and maturation. Comparison to sorghum reveals predominantly biallelic variations in sugarcane, consistent with the formation of two 'subgenomes' after their divergence ca. 3.8~4.6 MYA and reveals SNVs that may underlie their differences. Conclusions This gene-copy resolved assembly represents a large step towards a whole genome assembly of a commercial sugarcane cultivar providing a large diversity of genes and homo(eo)logs useful for improving biomass and food production.
**Corresponding Author:**	Glaucia Mendes Souza, Ph.D<br>Universidade de São Paulo<br>Sao Paulo, SP BRAZIL
**Corresponding Author Secondary Information:**	
**Corresponding Author's Institution:**	Universidade de São Paulo
**Corresponding Author's Secondary Institution:**	
**First Author:**	Glaucia Mendes Souza, Ph.D
**First Author Secondary Information:**	
**Order of Authors:**	Glaucia Mendes Souza, Ph.D
	Marie-Anne Van Sluys, Ph.D
	Carolina Gimiliani Lembke, Ph.D
	Hayan Lee, Ph.D
	Gabriel Rodrigues Alves Margarido, Ph.D
	Carlos Takeshi Hotta, Ph.D
	Jonas Weissmann Gaiarsa, Ph.D
	Augusto Lima Diniz, Ph.D
	Mauro de Medeiros Oliveira, Ph.D
	Sávio de Siqueira Ferreira, Ph.D
	Milton Yutaka Nishiyama-Jr, Ph.D
	Felipe ten Caten, Ph.D
	Geovani Tolfo Ragagnin, MSc
	Pablo de Morais Andrade, Ph.D
	Robson Francisco de Souza, Ph.D
	Gianlucca Gonçalves Nicastro, Ph.D
	Ravi Pandya, BS.c
	Changsoo Kim, Ph.D
	Hui Guo, Ph.D
	Alan Mitchell Durham, Ph.D
	Monalisa Sampaio Carneiro, Ph.D
	Jisen Zhang, Ph.D
	Xingtan Zhang, PhD

Powered by Editorial Manager® and ProduXion Manager® from Aries Systems Corporation

	Qing Zhang, Ph.D
	Ray Ming, Ph.D
	Michael Schatz, Ph.D
	Bob Davidson
	Andrew Paterson, Ph.D
	David Heckerman, Ph.D
**Order of Authors Secondary Information:**	
**Response to Reviewers:**	We have uploaded our final version of the manuscript with data citation and updated funding information.
**Additional Information:**	
**Question**	**Response**
Are you submitting this manuscript to a special series or article collection?	No
**Experimental design and statistics**<br><br>Full details of the experimental design and statistical methods used should be given in the Methods section, as detailed in our Minimum Standards Reporting Checklist. Information essential to interpreting the data presented should be made available in the figure legends.<br><br>Have you included all the information requested in your manuscript?	Yes
**Resources**<br><br>A description of all resources used, including antibodies, cell lines, animals and software tools, with enough information to allow them to be uniquely identified, should be included in the Methods section. Authors are strongly encouraged to cite Research Resource Identifiers (RRIDs) for antibodies, model organisms and tools, where possible.<br><br>Have you included the information requested as detailed in our Minimum Standards Reporting Checklist?	Yes
**Availability of data and materials**	Yes

All datasets and code on which the conclusions of the paper rely must be either included in your submission or deposited in publicly available repositories (where available and ethically appropriate), referencing such data using a unique identifier in the references and in the "Availability of Data and Materials" section of your manuscript.<br><br>Have you have met the above requirement as detailed in our Minimum Standards Reporting Checklist?	

Manuscript

Click here to access/download;Manuscript;GIGA-D-19-00013 Manuscriptforpublication.docx

Click here to view linked References

Assembly of the 373K gene space of the polyploid sugarcane genome reveals reservoirs of functional diversity in the world's leading biomass crop

Full name	Institutional address	e-mail
Glaucia Mendes Souza*	1	glmsouza@iq.usp.br
Marie-Anne Van Sluys*	2	mavsluys@usp.br
Carolina Gimiliani Lembke	1	carolina.lembke@gmail.com
Hayan Lee	3,4	hayan.lee@stanford.edu
Gabriel Rodrigues Alves Margarido	5	gramarga@usp.br
Carlos Takeshi Hotta	1	hotta@iq.usp.br
Jonas Weissmann Gaiarsa	2	jonaswg@gmail.com
Augusto Lima Diniz	1	augustold@usp.br
Mauro de Medeiros Oliveira	1	mauromedeiros@usp.br
Sávio de Siqueira Ferreira	1,2	saviobqi@gmail.com
Milton Yutaka Nishiyama-Jr	1,6	yutakajr@gmail.com
Felipe ten Caten	1	ftencaten@gmail.com
Geovani Tolfo Ragagnin	2	geovaniragagnin@gmail.com
Pablo de Morais Andrade	1	pablo.andrade@gmail.com
Robson Francisco de Souza	7	rfsouza@usp.br
Gianlucca Gonçalves Nicastro	7	nicastro@iq.usp.br
Ravi Pandya	8	ravip@microsoft.com,
Changsoo Kim	9,10	changsookim@cnu.ac.kr
Hui Guo	9	huiguo7@gmail.com
Alan Mitchell Durham	11	aland@usp.br
Monalisa Sampaio Carneiro	12	monalisa@ufscar.br
Jisen Zhang	13	zjisen@126.com
Xingtan Zhang	13	tanger_009@163.com
Qing Zhang	13	zhangqing970@126.com
Ray Ming	13,14	rayming@illinois.edu
Michael C. Schatz	3,15	michael.schatz@gmail.com
Bob Davidson	8	bob.davidson@microsoft.com
Andrew Paterson	9	paterson@uga.edu
David Heckerman	8	heckerma@hotmail.com

1 – Departamento de Bioquímica, Instituto de Química, Universidade de São Paulo, Av. Prof. Lineu Prestes, 748, São Paulo, SP 05508-000, Brazil

2 – Departamento de Botânica, Instituto de Biociências, Universidade de São Paulo, Rua do Matão, 277, São Paulo, SP 05508-090, Brazil

3 – Cold Spring Harbor Laboratory, One Bungtown Road, Koch Building #1119, Cold Spring Harbor, NY 11724, United States of America

4 – Department of Energy Joint Genome Institute, 2800 Mitchell Drive, Walnut Creek, CA CA 94598, United States of America

5 – Departamento de Genética, Escola Superior de Agricultura Luiz de Queiroz, Universidade de São Paulo, Avenida Pádua Dias, 11, Piracicaba, SP 13418-900, Brazil

6 – Laboratório Especial de Toxinologia Aplicada, Instituto Butantan, Av. Vital Brasil, 1500, São Paulo, SP 05503-900, Brazil

7 – Departamento de Microbiologia, Instituto de Ciências Biomédicas, Universidade de São Paulo, Av. Professor Lineu Prestes, 1734, São Paulo, SP 05508-900, Brazil

8 – Microsoft Research, One Microsoft Way, Redmond, WA 98052, United States of America9 – Plant Genome Mapping Laboratory, University of Georgia, 120 Green Street, Athens, GA 30602-7223, United States of America

10 – Department of Crop Science, Chungnam National University, 99 Daehak Ro Yuseong Gu, Deajeon, 34134, South Korea

11 – Departamento de Ciências da Computação, Instituto de Matemática e Estatística, Universidade de São Paulo, Rua do Matão, 1010, São Paulo, SP 05508-090, Brazil

12 - Departamento de Biotecnologia e Produção Vegetal e Animal, Centro de Ciências Agrárias, Universidade Federal de São Carlos, Rodovia Washington Luis km 235, Araras, SP 13.565-905, Brazil

13 – FAFU and UIUC-SIB Joint Center for Genomics and Biotechnology, Fujian Agriculture and Forestry University, Shangxiadian Road, Fuzhou 350002, Fujian, China

14 - Department of Plant Biology, University of Illinois at Urbana-Champaign, 201 W. Gregory Dr. Urbana, Urbana, Illinois 61801, USA

15 – Departments of Computer Science and Biology, Johns Hopkins University, 3400 North Charles Street, Baltimore, MD 21218-2608, United States of America

[*]These authors contributed equally to this work and are co-corresponding authors: glmsouza@iq.usp.br and mavsluys@usp.br

ABSTRACT

Background

Sugarcane cultivars are polyploid interspecific hybrids of giant genomes, typically with 10-13 sets of chromosomes from two Saccharum species. The ploidy, hybridity and size of the genome, estimated to have in excess of 10 Gb, pose a great challenge for sequencing.

Results

Here we present a gene space assembly of SP80-3280, including 373,869 putative genes and their potential regulatory regions. The alignment of single-copy genes in diploid grasses to the putative genes, indicates that we could resolve 2-6 (up to 15) putative homo(eo)logs that are 99.1% identical within their coding sequences. Dissimilarities increase in their regulatory regions and gene promoter analysis shows differences in regulatory elements within gene families and are species-specific expressed. We exemplify these differences for sucrose synthase (SuSy) and phenylalanine ammonia-lyase (PAL), two gene families central to carbon partitioning. SP80-3280 have particular regulatory elements involved in sucrose synthesis not found in the ancestor *S. spontaneum*. PAL regulatory elements are found in co-expressed genes related to fiber synthesis within gene networks defined during plant growth and maturation. Comparison to sorghum reveals predominantly biallelic variations in sugarcane, consistent with the formation of two ‘subgenomes’ after their divergence ca. 3.8~4.6 MYA and reveals SNVs that may underlie their differences.

Conclusions

This assembly represents a large step towards a whole genome assembly of a commercial sugarcane cultivar. It includes a rich diversity of genes and homo(eo)logous resolution for a representative fraction of the gene space, relevant to improve biomass and food production.

Keywords: Allele; Bioenergy; Biomass; Genome; Polyploid

BACKGROUND

Sugarcane is the world's most cultivated crop in tonnage (more than rice, maize and wheat) [1], and is considered the most sustainable of energy crops [2] with high potential to mitigate climate change without affecting food security [3]. Already produced in over 100 countries, high productivity of sugar, bioethanol and bioelectricity [4] make it a highly expandable green alternative to petroleum [5–7]. The International Energy Agency projects a 150 EJ (17% of energy demand) contribution of bioenergy by 2060, delivering 18% of the emission reductions needed to achieve the 2DS (2°C Scenario). Sugarcane bioenergy production by 2045 could displace up to 13.7% of crude oil consumption and 5.6% of the world's CO_2 emissions relative to 2014. This can be achieved without using forest preservation areas or land necessary for food production systems. Additionally, the myriad of products that can derive from sugarcane biomass [8] further enhance opportunities for sugarcane in a portfolio of technologies needed to transition to a low carbon 'bioeconomy'.

Opportunities to accelerate breeding progress and enrich knowledge of the fundamental biology of this important plant motivate efforts to produce a high-quality reference genome, a challenge that is unusually complex. Unlike wheat cultivated species known to be either tetraploid (AABB) or hexaploid (AABBDD), the *Saccharum* (sugarcane) genus is considered to be a species complex. A recent study [9] proposed independent polyploidization events within *Saccharum* after divergence from the last ancestor shared with *Sorghum*, superimposed upon an additional whole genome duplication since the diversification of grasses. As a consequence, the sugarcane genome is redundant and harbors genes in multiple functional copies. Adding further complexity, sugarcane cultivars are polyploid/aneuploid interspecific hybrids, typically with 10-13 sets of their 10 basic chromosomes, 80-85% from *Saccharum officinarum* (2n=80), which is known for its sweetness, 10-15% from *S. spontaneum* (2n=40-128) known for its robustness, and ~5% with recombined chromosomes between those two progenitors [10,11]. The ploidy, hybridity and sheer size of the genome, estimated to have in excess of 10 Gb, pose a great challenge for sequencing [12]. Recently released sequences of the modern cultivar R570 yielded a mosaic monoploid reference (382 Mb single tiling path) [13] and a *S. spontaneum* AP85-441 haploid assembly (3.13 Gb) [14].

Worldwide sugarcane yield (~84 ton/ha) is currently only ~20% of the theoretical potential (~381 ton/ha), spurring great interest in conventional or molecular breeding approaches to improve it. However, progress by conventional breeding towards closing the gap between current and potential yield has been slow with gains in the order of 1.0–1.5% a year [15]. Sugarcane commercial cultivars distribute roughly one third of their

carbon into sucrose and two thirds into tops and stems which, due to high lignin content, are burned to fuel boilers, contributing to the favorable energy balance of industrial processes [16]. As sugarcane can accumulate large amounts of sucrose in its stems, up to ~650 mM [17], it is important to study sucrose metabolism and the key players in its regulation. Also, of interest is the revealing of regulators of cell wall biosynthesis. Altering these pathways may help shift carbon partitioning from sucrose storage to biomass accumulation, rich in fiber content, mostly composed of secondary cell walls formed by cellulose, hemicellulose and lignin [18]. The latter compound is a hydrophobic polymer that provides strength and rigidity to the plant, but also is responsible for cell wall recalcitrance, which is the natural plant resistance to hydrolytic attacks that hampers cellulosic ethanol production [19].

RESULTS

The SP80-3280 assembly reveals a gene space of 373,869 genes

Here, we report a representative gene space assembly of the genome sequence of SP80-3280 (GenBank accession number QPEU01000000), the cultivar used in Brazilian breeding programs with the largest collection of transcriptomic data available [20]. In the assembly of 4.26 GB, 373,869 putative genes and promotor regions were predicted. For a large fraction of the gene space, an average of 6 sugarcane haplotypes, putatively homo(eo)logs, were identified. This is the first release of an assembly of such a giant hybrid polyploid genome with part of the putatively homo(eo)logs resolved and their potential regulatory regions.

The assembly was constructed using 26 libraries sequenced using Illumina Synthetic Long-Read technology, obtaining 19 Gb, ~19x haploid genome coverage (~1.9X genome coverage) with >99% of bases having >99% accuracy (**Additional file 1: Fig. S1**), which assure the sequence quality of genes (to be predicted) and intergenic regions (which include the 5' and 3' region of genes). The final assembly includes 450,609 contigs (267,287 unitigs + 183,322 singletons), with average length of 9,452 bp and NG50 of 41,394 bp (**Table 1)**, adding over 3Gb of sequence not previously reported (**Additional file 2: Table S1**) [21]. The gene space described here might be explored through a GBrowse environment available at http://sucest-fun.org/cgi-bin/cane_regnet/gbrowse2/gbrowse/microsoft_genome_moleculo_scga7/.

Comparisons to different sets of genes were performed: (*i*) among 39,441 sorghum transcripts, 39,207 (99.4%) matched the assembly, at least partially; of these, 71.1% matched at least one sugarcane contig with 90% or higher coverage (**Additional file 1: Fig. S2**); (*ii*) the assembly completely covers 217 (87.5%) of the 248 ultra-conserved Core Eukaryotic Genes Mapping Approach (CEGMA) [22] proteins, and partly covers 18 (7.3%), with only 13 (5.2%) not detected (**Additional file 2: Table S2)**; (*iii*) among 1,440 genes in the Benchmarking Universal Single-Copy Orthologs (BUSCO) [23] Plantae lineage, the assembly completely covers 1,309 (90.9%) and partially covers 53 (3.7%) (**Additional file 2:Table S3)**. By including tBLASTn of the 78 (5.4%) missing Plantae lineage BUSCO genes, only 8 (0.5%) are absent; (*iv*) assembled chloroplast (NC_005878.2) and mitochondrial (LC107874.1and LC107875.1) genomes were over 99% similar (at gene level) to published *Saccharum* genomes [24,25]; and (*v*) 94.9% of 134,840 SP80-3280 expressed sequence tags (ESTs) match the assembled gene space sequence.

The assembly revealed 373,869 putative genes with 374,774 transcripts (**Table 1**), far more than the 72,269 unigenes inferred from six sugarcane genotypes [26]; 85,151 transcripts of sugarcane genotypes with contrasting lignin contents [27]; and 195,765 transcripts inferred from *de novo* assembly of ORFeomes from *S. officinarum*, *S. spontaneum* and SP80-3280 [28].

Among the predicted transcripts, 302,627 (80.7%) aligned to a Uniref50 protein [29], and 195,651 were annotated with 10,362 GO terms [30] (**Additional file 1: Fig. S3**). Our previously published SP80-3280 ORFeome was reassembled using the genome as a reference, revealing 269,050 genes and 275,807 transcripts from leaves, immature and intermediate internodes (**Additional file 2: Table S4)**. Further, a set of 134,840 SP80-3280 ESTs from a Sugarcane EST Project – SUCEST [20] – were mapped to assembled contigs and compared to predicted genes, in order to further estimate the homo(eo)logous abundance of the predicted gene space. A total of 127,940 ESTs (92.8%) have at least one match in the assembly, which is in accordance with similar analysis of other plant genomes [31], and only 6.8% of aligned ESTs (8,499) do not correspond with predicted genes. This result resembles the BUSCO results, for which only 5.4% of conserved genes could not be identified in the assembly. Although 10.4% of ESTs (12,966) have a unique hit, what may represent sequencing/assembly issues or genes loss, 84.9% of ESTs (106,133) show 2-8 and up to 30 matches on the genome, reflecting the presence of the majority of putative homo(eo)logs (**Fig. 1A**). This result is similar to

the search of CEGMA matches against the genome itself using BLASTn. From 235 sequences completely or partially covering CEGMA proteins, 205 has 2-8 and up to 17 matches on the genome (**Fig. 1B**).

To verify how the assembled gene space reflected the expected content of homo(eo)logous genes, the gene content was compared to those of other grasses. Single-copy genes in diploid grasses (sorghum, rice and *Brachypodium*) are present in up to 15 copies in sugarcane, mostly with 2-6 copies (total of 1,592 coding sequences (CDS) in sugarcane) (**Fig. 2A**). Dissimilarities among putative homo(eo)logs increase from the coding region to the promoter region, with median divergence of 0.90% between CDS, 1.03% for the 100 nucleotides (nt) upstream, 4.47% for 500 nt and 7.50% for 1,000 nt (**Fig. 2B**). Frame-preserving INDELs are more abundant than frameshifts (**Fig. 2C**) and short frameshift INDELS were relatively less frequent in the sugarcane exons than in sorghum [32].

The SP80-3280 gene series that correspond to single-copy genes in diploid grasses showed expression of sense copies for multiple homo(eo)logs (**Fig. 3A**), with very few copies transcribed in antisense orientation (**Fig. 3B**) based on alignment with the SP80-3280 cDNA reads [28] from leaves, immature and intermediate internodes. For some genes, not all copies are expressed in SP80-3280 (**Fig. 3A, Additional file 1: Fig. S4 A**). In addition, the increase in the number of expressed copies is not accompanied by an increase in the level of expression (**Additional file 1: Fig. S4B**).

As an example of the complexities in data mining of such an intricate gene space for future reference, we offer an example using two well-known genes involved in sucrose and lignin biosynthesis.

Gene family analysis of SuSy and PAL shows differences in their regulatory regions in SP80-3280 and *S. spontaneum*

Sucrose Synthases (SuSy) catalyze the reversible breakdown of sucrose into UDP-glucose and fructose in carbon partitioning [33]. In agreement with previous work on sugarcane progenitors [34] (*S. officinarum*, *S. robustum* and *S. spontaneum*), 43 ScSuSy (Sugarcane Sucrose Synthase) CDSs identified in the SP80-3280 assembly branch out in phylogenetic inferences as five SuSy genes (hereafter ScSuSy1-5) organized in three groups: I (ScSuSy1 and 2), II (ScSuSy3 and 5) and III (ScSuSy4) (**Fig. 4A)**. Sorghum shares these 5 SuSy genes, indicating that they evolved before the sugarcane/sorghum divergence. RNA-Seq data from leaves and internodes of SP80-3280 (Ion PGM Sequencing) [28] shows expression of 34 of the 40 ScSuSy members,

suggesting ScSuSy1-2 (group I) and ScSuSy5 might control carbon flux from source to biomass conversion in stems, as they show higher expression in internodes than in leaves (**Fig. 4C**).

Different members of the SuSy gene family may have different functional roles and in sugarcane this was observed as different expression levels related to different TFBs identified. We identified five different top-ranked TFBs (with the highest score) in the ScSuSy1-5 members. Three of them are related to auxin and abscisic-acid hormone signaling (ScSuSy1, 3, 5). For ScSuSy1 genes, the TFBS analysis predicted the motif wATATATATw (MA1184.1) that is associated with RVE1, a morning-phased transcription factor integrating the circadian clock and auxin pathway genes that bind to the evening element (EE) of promoters [35]. For ScSuSy2 genes, we found the motif GACrAATryA (MA1374.1) that is associated with IDD which regulates photoperiodic flowering by modulating sugar transport and metabolism [36]. For ScSuSy3 genes, we found the AyACTAGTrT (MA0930.1) motif in 64% of its SP80-3280 copies and in all copies in the *S. spontaneum* and R570 monoploid genomes. It is associated with ABA-responsive elements (ABRE) that regulate stress response via ABA signaling. For ScSuSy4 genes, we found the TAGyAynTTT (MA1012.1) motif that is probably involved in regulation of the photoperiod and vernalization pathways. Finally, for ScSuSy5 genes, we found a CTGCTAGCAG (MA0564.1) conserved motif exclusively for ScSuSy5 genes in SP80-3280. This motif allows binding with an element associated with ABI3, which participates in abscisic acid (ABA)-regulated gene expression. Previous studies from our group had already pointed out ABA- and sucrose-induced genes associated with higher sucrose content in sugarcane [37].

SuSy produces the substrate for cellulose biosynthesis (UDP-glucose) and is commonly associated with cell wall and cellulose synthesis [38,39]. In view of the myriad of possibilities to convert lignocellulosic compounds into chemicals and fuels, defining phenylpropanoid biosynthesis pathway members in sugarcane is of great interest. Phenylalanine ammonia-lyase (PAL) is the first enzyme in phenylpropanoid biosynthesis [40–42] and silencing its expression has been associated to a reduction in lignin content [40–43]. Lignin is a major component of plant cell walls [18], and is responsive to the ethylene-releasing ripener (ethephon) in both leaf and internode [44].

Mapping of predicted proteins from SP80-3280 against the SUCEST-FUN Cell Wall Catalogue [43] (731 transcripts of 20 protein categories) identified 3,054 similar proteins (**Additional file 2:Table S5)**, including 47 PAL copies. Based on a Maximum Likelihood gene tree that includes sorghum, *S. spontaneum* and mosaic

monoploid R570 PAL sequences reveals five clusters (**Fig. 4B**), each containing at least one representative with a sorghum ortholog. *S. spontaneum* has 33 putative PAL genes, somewhat more than expected considering that the sequenced genotype is a tetraploid. The higher number may be due to expansion of PAL members in group I that occurred also for sorghum and the sugarcane hybrid genomes of R570 and SP80-3280. Group V has a higher number of SP80-3280 PAL members and all except one (ID 37780.4) showed expression evidence (**Fig. 4D**).

Regarding TFBS prediction within PAL regulatory sequences, we identified four different top-ranked TFBS. For PAL I, it was predicted an ArCAyATnTG (MA0930.1) element, which is associated with ABF3, a transcription factor involved in ABA and stress responses and acting as a positive component of glucose signal transduction. For PAL III, we found the element GGTCsGGCkC (MA0992.1), an element associated with AP2/ERF, a transcription factor involved in the regulation of gene expression by stress factors and by components of stress signal transduction pathways. For PAL Va, we found the element TCTAAAGTTT (MA0064.1), which is associated with PBF, a transcription factor involved in ABA, stress response and components of stress signal transduction pathways. Finally, for PAL Vb, we found the motif GCCGGAACGG (MA1009.1). This element is associated with ARF3, a transcription factor involved in auxin and ABA-regulated gene expression. In summary, our results corroborates reported findings [37] which reveal that PAL genes were induced by ABA.

In addition to PAL members expansion in group I, the CCR (Cinnamoyl-CoA reductase), COMT (Caffeic acid 3-O-methyltransferase) and 4CL (4-coumarate-CoA ligase) gene families, also related to phenylpropanoid biosynthesis, have much higher numbers of genes (620, 453 and 375, respectively) in sugarcane than sorghum [45] (44, 41 and 15, respectively). This is another challenge and opportunity for future functional characterization (**Additional file 2: Table S6)**.

The sheer number of sugarcane genes found so far, the large size of multi-gene families and the evidence that not all homo(eo)logs are expressed point to a very complex role of regulation in the determination of phenotypic differences. Consistent with the gene copy-richness of sugarcane, we inferred 15,737 transcription factors (TFs) from 57 families (**Additional file 2: Table S7)**, versus ~2,000 previously estimated [46]. The classification of core promoters and identification of Transcription Factor Binding Sites (TFBSs) in proximal

promoters was performed *in silico* and the percentage of core promoter regions with a TATA-box element was 47.72% and 12.76% for SuSy and PAL genes, respectively.

The TFBS identification pointed to a wealth of regulatory elements differentially distributed among members of the same gene family, i.e. SuSy and PAL (**Fig. 4C and D and Additional file 2: Table S8)**. In addition, using gene expression data of SP80-3280 plants grown in field conditions for 13 months, we have found evidence of a co-expression module, enriched for phenylpropanoid and lignin biosynthesis gene ontology terms (**Additional file 1: Fig. S5A**). This module comprises 116 transcripts, including one PAL (**Additional file 1: Fig. S5B**), whose expression is higher in internodes 5 and 9, than in leaves and immature internode (**Additional file 1: Fig. S5C**). It was possible to identify the TFBSs, predicted as putative regulators of the PAL gene family (**Fig. 4D**) within the upstream region of these co-expressed genes, suggesting that ABF, ERF, ZF-HD/C2H2, and ARF3 (**Additional file 1: Fig. S5D**) may also regulate other genes involved in lignin biosynthesis and metabolism. The most significant motifs found for each gene family (SuSy and PAL) were mapped to the promoter region of the remaining sequences from both SP80-3280 and R570 hybrids and *S. spontaneum* (**Additional file 2: Table S8 and Table S9**). Interestingly, only ScSuSy2 and ScSuSy3 motifs mapped in all species, suggesting that SP80-3280 hold particular regulatory elements involved in sucrose synthesis. Conversely, SP80-3280 and *S. spontaneum* share all predicted motifs for PAL genes (**Additional file 2: Table S9)**, suggesting that this gene family may be derived from the *S. spontaneum* ancestor.

Transposable element insertions may affect SuSy and PAL expression

Fewer transposable elements (TE) were identified in SP80-3280 gene space than in the AP85-441 *S. spontaneum* and mosaic monoploid R570 assembly, probably due to repetitive regions collapsing in the assembly even with the use of long synthetic-read sequencing (**Additional file 1: Fig. S6**, **Additional file 2: Table S10)**. All previously described TE families are represented in the three genome assemblies, disclosing few cultivar specific amplifications. The two modern cultivars (SP80-3280 and R570) have fewer TE counts than the *S. spontaneum* progenitor in normalized monoploid genomes. LTR retrotransposons are large contributors to genome composition at the chromosome assembly level. However, scMaximus (Copia) and scDel (Gypsy) LTR-retrotransposon families are similarly represented in both gene space and chromosome assemblies supporting their presence in transcriptionally active regions [47]. We also note that scCACTA

transposons are more represented at the gene space assembly than schAT while the scMutator family is similarly represented in both.

Functionally important TE insertions were identified in the ScSuSy gene family (**Fig. 4**). ScSuSy2 copies have a contrasting pattern, most *S. spontaneum* having TE insertions while most SP80-3280 homo(eo)logs do not – although SP80-3280 and *S. spontaneum* share one ancient insertion of schAT159 at similar distances from the ATG. ScSuSy3 genes are polymorphic between species and within SP80-3280, with 6 copies having no TE and 5 in which different TEs may impact expression. In particular, scga7_uti_cns_0020964:7575-17575 (-) harbors a full LTR at 280 bases from the ATG. Most ScSuSy4 copies have no TE insertion but interestingly, as described for ScSuSy2, SP80-3280 (scga7_uti_cns_0226458:7638-16073 (-)) and *S. spontaneum* (Chr1B:33406669-33416669 (-)) share one ancient schAT159 insertion. Finally, ScSuSy1 has similar patterns of TE presence and absence in both genomes, and ScSuSy5 genes have no insertions in the promoter regions of either *S. spontaneum* or SP80-3280. Furthermore, PAL genes from group I exhibit most of the copy variation and harbor TEs inserted near the promoter region. Only two copies from SP80-3280 and *S. spontaneum* lack TE insertion in PALs from group I.

Sugarcane and sorghum polymorphisms support recent allotetraploidy and suggest candidate genes for morphological and physiological differences between these taxa

Despite a common foundation for evolving high sugar content with similar SuSy genes (ScSuSy1-5), sugarcane and closely related sorghum have taken different paths since sharing ancestry. We identified 10,586 natural SNP variations (SNVs) between sorghum and sugarcane 4,140 unique genes, mostly bi-allelic (80.8%), but 6.2% tri-allelic and 0.97% tetra-allelic (**Fig. 5**). The overwhelming predominance of biallelic variations indicates that many sorghum genes are represented by two discernible sugarcane copies, supporting the theory of allotetraploidization shortly after divergence with sorghum ca. 3.8~4.6 MYA [48], creating two sugarcane 'subgenomes'. Recently published results from Vieira et al. [49], demonstrate that sugarcane meiotic chromosomes behave as bivalents, supporting this inference. Autotetraploidization after *Saccharum* speciation ca. 3.1~3.8 MYA may have further contributed to allelic richness within each sugarcane 'subgenome'. The preservation of as many as four functionally different alleles at a locus, with cases observed on all except one chromosome (Chr 10 **- Fig. 5**), is consistent with the well-known heterozygosity of sugarcane cultivars and

associated susceptibility to inbreeding depression. However, genes for which sugarcane has only one allele are more abundant than 3- or 4-allele, perhaps reflecting cases in which a single gene copy is sufficient, or in which occasional exchanges between subgenomes have homogenized multiple homo(eo)logs.

Further, 1,334 SNVs that differentiate sugarcane from sorghum in 585 single-copy genes in diploid grasses include frameshifts, premature termination, erroneous splicing, loss of stop codons and incorrect translation initiation (**Additional file 1: Fig. S7**, **Additional file 2: Table S11)** in genes significantly enriched in transcription, DNA-dependent cell organization and biogenesis in the nucleus and endoplasmic reticulum (**Additional file 2: Table S12)** comprise a rich slate of candidates for causes of morphological and physiological differences between these taxa.

The gene space contribution towards a chromosome level assembly of a sugarcane commercial hybrid

Notwithstanding the fragmented nature of our assembly, we explored how it could contribute beyond the gene space toward a whole genome assembly of the hybrid sugarcane genome. Previous analysis of grass genomes revealed extensive conservation of gene order overlaid with a background of small-scale chromosomal rearrangements and numerous localized gene deletions, insertions and duplications [50]. Recently published estimates of the levels of gene synteny between *Sorghum bicolor* and the sugarcane cultivar R570 found that 83% of the genes are arranged co-linearly in the two genomes [13]. In our assembly of SP80-3280, 79,094 (17.6%) contigs had at least two predicted genes and could therefore be used to compare the order of genes in SP80-3280 to those of sorghum. To avoid the need to resolve multiple comparisons to duplicated regions in the sorghum genome, we generated a sequence similarity-based clustering of all coding sequences from both genomes and used the genes in clusters with only one sorghum gene as anchors to evaluate synteny (**Additional file 1: Fig. S8**). We found that 9,319 (2.1%) SP80-3280 contigs had at least two synteny anchors and 85% (7,906 – 1.8% of all contigs) of these contigs were fully syntenic (**Additional file 1: Fig. S9A, B**), *i.e.* had all genes in the same order and orientation in SP80-3280 contigs and the sorghum chromosomes (**Additional file 2: Table S13)**. To evaluate the effect of SP80-3280 assembly fragmentation on the number of segments with conserved gene order ("syntenic blocks") per contig, we used a Monte Carlo method to simulate the fragmentation of the chromosomes and contigs of the *Saccharum* R570 and *S. spontaneum* genomes. We performed 1,000 rounds of simulation for each genome and, at each round, sampled 10,000 random fragments from each of these two genomes, while simultaneously sampling the same number

of contigs from SP80-3280's assembly. Sampled contigs and contig fragments were constrained to follow the distribution of the number of genes per contig observed for the full SP80-3280 assembly. The number of syntenic blocks on each fragment was then evaluated and the relative frequency of contigs/fragments per number of syntenic blocks is shown in additional file 1, **Fig. S10C**. We observed that contigs and fragments harboring a single syntenic block are sampled at similar frequencies in all genomes analyzed. While an increase in sequencing coverage would lead to improved estimates of co-linearity, our analysis of the small subset of contigs with two or more marker genes suggests that levels of genomic rearrangement in SP80-3280 are similar to those expected anywhere in the genomes of the other two *Saccharum* species.

Finally, to allocate the gene space into potential physical groupings we aligned the SP80-3280 transposable element (TE) masked BWA-SW to chromosome level assemblies of the *S. spontaneum* tetraploid AP85-441 genome [14] and the R570 [13] monoploid genome data. Multiple correspondence analysis (MCA) with hierarchical clustering of the sequences enabled us to allocate the gene space contigs into 6 clusters, an important contribution to future scaffolding efforts. From the total of 450,609 contig sequences, 418,471 (92,86%) produced a BWA-SW alignment against the *S. spontaneum* [14] and R570 [13] assemblies (**Fig. 6A**) and protein alignment among these three species are consistent with MCA results (**Fig. 6B and C**). Contigs were also mapped against a collection of 778 targeted sequenced BACs of which 347 are from SP80-3280 and 431 from R570. All BACs had a corresponding contig match against the assembly. This collection shows centromeric regions and non-TE multigene families are the most covered (64x). An R gene locus (I2C-2) found in cluster 3 of SP80-3280 and in chromosome 9 of R570, was verified for co-location with a Ca^{+}-dependent kinase, a *dog1* (delay of germination 1) and an aminotransferase. The co-location was confirmed in R570 and SP80-3280 BACs showing up to eight copies of each gene (**Additional file 1: Fig. S10**).

DISCUSSION

This assembly presents 373,869 genes. The gene space described here represents a significant step in understanding the haplotype origin of the hybrid genome. Approximately 12.25% of the SP80-3280 genome sequence is of *S. spontaneum* origin [14], supporting previous studies [10,11]. The comparison against different sets of genes (sorghum, CEGMA, BUSCO, mitochondrial and chloroplast) shows that the gene space assembly contains the majority of the genes queried in at least one copy. The total of predicted genes (373,869)

is around 10x, 14x and 13x higher than those for monoploid genome assemblies of *S. spontaneum* [14], sugarcane R570 [13] and sorghum [52], respectively. We also detected that single-copy genes in diploid grasses are present in 2-6 and up to 15 copies. These findings agree with the predicted 8 to 14 copies for *S. spontaneum*, depending on the cytotypes, and for modern sugarcane varieties [53]. The total number of predicted genes, the high quality of alignments and the detection of more than one copy for single-copy genes in diploid grasses indicates that the assembly provides homo(eo)logous resolution for a large fraction of the gene space (~87%).

Although for sugarcane modern varieties we expect eight or more copies of each chromosome, it is possible that each homolog does not contains a copy of every gene, because of potential gene loss. In addition, it is also possible that some homeologs were not identified in our assembly because of assembly or sequencing difficulties in regions with highly repetitive sequences. Single-copy genes from diploid grasses correspond to mostly 2-6 copies (up to 15) of sugarcane genes in our SP80-3280 assembly and nucleotide differences are present mainly in the upstream regulatory region. This highlights the importance and complexity of studying homo(eo)logs expression in sugarcane and adds great value to the development of molecular markers for breeding in gene promoter regions. The differences in gene upstream sequences may potentially affect the expression level among the copies and across the studied tissues. This was also reported for the polyploids cotton [54] and wheat [55]. Expression differences among homo(eo)logs in polyploid species may play a crucial role in increasing adaptability to environmental stresses (such as salinity [56], heat and drought [57]) and in improving performance of new cultivars. These differences highlight the importance of our assembly which discriminates homo(eo)logs for most genes, for example providing important information for the selection of target sequences (genes or promoters) to produce transgenic sugarcane plants. With the homo(eo)logs identified, one could discard a sequence that is not expressed or use genome editing tools to modify a target sequence to increase its expression. It is also possible to identify the progenitor contributing a homo(eo)log (e.g., *S. spontaneum*, *S. officinarum* or a parent in a cross) and select the homo(eo)log from the progenitor that has the phenotype of interest.

In an attempt to organize the contigs, we allocate them in 6 clusters using MCA with hierarchical clustering of the sequences. The majority of proteins predicted from chromosomes 1, 2, 3 and 4 (in both *S. spontaneum* and R570) have their best matches located in SP80-3280 contigs from clusters 2, 5, 6 and 1,

respectively (**Fig. 6B** and **C**). On the other hand, clusters 3 and 4, which contain contigs matching to multiple chromosomes, including those in which chromosomal rearrangement events were demonstrated in comparison to sorghum: SsChr5, SsChr6 and SsChr7 from *S. spontaneum* [14] and six R570 hom(oe)ology groups HG5-HG10 [13].

Assembling the genome of a polyploid interspecific hybrid is of especially high value for breeders. The assembly, gene prediction, and annotation provided can bridge long standing gaps of knowledge allowing them a more efficient use of genomic tools. Sugarcane's large autopolyploid genome, predominant clonal propagation, and need for extensive phenotyping to determine breeding values, have contributed to the relatively slow (~1% per year at most) rate of progress in improvement of sugarcane [58] and perhaps other autopolyploids. The demonstration that most of its many homo(eo)logs are expressed, often with tissue-specificity, and that transcription factor binding sites and TE insertions differ among homo(eo)logs, suggests complex constraints that may necessitate unusual richness of information to make effective decisions about selecting some homo(eo)logous alleles at the expense of others in autopolyploid breeding populations. These principles may apply widely to many plants with large polyploid genomes that include many of those most efficient at converting solar radiation to biomass.

The present work discloses a large collection of gene space homo(eo)logs diversity, taking advantage of novel sequencing technologies, adding over 3Gb of sequence not previously reported, in addition to genome annotation, data mined homo(eo)logs, and explored regulatory regions of SuSy and PAL. The presented gene space of the sugarcane genome is a fundamental step towards a high-quality chromosome resolved assembly from a current commercial hybrid. The genome sequence released for this interspecific polyploid supports its recent allotetraploid nature, reveals differences in promoter regions associated to a diverse gene expression pattern and transposable elements contributing to fine tuning of the sugarcane genome.

METHODS

Plant material

Leaves from SP80-3280 were collected and frozen in liquid nitrogen. Genomic DNA was extracted using DNeasy Plant Mini Kit (Qiagen) following the standard protocol. DNA integrity was analyzed using the

Agilent High Sensitivity DNA Analysis Kit (Agilent Technologies) and Agilent 2100 Bioanalyzer Instrument. Quantification was done using Quant-it^{TM} PicoGreen® dsDNA Assay Kit (ThermoFisher Scientific) and SpectraMax M2 microplate reader (Molecular Devices).

Sequencing Illumina Long-reads and Assembly

We used Illumina Synthetic Long-read sequencing technology, which provides very accurate long reads with a mean read length of roughly 5 kb, thus being able to represent polymorphisms across all copies of chromosomes. Genomic DNA was sheared into 5-10 kb fragments and diluted in a 384-well plate. DNA fragments were ligated with PCR primers and specific sequences, which identify the 5' and 3' ends. The fragments from each well were amplified, fragmented and barcoded with unique indices, to create a TruSeq Synthetic Long-Read DNA library. In total, 26 libraries were made. The short fragments created in the second step of fragmentation were pooled and sequenced on the HiSeq instrument at the Illumina Service Genome Network. The reads from each of the 384 wells were pre-processed to correct sequencing and PCR errors. Contigs were produced from the paired-end information and further scaffolded together to resolve repeats and fill in gaps. In this step, the software removes fragments containing inconsistent bases at a higher rate than expected from sequencing error rate. More details on the informatics pipeline for short read scaffolding into long reads are available in the Fast Track Services Long Reads Pipeline User Guide [59].

To assemble sequences we used a two step approach: *i)* the Celera Assembler [60] (CA) was used for overlap computation and layout building; *ii)*the *tig-sense* module of the HBAR-DTK (Hierarchical-Based AssembleR Development ToolKit) from Pacific Biosciences [61] was used to construct consensus sequences. This was motivated by the fact that the CA, which uses the overlap-layout-consensus method, is more robust than *de Bruijn* graph approaches. However, some adjustments needed to be made. CA, designed for Sanger reads, only accepts quality scores between 0 and 40. Since synthetic long reads are very accurate and some of the base qualities exceeded this upper bound, we set the quality scores over Q40 as Q40 to allow them to be appropriately parsed. The consensus module was also adapted for the analysis of big complex genomes. The substantial number of contigs generated initially (roughly 450,000, half of them singletons) resulted in several files in a folder that hindered I/O operations. So, we *i)* modified *tig-sense* to automatically create subdirectories that contained not more than a thousand contig FASTA files, reducing delays for file lookup; *ii)* divided contig

processing into non-singletons and singletons, prioritizing non-singleton contigs; and *iii)* created a work history so that the program could be resumed after a halt. Overall, these modifications allowed us to reduce the running time of the consensus pipeline by one or two orders of magnitude. In order to identify problematic regions, after the assembly step, we have assessed the assembled contigs using a read coverage analysis by mapping reads back to contigs. After sorting contigs from highest coverage to lowest, we found that only 0.1 Gbp of contigs had very high coverage (**Additional file 1: Fig. S11**).

Sequencing BAC clones and assembly

A total of 780 independent BACs were sequenced using Roche454 sequencing technology. Each BAC clone was tagged with a unique barcode and sets of 12 BACs were pooled in one gasket. We assembled BACs individually as described [62] and obtained a total of 49.6 Mbp of assembled sequence, with a mean length of 107 Kbp. The BAC data includes 317 R570 BACs [62], 116 additional R570 BACs and 347 from SP80-3280.

Assembly Validation

Comparison with Sugarcane BACs

Assembled contigs were aligned against a set of 780 BACs with BWA mem (BWA, RRID:SCR_010910), using default parameters. Alignment data was processed for coverage with the aid of SAMTOOLS (SAMTOOLS, RRID:SCR_002105) v1.1 and BEDTools (BEDTools, RRID:SCR_006646) v2.25 and selected matches were at least 10 kbp long and covered 90% or more of the contig. Additionally, the unassembled synthetic long reads were aligned to the same set of BACs, to check for discrepancies among contigs and long reads, which could be indicative of regions that were not assembled.

Comparison with Sorghum CDS

The set of 39,207 annotated sorghum coding sequences (CDS), release version v2.1, were downloaded from Phytozome [63]. These were aligned against the assembled contigs with BLASTn (v2.2.30+) using default parameters. For each sorghum CDS, we identified the longest fraction of the coding sequence contained within a single unitig. Only hits with at least 80% identity at the nucleotide level were considered for computing coverage. For any CDS with multiple HSPs (High-scoring Segment Pair) against the same contig that passed

the filtering criteria, we used the union of such hits, excluding any potential overlap. Given that most contigs contained only one or two genes, we expect very little influence of spurious hits to different gene regions.

Comparison with CEGMA

A total of 248 Ultra-conservative core eukaryotic genes classified by Korf Lab [22] were assessed in our sugarcane assembly with '-g' and other default options of CEGMA (CEGMA, RRID:SCR_015055) v2.5. To assess the presence of putative homo(eo)logs for CEGMA regions identified on the assembly, the sequences were retrieved according to the coordinates provided on CEGMA output. Sequences were aligned back to the genome using BLASTn with default parameters. Matches with identity and query coverage greater than 90% were considered for calculation of alignment frequency.

Comparison with BUSCO

The assembly was assessed for the presence of the 1,440 core genes from the Plantae lineage of Benchmarking Universal Single-Copy Orthologs (BUSCO, RRID:SCR_015008) [23]. BUSCO performs gene prediction and orthogonality assessment using Augustus (Augustus: Gene Prediction, RRID:SCR_008417) [64] and HMMER3 (HMMER, RRID:SCR_005305) [65]. Since these steps demand huge resources, we partitioned sugarcane contigs (4.3Gbp) into six groups with similar length and processed BUSCO in parallel. After we merged results, we applied orthogonality assessment algorithm once again as thresholds that BUSCO exploits to discern actual single-copy orthologs from paralogs.

Comparison of the mitochondrial and chloroplast genomes

To reconstruct the SP80-3280 mitochondrial and chloroplast genomes, we have used as reference the complete genomes of *Saccharum* hybrid chloroplast (NC_005878.2) [24] and the *Saccharum officinarum* mitochondrial chromosome 1 (LC107874.1) and chromosome 2 (LC107875.1) [25], downloaded from NCBI. The SP80-3280 genome contigs were aligned using BLASTn against their respective references and the best hits were selected based on cutoff E-value $\leq 1x10^{-15}$, with contig coverage ≥ 90% and identity ≥ 70%. The BLASTn alignment results identified 2,482 and 909 contigs for the two mitochondrial chromosomes, respectively; and 51,768 contigs for the chloroplast genome. To reconstruct the consensus sequences and do the genome

annotation we have used the CLC Genomics Workbench tools (CLC Genomics Workbench, RRID:SCR_011853) [66]. The contigs used for genomes reconstruction presented mean size of 4Kb, with coverage depth higher than 20x.

Using the CLC Tools and the Genome Finishing Module, the selected contigs were aligned to their respective references and consensus sequences extracted, filling the gaps with N's. The reconstructed consensus sequence aligned against the chloroplast genome presented 99.99% and 99.99% of coverage and identity respectively, and there were identified only 6 mismatches and 2 gaps, most of them located in intergenic regions and in one of the rRNA23S copies with protein frame preservation.

The alignment against mitochondrial chromosomes 1 and 2 presented 99.85% and 99.93% of coverage and 99.90% and 99.94% of identity, respectively. The consensus sequences were annotated using their respective NCBI references with the CLC tool "Annotate from Reference", where all genes, tRNAs, rRNAs and miscellaneous features were totally transferred. For the mitochondrial chromosome 1, 237 mismatches and 63 gaps were identified, most of them present in intergenic regions and only 2 mismatches in 2 rRNA genes, with proteins frame preservation. And for chromosome 2, we identified a region composed by 19 N's inside a repetitive AT's region. In addition, the reconstructed chromosome has 57 mismatches and 16 gaps, all of them present in intergenic regions.

Comparison with Sugarcane ESTs

A set of 134,840 ESTs from leaves, internodes and roots samples exclusively from SP80-3280 [20] were aligned to the contigs sequences using SPALN v 2.3.3 [67] applying mapping and alignment algorithm (-Q 5) and admitting all possible matches for each sequence (-M 1000). Coordinates of aligned ESTs were compared to gene annotation using Bedtools intersect utility [68]. Alignments might be explored through a GBrowse environment available at http://sucest-fun.org/cgi-bin/cane_regnet/gbrowse2/gbrowse/microsoft_genome_moleculo_scga7/).

Genome Annotation

Gene prediction

Contigs were annotated using a pipeline developed in house, previously used for BAC annotation. Transposable element (TE) discovery and masking was done using LTR harvest, LTR digest, CrossMatch against *Utricularia gibba* TE DB and RepeatMasking [69] of Viridiplantae [70] and previously known sugarcane TEs [47].

Genes were discovered and annotated using masked contig sequences. *De novo* predictions were done with Augustus [64], Glimmer HMM (GlimmerHMM, RRID:SCR_002654) [71], GeneMark HMM [72], SNAP (SNAP, RRID:SCR_007936) and PASA (PASA, RRID:SCR_014656) [73] with rice models and sugarcane EST and RNA-Seq data [28]. Alignments were also generated against reference protein DBs (sorghum, known sugarcane and Phytozome) using Exonerate [74] and BLAST [75] (v2.2.30+). Both *de novo* and alignment evidence were used for consensus annotation with EVidenceModeler (EVidenceModeler, RRID:SCR_014659) [76] with greater weight given to experimental and alignment information. Functional assignment was derived from protein DB best hits and InterProScan 5 (InterProScan, RRID:SCR_005829) [77] results.

GeneOntology annotation

For functional annotation of predicted proteins from SP80-3280, all sequences were aligned to UniRef50 clusters, a dataset of representative sequences clustering high similarity proteins from UniProtKB [29], using BLASTp (v2.2.30+, *-evalue* $1x10^{-5}$). Sequences that fail to align in this first approach were also searched against the RefSeq non-redundant protein database. Gene Ontology mapping and annotation of sequences with positive BLAST results was performed using Blast2Go (Blast2GO, RRID:SCR_005828) framework [78].

Reference-guided RNA-Seq Assembly

We used Trinity (Trinity, RRID:SCR_013048) version 2.0.6 for reassembly of the Sugarcane ORFeome [28] using the genome as a reference, with a minimum contig length of 250 bp (genome_guided_max_intron 3,000, genome_guided_min_coverage 5, genome_guided_min_reads_per_partition 10) to identify transcript models. SP80-3280 RNA-Seq reads from 3 tissues (leaves and immature and intermediate internodes) were used for alignment against the reference genome and partitioned into read clusters, which were then individually assembled using Trinity genome-guided methods. Trinity and genome-guided methods used a fixed k-mer size

of 25nt. In this new assembly, 269,050 genes and 275,807 transcripts were recovered. The quantity of transcripts recovered by the reference guided-assembly was higher, and thus closer to the number of predicted genes (374,774), than the *de novo* assembly. Transcript expression level was estimated by FPKM (fragments per kilobase of exon model per million reads mapped).

Identification of Putative Homo(eo)logs and Count Estimation

We downloaded the *Sorghum bicolor* genome assembly v2.1 from Phytozome and took 2,051 single-copy genes according to Han *et al*. [79], which were also present as single copies in the genomes of *Oryza sativa* and *Brachypodium distachyon*. We aligned the coding sequences of these sorghum genes to the coding sequences of predicted sugarcane genes from the SP80-3280 assembly, using the BLASTn (v2.2.30+, *-evalue 1x10*$^{-6}$). We filtered alignments with at least 80% nucleotide identity, based on Wang *et al*. [50], covering at least 70% of both the sugarcane and sorghum sequences. Sugarcane gene models aligned to the same single-copy sorghum gene were denoted as putative homo(eo)logs. Finally, we counted the number of copies for each gene.

We clustered all putative homo(eo)logs based on each single-copy sorghum gene to get estimates of sequence differentiation. We aligned the coding sequences for each pairwise combination in each gene cluster, using BLAT (BLAT, RRID:SCR_011919) v35 [80] (*–minIdentity=0 –minScore=60*). One of the clusters had 21 putative homo(eo)logs, which is higher than the number of chromosome copies expected for sugarcane and was discarded from the analysis. Next, we parsed the alignments to obtain estimates of copy differentiation considering both SNPs and INDELs. We gathered distance estimates from all pairs, from all clusters, to obtain dissimilarity distributions.

Putative Homo(eo)logs characterization

Upstream region analysis

We also assessed the dissimilarity levels of regions upstream (potential promoter regions) of the predicted sugarcane putative homo(eo)logs. We initially collected three different sequence ranges (100 bp, 500 bp and 1,000 bp) upstream of the predicted gene start site. Next, we aligned these upstream sequences for each pairwise combination in each cluster, again using BLAT v35 [80] (*–minIdentity=0 –minScore=30*). Finally,

for each distance range, we parsed the alignments and computed the dissimilarity level considering both mismatches and gaps to obtain a distance matrix for the upstream region of each cluster. To avoid partial alignments of the upstream sequences, only alignments up to 20% shorter or longer than the expected sequence length were considered. Note that the dimension of the distance matrix varied between gene clusters, according to the distribution of cluster sizes shown in **Fig. 2A**.

Insertions and Deletions between gene copy Coding Sequences

To investigate the occurrence of frameshift mutations between putative homo(eo)logs, we built multiple alignments of its coding sequences for each cluster, with MUSCLE (MUSCLE, RRID:SCR_011812) v3.8.31 [82], using default parameters. We then computed the length distribution of insertions and deletions in the coding sequences, to differentiate between frame-preserving and frameshift indels. We parsed the CDS alignment for each pairwise combination of putative homo(eo)logs and counted the number of occurrences of gaps of a given length. We then pooled counts from all copy combinations to get a joint estimated distribution.

Tissue-Specific Homo(eo)logs Expression Analysis

We used RNA-Seq data [28] from leaves (*L*), immature (*I1*) and intermediate (*I5*) internodes of SP80-3280 to find the expression of putative tissue-specific putative homo(eo)logs. These reads were initially aligned to the sugarcane genome assembly using TopHat2 (TopHat, RRID:SCR_013035) [83] version 2.0.9 (*library-type fr-firststrand*). We allowed reads to be aligned to up to 20 contigs of the genome assembly to identify alignments to different homo(eo)logs (*--max-multihits 20*) and supplied TopHat2 with the putative homo(eo)logs' annotation as a GTF file (*--GTF CDSMapping-homo(eo)logs.gtf*), in order to direct TopHat2 to align the reads to this transcriptome first.

Besides the *TopHat2* alignment, we used the RSEM (RSEM, RRID:SCR_013027) tool rsem-calculate-expression (version 1.2.31) to quantify the expression of predicted genes (bowtie2, fragment-length-mean, fragment-length-sd and calc-ci parameters). An in-house Perl script was used to estimate the mean length and standard deviation for each RNA-Seq library. The main output of *Tophat2 BAM* formatted file [84] *accepted_hits.bam* was used with *RSEM* to estimate the transcriptome expression profile. We developed in-house Perl and R language (version 3.3.2) scripts to find the number of putative expressed homo(eo)logs for

each single-copy genes in diploid grasses, using the information from *genome annotation* file (GFF format), showing the gene structure, the transcriptome annotation and respective TPM (Transcript Per Million) abundance. The previous information allowed the creation of the homo(eo)logs GFF file. We also applied TopHat2 to find the number of putative homo(eo)logs expressed only in *antisense* orientation, using the same protocol described above, and the *antisense* reads of RNA-Seq previously identified by Nishiyama *et al*. [28].

ScSuSy and ScPAL gene family analysis

We used the sugarcane and sorghum SuSy protein sequences reported by Zhang et al. [34] as query for a BLASTx (v2.2.30+) search in the predicted proteins from SP80-3280, *S. spontaneum*[46] and R570 genome assemblies [13]. Putative SuSy genes were then filtered by query coverage >=80% of at least one of the five ScSuSy from Zhang et al. [34] and by PFAM [85] domain search, considering only those containing both the conserved sucrose synthase and glucosyl-transferase 1 domains.

Based on BLAST and keyword search ('Phenylalanine ammonia-lyase', 'PAL' and 'EC:4.3.1.24') in two databases (Plant GDB, http://www.plantgdb.org/ and Phytozome [63]) we found 8 different PAL genes in the sorghum genome, the same number previously reported [86]. For sugarcane, PAL genes were retrieved from an EST Cell Wall catalogue [43], which was used as query together with sorghum PAL genes for a BLASTx (v.2.2.30+) search to identify PAL genes in the predicted proteins from *S. spontaneum* [51] and R570 genome assemblies [13]. Putative PAL genes were then filtered by query coverage ≥ 80% of the sorghum PAL genes and by PFAM [85] domain search, considering only those containing the Aromatic amino acid lyase domain. Also, sequences not containing the PAL conserved amino acid motif Ala-Ser-Gly [87,88] and an essential Tyr110 [89] were excluded.

For both SuSy and PAL, nucleotide sequences (CDS) were aligned with clustalw [90] software in MEGA (MEGA Software, RRID:SCR_000667) 7.0 [91] and maximum likelihood trees were constructed with 1,000 bootstraps and Gaps/missing data treatment "*use all sites*". Expression heatmap was constructed using log2 transcript per million (TPM) from previous RNA-Seq data [28].

Cell wall-related genes

For the identification of cell wall-related genes in the sugarcane genome we used the Sugarcane SAS Cell Wall catalogue [43] as a reference. The search was carried out using tBLASTn (v2.2.30+, *-evalue* $1x10^{-6}$). These were manually re-annotated to produce a sugarcane cell wall catalogue with 3,054 sequences, classified in 10 cell wall categories.

Transcription Factor analysis

For the identification and classification of sugarcane predicted proteins into transcription factor (TF) families, we used the classification rules and tools described in GRASSIUS [46]. The search was carried out using HMMER v3.1b1 [92] and all significant HMM hits with *e*-value smaller than $1x10^{-3}$ were kept.

Promoter region analysis

Transcription Start Site (TSS) and promoter region classification

We evaluated promoter regions of genes associated with cell wall and sugar metabolism, ScPAL (Sugarcane Phenylalanine ammonia-lyase) and ScSuSy (Sugarcane Sucrose Synthase), respectively, as described above. A total of 47 ScPAL and 44 ScSuSy was used. To extract the candidate promoter region, we selected, when available, up to 1,500 nt upstream from the annotated start position of the gene, consisting of a core promoter (500 nt upstream of the start position) and proximal promoter (1,000 nt upstream of the core promoter). Next, we used TSSPlant [93] to predict the TSS of the genes and the type of promoter (TATA-box, TATA-less). The software was set to report high score, sense only TSSs.

Transcription Factor Binding Site (TFBS) in silico characterization

The annotation of TFBSs in the proximal promoter regions was performed in two steps: *de novo* prediction of TFBS motifs in smaller subsets of sequences and mapping the predicted TFBSs in the remaining promoter sequences. Sequences were partitioned in 10 subsets: five ScPAL groups and five ScSuSy groups. We then applied MEME (MEME Suite - Motif-based sequence analysis tools, RRID:SCR_001783) [94] and MotifSampler [95], with default parameters, to each of these datasets to determine putative TFBS motifs. Both were restricted to search for at most 6 motifs with 10nt or less. MEME candidates were a subset of MotifSampler's. MotifSampler ran for 100 cycles; following the manual we selected, from the 10 top-ranked

motifs, the first 5 that occurred at least 10 times in the different cycles. Each of the resulting 35 candidate motifs was searched in the JASPAR public database [96], with partial positive matches for all of them.

To evaluate the significance of the motifs we measured their frequency in promoter regions of each of the original gene families and compared them with the frequency of each of these motifs in the promoter regions of the other SP80-3280 predicted genes. We also mapped the motifs of each ScSuSy and ScPAL gene family respectively in the promoter region of the ScSuSy and ScPAL genes from *S. spontaneum* and R570. Candidate motifs were mapped with MotifLocator [95]. For characterizing background sequences, we trained a first order Markov chain [95] trained on SP80-3280 coding regions that were previously shuffled using the fasta-shuffle-letters tool [94]. The parameters were set to full match of the motif in the target sequence and score 95% above of the background.

Co-expression analysis

A field experiment was conducted at the Agricultural Sciences Center of the Federal University of São Carlos in Araras (22°21’25’’S and 47°23’3’’W) in the state of Sao Paulo, Brazil. Trial plots of SP-3280 consisted of four rows of 10 m long and spaced 1.35m apart. The field experiment was initiated in October 2012 and extended up until November 2013, representing the conditions under which “one-year” sugarcane crops are cultivated. Aiming to carry out observations throughout growth and development, tissue samples of the +1 leaves (L1) and upper (I1), immature (I5) and mature (I9) internodes were collected from two plots (two technical replicates) after 4, 8, 11 and 13 months of planting.

RNA was extracted for four biological replicates, two from each plot, using the TriZol method, treated with DNase I and purified. A pool of samples from leaves and a pool of internodes was used as a 'reference sample' for hybridization experiments on a customized 4 × 44 K oligoarray (Agilent Technologies) for sugarcane (CaneRegNet), conducted following the recommendations proposed by Lembke et al. [97]. The oligoarrays were read using the GenePix 4000B scanner device (Molecular Devices) and the fluorescence data was processed by Feature Extraction software 9.5.3 (Agilent Technologies).

Log2 transformed expression data was used for discovery and the analysis of co-expression modules, on CEMiTool R package [97]. The adjacency matrix was calculated by estimating the Spearman's correlation coefficient between all pair of genes and raised to a soft thresholding power (β) of 14. TopGO (topGO,

RRID:SCR_014798) R package [98] was used for gene ontology enrichment analysis for each module and node and edge files were generated for use with the Cytoscape (Cytoscape, RRID:SCR_003032) network visualization program [99].

SNP variants (SNVs) analysis compared to genic regions in *Sorghum bicolor*

The 450,609 sugarcane contigs (183,322 singletons and 267,287 unitigs) were aligned to the sorghum genome sequence [52] using the BWA MEM v0.7.10 [100] and contigs with mapping quality larger than 20 were used for variant calling. SNVs were called using samtools v1.1 and bcftools v1.1 [84]. Using in-house Python scripts, extracted SNVs were screened when sugarcane contigs were located on the genic regions of the sorghum genome and two or more sugarcane contigs were aligned to the same sorghum gene. Then, the number of SNVs in each gene was counted according to four-base changes.

SNVs that are homozygous in sugarcane were extracted for further analysis. SNVs mapping to coding regions, splicing sites, stop codons and transcription initiation sites were classified as potential large-effect SNVs.

Functional Enrichment Test

Arabidopsis GO-slim gene annotation was used for functional enrichment analysis. GO-slim terms were assigned to sugarcane genes based on sequence similarity inferred from best BLASTp (v2.2.30+) hit. We used a binomial distribution based on the proportion of a GO-slim term among all annotated genes in the sorghum genome as the null distribution. The binomial test was used to assess functional enrichment, with a significance threshold of $p > 0.05$.

Conserved Synteny Blocks

DNA sequences for all CDSs from *S. spontaneum* [51], R570 [13], *S. bicolor* [101] and SP80-3280 were aligned using the BLASTn program. Results from BLAST searches, with e-value <= 10^{-5}, were parsed using an in-house Python script to filter alignments covering at least 70% of the length of both the query and hit sequences. A second filter, requiring at least 80% identity was also applied and the resulting pairs of queries and hit sequences were classified into putative orthologous groups using the union-find algorithm. We selected putative orthologous groups present in all three organisms but with only one *Sorghum* gene to be used as

markers to detect blocks of conserved gene order (syntenic bocks) in comparisons of SP80-3280 and *S. spontaneum* against the genome of *S. bicolor*, thus avoiding the complications of a direct comparison of the two polyploid genomes (**Additional file 1: Fig. S8**). Another Python script was used to detect the syntenic blocks in both *Saccharum* genomes and to count the number of syntenic blocks in each contig. In order to evaluate the effect of genome fragmentation on our estimates of gene conservation, a Monte Carlo simulation of chromosome fragmentation was performed on the R570 and *S. spontaneum* genomes. We sampled 10,000 random regions of the R570 and *S. spontaneum* genomes, with fragment lengths constrained to follow the distribution of contig lengths observed for SP80-3280. We performed 1,000 rounds of these simulated fragmentations, every time allowing genomic fragments (and the genes within them) to be chosen randomly throughout the genome, with no bias to marker genes. We assessed the degree of conservation through the fraction of contigs with two or more marker genes that were found in the same order in the *Saccharum* genome fragments and in the *S. bicolor* genome.

Chromosome Synteny Multiple Correspondence Analysis with Clustering

We performed a multiple correspondence analysis (MCA) with clustering of the best local alignment hit of masked contigs. Input data were the 450,609 contigs of the sugarcane synthetic long read assembly and the masked genomic sequences of *S. spontaneum* [51] and R570 [13]. We used the masked sugarcane contig sequence produced by the annotation pipeline, excluding 69,879 sequences that were fully masked.

The contigs were aligned to the grass genomes using BWA-SW v0.7.12-r1044 [100]. We used an in-house Perl 5 script to retrieve the highest scoring hit for each contig and generate a table for input into R v3.2.1 [81]. This table contained the chromosome hit, if any, for each contig against each reference genome.

We then used the FactoMineR (FactoMineR, RRID:SCR_014602) R package v1.31.3 [102], along with the missMDA missing data handling auxiliary package v1.8.2 [103]. We performed MCA with these data, *i.e.*, chromosome hit number information for each contig was treated as a set of categorical variables and represented in the two principal component dimensions. This was followed by hierarchical clustering in these two dimensions, as well as figure rendering, using the Hierarchical Clustering on Principal Components (HCPC) function of FactoMineR.

In order to identify the correspondence between *S. spontaneum* and R570 chromosomes and SP80-3280 clusters, protein sequence alignment between the cultivar variety and the ancestor and R570 was performed with BLASTp considering an e-value threshold of $1x10^{-5}$. The best hit with a minimum query coverage of 90% was selected for visual representation of the alignment results with Circos plot.

ADDITIONAL FILES

Additional file 1.doc contains Supplemental Figures S1 to S11

Additional file 2.xls contains Supplemental Tables S1 to S13

DECLARATIONS

List of abbreviations

CEGMA: Core Eukaryotic Genes Mapping Approach

BUSCO: Benchmarking Universal Single-Copy Orthologs

ESTs: expressed sequence tags

CDS: coding sequences

SuSy: Sucrose Synthase

ScSuSy: Sugarcane Sucrose Synthase

PAL: Phenylalanine ammonia-lyase

ScPAL: Sugarcane Phenylalanine ammonia-lyase

CCR: Cinnamoyl-CoA reductase

COMT: Caffeic acid 3-O-methyltransferase

4CL: 4-coumarate-CoA ligase

TFBSs: Transcription Factor Binding Sites

TE: transposable elements

MCA: Multiple correspondence analysis

I2C-2: R gene locus

dog1: (delay of germination 1

ABRE: ABA-responsive elements

ABA: abscisic acid

Consent for publication: Not applicable

Availability of data and material

Genomic data is publicly available at NCBI under GenBank Bioproject PRJNA431722. Contig sequence, gene annotation, alignment with RNA-Seq reads and SAS are also available in a genome browser framework at http://sucest-fun.org/cgi-bin/cane_regnet/gbrowse2/gbrowse/microsoft_genome_moleculo_scga7/). The microarray data have been deposited in NCBI's Gene Expression Omnibus and are accessible through GEO Series accession number GSE124990. All data and scripts are also available at GigaDB [104] and in a Github repository [105].

Competing interests

The authors declare that they have no competing interests.

Funding

This work was funded by State of São Paulo Foundation and Microsoft Research (FAPESP grant n° 2012/51062-3) and State of São Paulo Foundation (FAPESP grants n° 2016/17545-8, 2014/50921-8, 2008/52146-0 and 2008/52074-0) under the BIOEN Program. Additional funding included awards from the National Science Foundation (DBI-1350041), and from the National Institutes of Health (R01-HG006677). Bioinformatic tools were run locally on the servers HELIX -IQ / Lab. Signal Transduction - and on the eScience Network - IME / FAPESP grant n° 2011 / 50761-2, CNPq, CAPES, NAP eScience - PRP – USP. GMS is a recipient of a CNPq Productivity Fellowship 304360/2014-7; MAVS is a recipient of a CNPq Productivity Fellowship (308197/2010-0); GRAM was supported by the FAPESP grant 2015/22993-7; JW was supported by the FAPESP Fellowships 2013/18322-4 and 2015/15346-5 and CNPq Fellowship 159094/2014-3; ALD is a recipient of a FAPESP Fellowship 2017/02270-6; MMO was a recipient of a CAPES

Fellowship DS-1454337; SSF was supported by the FAPESP Fellowships 2013/23048-9 and 2016/06917-1; MYN was supported by a FAPESP fellowship 2013/07467-1; FTC is a recipient of a FAPESP Fellowship 2017/02842-0; AMD is a recipient of a CNPq Productivity Fellowship (309566/2015-0); AP is a recipient of funding from the International Consortium for Sugarcane Biotechnology; US National Science Foundation IOS-0115903, and Georgia Agricultural Experiment Station.

Authors' contributions

Project leaders: GMS, MAVS and DH;

Sample collection and DNA extraction: CGL;

Genome sequencing and assembly: HL, MCS, GRAM, RP and BD;

Genome assembly supervision: DH;

Genome annotation: MAVS, GJW, MYNJ and FTC;

Saccharum spontaneum genome assembly: JZ, XZ, QZ and RM;

BWA-SW analysis: GJW;

BAC sequencing and assembly: MAVS, GJW, GTR, HB and SV;

Synteny analysis: AMD, RFS and GGS;

Reference-guided RNA-Seq Assembly: MYNJ;

Tissue-Specific Allelic Expression Analysis: MYNJ, CGL and PMA;

Phylogeny analysis: SSF and ALD;

SP80-3280 growth and maturation experiment: MSC, GMS, CGL and ALD

Co-expression analysis: ALD

Regulatory region analysis (TE and TFBS): MAVS, MMO, AMD, GMS, CTH and ALD;

SNP variants (SNVs) analysis: CK, HG and AP;

Organization and management of the author's contributions: CGL, ALD, GMS and MAVS;

Data availability (NCBI, Github and Sucest-fun): FTC;

All authors have read and approved the final version of the manuscript.

Acknowledgements

We are indebted to Andreia Prata, Vania Sedano, Nathalia de Setta, Joni Lima, Marcos Buckeridge, Eveline Tavares, Katia Scortecci, Anete Pereira de Souza, Sonia Vautrin and Hélène Bergès for contributions in BAC library construction, BAC selection or sequencing. We are indebted to the Sugarcane Genome Sequencing Initiative for useful discussions.

REFERENCES

1. FAOSTAT. Production/Crops, Food and Agriculture Organization of the United Nations - Statistics Division [Internet]. 2018. Available from: http://www.fao.org/faostat/en/#home

2. Long SP, Karp A, Buckeridge SC, Davis SC, Jaiswal D, Moore PH, et al. Feedstocks for biofuels and bioenergy. Bioenergy Sustain Bridg Gaps [Internet]. Paris Cedex: Scientific Committee on Problems of the Environment (SCOPE); 2015. p. 302–347. Available from: http://bioenfapesp.org/scopebioenergy/images/chapters/bioen-scope_chapter10.pdf

3. Kline KL, Msangi S, Dale VH, Woods J, Souza GM, Osseweijer P, et al. Reconciling food security and bioenergy: priorities for action. GCB Bioenergy. 2017;9:557–76.

4. Goldemberg J. Ethanol for a Sustainable Energy Future. Science. 2007;315:808–10.

5. Jaiswal D, De Souza AP, Larsen S, LeBauer DS, Miguez FE, Sparovek G, et al. Brazilian sugarcane ethanol as an expandable green alternative to crude oil use. Nat Clim Change. 2017;7:788–92.

6. Souza GM, Ballester MVR, de Brito Cruz CH, Chum H, Dale B, Dale VH, et al. The role of bioenergy in a climate-changing world. Environ Dev. 2017;23:57–64.

7. Souza GM, Victoria RL, Joly CA, Verdade LM. Bioenergy & sustainability: bridging the gaps. Paris Cedex: Scientific Committee on Problems of the Environment (SCOPE); 2015.

8. Souza GM, Filho RM. Industrial Biotechnology and Biomass: What Next for Brazil's Future Energy and Chemicals? Ind Biotechnol. 2016;12:24–5.

9. Vilela M de M, Del-Bem L-E, Van Sluys M-A, de Setta N, Kitajima JP, Cruz GMQ, et al. Analysis of three sugarcane homo/homeologous regions suggests independent polyploidization events of *Saccharum officinarum* and *Saccharum spontaneum*. Genome Biol Evol. 2017;evw293.

10. Jannoo N, Grivet L, Seguin M, Paulet F, Domaingue R, Rao PS, et al. Molecular investigation of the genetic base of sugarcane cultivars. Theor Appl Genet. 1999;99:171–84.

11. D'Hont A. Unraveling the genome structure of polyploids using FISH and GISH; examples of sugarcane and banana. Cytogenet Genome Res. 2005;109:27–33.

12. Thirugnanasambandam PP, Hoang NV, Henry RJ. The Challenge of Analyzing the Sugarcane Genome. Front Plant Sci [Internet]. 2018 [cited 2018 Aug 23];9. Available from: http://journal.frontiersin.org/article/10.3389/fpls.2018.00616/full

13. Garsmeur O, Droc G, Antonise R, Grimwood J, Potier B, Aitken K, et al. A mosaic monoploid reference sequence for the highly complex genome of sugarcane. Nat Commun [Internet]. 2018 [cited 2018 Aug 16];9. Available from: http://www.nature.com/articles/s41467-018-05051-5

14. Zhang J, Zhang X, Tang H, Zhang Q, Hua X, Ma X, et al. Allele-defined genome of the autopolyploid sugarcane Saccharum spontaneum L. Nat Genet. 2018;50:1565–73.

15. Waclawovsky AJ, Sato PM, Lembke CG, Moore PH, Souza GM. Sugarcane for bioenergy production: an assessment of yield and regulation of sucrose content. Plant Biotechnol J. 2010;8:263–76.

16. Goldemberg J, Coelho ST, Guardabassi P. The sustainability of ethanol production from sugarcane. Energy Policy. 2008;36:2086–97.

17. Welbaum GE, Meinzer FC. Compartmentation of solutes and water in developing sugarcane stalk tissue. Plant Physiol. 1990;93:1147–53.

18. Bonawitz ND, Chapple C. The genetics of lignin biosynthesis: connecting genotype to phenotype. Annu Rev Genet. 2010/09/03. 2010;44:337–63.

19. Himmel ME, Ding SY, Johnson DK, Adney WS, Nimlos MR, Brady JW, et al. Biomass recalcitrance: engineering plants and enzymes for biofuels production. Science. 2007/02/10. 2007;315:804–7.

20. Vettore AL. Analysis and Functional Annotation of an Expressed Sequence Tag Collection for Tropical Crop Sugarcane. Genome Res. 2003;13:2725–35.

21. Riaño-Pachón DM, Mattiello L. Draft genome sequencing of the sugarcane hybrid SP80-3280. F1000Research. 2017;6:861.

22. Parra G, Bradnam K, Korf I. CEGMA: a pipeline to accurately annotate core genes in eukaryotic genomes. Bioinformatics. 2007;23:1061–7.

23. Simão FA, Waterhouse RM, Ioannidis P, Kriventseva EV, Zdobnov EM. BUSCO: assessing genome assembly and annotation completeness with single-copy orthologs. Bioinformatics. 2015;31:3210–2.

24. Calsa Júnior T, Carraro DM, Benatti MR, Barbosa AC, Kitajima JP, Carrer H. Structural features and transcript-editing analysis of sugarcane (Saccharum officinarum L.) chloroplast genome. Curr Genet. 2004;46:366–73.

25. Shearman JR, Sonthirod C, Naktang C, Pootakham W, Yoocha T, Sangsrakru D, et al. The two chromosomes of the mitochondrial genome of a sugarcane cultivar: assembly and recombination analysis using long PacBio reads. Sci Rep [Internet]. 2016 [cited 2018 Jan 24];6. Available from: http://www.nature.com/articles/srep31533

26. Cardoso-Silva CB, Costa EA, Mancini MC, Balsalobre TWA, Canesin LEC, Pinto LR, et al. De Novo Assembly and Transcriptome Analysis of Contrasting Sugarcane Varieties. Gibas C, editor. PLoS ONE. 2014;9:e88462.

27. Vicentini R, Bottcher A, Brito M dos S, dos Santos AB, Creste S, Landell MG de A, et al. Large-Scale Transcriptome Analysis of Two Sugarcane Genotypes Contrasting for Lignin Content. Amancio S, editor. PLOS ONE. 2015;10:e0134909.

28. Nishiyama MY, Ferreira SS, Tang P-Z, Becker S, Pörtner-Taliana A, Souza GM. Full-Length Enriched cDNA Libraries and ORFeome Analysis of Sugarcane Hybrid and Ancestor Genotypes. PLOS ONE. 2014;9:e107351.

29. Suzek BE, Wang Y, Huang H, McGarvey PB, Wu CH, the UniProt Consortium. UniRef clusters: a comprehensive and scalable alternative for improving sequence similarity searches. Bioinformatics. 2015;31:926–32.

30. Ashburner M, Ball CA, Blake JA, Botstein D, Butler H, Cherry JM, et al. Gene Ontology: tool for the unification of biology. Nat Genet. 2000;25:25–9.

31. Veeckman E, Ruttink T, Vandepoele K. Are We There Yet? Reliably Estimating the Completeness of Plant Genome Sequences. Plant Cell. 2016;28:1759–68.

32. Nelson JC, Wang S, Wu Y, Li X, Antony G, White FF, et al. Single-nucleotide polymorphism discovery by high-throughput sequencing in sorghum. BMC Genomics [Internet]. 2011 [cited 2018 Jan 26];12. Available from: http://bmcgenomics.biomedcentral.com/articles/10.1186/1471-2164-12-352

33. Coleman HD, Yan J, Mansfield SD. Sucrose synthase affects carbon partitioning to increase cellulose production and altered cell wall ultrastructure. Proc Natl Acad Sci. 2009;106:13118–23.

34. Zhang J, Arro J, Chen Y, Ming R. Haplotype analysis of sucrose synthase gene family in three Saccharumspecies. BMC Genomics. 2013;14:314.

35. Rawat R, Schwartz J, Jones MA, Sairanen I, Cheng Y, Andersson CR, et al. REVEILLE1, a Myb-like transcription factor, integrates the circadian clock and auxin pathways. Proc Natl Acad Sci. 2009;106:16883–8.

36. Seo PJ, Ryu J, Kang SK, Park C-M. Modulation of sugar metabolism by an INDETERMINATE DOMAIN transcription factor contributes to photoperiodic flowering in Arabidopsis: Sugar and photoperiodic flowering. Plant J. 2011;65:418–29.

37. Papini-Terzi FS, Rocha FR, Vêncio RZ, Felix JM, Branco DS, Waclawovsky AJ, et al. Sugarcane genes associated with sucrose content. BMC Genomics. 2009;10:120.

38. Persia D, Cai G, Del Casino C, Faleri C, Willemse MT, Cresti M. Sucrose synthase is associated with the cell wall of tobacco pollen tubes. Plant Physiol. 2008;147:1603–18.

39. Brill E, van Thournout M, White RG, Llewellyn D, Campbell PM, Engelen S, et al. A Novel Isoform of Sucrose Synthase Is Targeted to the Cell Wall during Secondary Cell Wall Synthesis in Cotton Fiber. Plant Physiol. 2011;157:40–54.

40. Sewalt Vjh, Ni W, Blount JW, Jung HG, Masoud SA, Howles PA, et al. Reduced Lignin Content and Altered Lignin Composition in Transgenic Tobacco Down-Regulated in Expression of L-Phenylalanine Ammonia-Lyase or Cinnamate 4-Hydroxylase. Plant Physiol. 1997;115:41–50.

41. Rohde A. Molecular Phenotyping of the pal1 and pal2 Mutants of Arabidopsis thaliana Reveals Far-Reaching Consequences on Phenylpropanoid, Amino Acid, and Carbohydrate Metabolism. PLANT CELL ONLINE. 2004;16:2749–71.

42. Vanholme R, Storme V, Vanholme B, Sundin L, Christensen JH, Goeminne G, et al. A Systems Biology View of Responses to Lignin Biosynthesis Perturbations in Arabidopsis. Plant Cell. 2012;24:3506–29.

43. Ferreira SS, Hotta CT, Poelking VG de C, Leite DCC, Buckeridge MS, Loureiro ME, et al. Co-expression network analysis reveals transcription factors associated to cell wall biosynthesis in sugarcane. Plant Mol Biol. 2016;91:15–35.

44. Cunha CP, Roberto GG, Vicentini R, Lembke CG, Souza GM, Ribeiro RV, et al. Ethylene-induced transcriptional and hormonal responses at the onset of sugarcane ripening. Sci Rep [Internet]. 2017 [cited 2018 Aug 16];7. Available from: http://www.nature.com/articles/srep43364

45. Xu Z, Zhang D, Hu J, Zhou X, Ye X, Reichel KL, et al. Comparative genome analysis of lignin biosynthesis gene families across the plant kingdom. BMC Bioinformatics. 2009;10:S3.

46. Yilmaz A, Nishiyama MY, Fuentes BG, Souza GM, Janies D, Gray J, et al. GRASSIUS: A Platform for Comparative Regulatory Genomics across the Grasses. PLANT Physiol. 2009;149:171–80.

47. Domingues DS, Cruz GM, Metcalfe CJ, Nogueira FT, Vicentini R, de S Alves C, et al. Analysis of plant LTR-retrotransposons at the fine-scale family level reveals individual molecular patterns. BMC Genomics. 2012;13:137.

48. Kim C, Wang X, Lee T-H, Jakob K, Lee G-J, Paterson AH. Comparative Analysis of Miscanthus and Saccharum Reveals a Shared Whole-Genome Duplication but Different Evolutionary Fates. Plant Cell. 2014;26:2420–9.

49. Vieira MLC, Almeida CB, Oliveira CA, Tacuatiá LO, Munhoz CF, Cauz-Santos LA, et al. Revisiting Meiosis in Sugarcane: Chromosomal Irregularities and the Prevalence of Bivalent Configurations. Front Genet [Internet]. 2018 [cited 2018 Aug 27];9. Available from: https://www.frontiersin.org/article/10.3389/fgene.2018.00213/full

50. Wang J, Roe B, Macmil S, Yu Q, Murray JE, Tang H, et al. Microcollinearity between autopolyploid sugarcane and diploid sorghum genomes. BMC Genomics. 2010;11:261.

51. Zhang et al. Allele-defined genome of the autopolyploid sugarcane Saccharum spontaneum L. Accept Nat Genet. 2018;

52. Paterson AH, Bowers JE, Bruggmann R, Dubchak I, Grimwood J, Gundlach H, et al. The Sorghum bicolor genome and the diversification of grasses. Nature. 2009;457:551–6.

53. D'Hont A, Ison D, Alix K, Roux C, Glaszmann JC. Determination of basic chromosome numbers in the genus *Saccharum* by physical mapping of ribosomal RNA genes. Genome. 1998;41:221–5.

54. Liu Z, Adams KL. Expression Partitioning between Genes Duplicated by Polyploidy under Abiotic Stress and during Organ Development. Curr Biol. 2007;17:1669–74.

55. Ramírez-González RH, Borrill P, Lang D, Harrington SA, Brinton J, Venturini L, et al. The transcriptional landscape of polyploid wheat. Science. 2018;361:eaar6089.

56. Zhang Y, Liu Z, Khan AA, Lin Q, Han Y, Mu P, et al. Expression partitioning of homeologs and tandem duplications contribute to salt tolerance in wheat (Triticum aestivum L.). Sci Rep [Internet]. 2016 [cited 2018 Aug 16];6. Available from: http://www.nature.com/articles/srep21476

57. Liu Z, Xin M, Qin J, Peng H, Ni Z, Yao Y, et al. Temporal transcriptome profiling reveals expression partitioning of homeologous genes contributing to heat and drought acclimation in wheat (Triticum aestivum L.). BMC Plant Biol [Internet]. 2015 [cited 2018 Aug 16];15. Available from: http://www.biomedcentral.com/1471-2229/15/152

58. Dal-Bianco M, Carneiro MS, Hotta CT, Chapola RG, Hoffmann HP, Garcia AAF, et al. Sugarcane improvement: how far can we go? Curr Opin Biotechnol. 2012;23:265–70.

59. Illumina. FastTrack Services Long Reads Pipeline User Guide. 2013.

60. Myers EW. A Whole-Genome Assembly of Drosophila. Science. 2000;287:2196–204.

61. Chin C-S, Alexander DH, Marks P, Klammer AA, Drake J, Heiner C, et al. Nonhybrid, finished microbial genome assemblies from long-read SMRT sequencing data. Nat Methods. 2013;10:563–9.

62. de Setta N, Monteiro-Vitorello CB, Metcalfe CJ, Cruz GMQ, Del Bem LE, Vicentini R, et al. Building the sugarcane genome for biotechnology and identifying evolutionary trends. BMC Genomics. 2014;15:540.

63. Goodstein DM, Shu S, Howson R, Neupane R, Hayes RD, Fazo J, et al. Phytozome: a comparative platform for green plant genomics. Nucleic Acids Res. 2012;40:D1178–86.

64. Keller O, Kollmar M, Stanke M, Waack S. A novel hybrid gene prediction method employing protein multiple sequence alignments. Bioinforma Oxf Engl. 2011;27:757–63.

65. Eddy SR. Accelerated Profile HMM Searches. Pearson WR, editor. PLoS Comput Biol. 2011;7:e1002195.

66. Knudsen T, Knudsen B. CLC Genomics Benchwork 6 [Internet]. 2013. Available from: http://www.clcbio.com

67. Gotoh O. Direct mapping and alignment of protein sequences onto genomic sequence. Bioinformatics. 2008;24:2438–44.

68. Quinlan AR, Hall IM. BEDTools: a flexible suite of utilities for comparing genomic features. Bioinformatics. 2010;26:841–2.

69. Smit A, Hubley R, Green P. RepeatMasker Open-4.0 [Internet]. Available from: http://www.repeatmasker.org

70. Jurka J, Kapitonov VV, Pavlicek A, Klonowski P, Kohany O, Walichiewicz J. Repbase Update, a database of eukaryotic repetitive elements. Cytogenet Genome Res. 2005;110:462–7.

71. Majoros WH, Pertea M, Salzberg SL. TigrScan and GlimmerHMM: two open source ab initio eukaryotic gene-finders. Bioinforma Oxf Engl. 2004;20:2878–9.

72. Besemer J, Borodovsky M. GeneMark: web software for gene finding in prokaryotes, eukaryotes and viruses. Nucleic Acids Res. 2005;33:W451–4.

73. Haas BJ, Delcher AL, Mount SM, Wortman JR, Smith Jr RK, Hannick LI, et al. Improving the Arabidopsis genome annotation using maximal transcript alignment assemblies. Nucleic Acids Res. 2003;31:5654–66.

74. Slater GSC, Birney E. Automated generation of heuristics for biological sequence comparison. BMC Bioinformatics. 2005;6:31.

75. Camacho C, Coulouris G, Avagyan V, Ma N, Papadopoulos J, Bealer K, et al. BLAST+: architecture and applications. BMC Bioinformatics. 2009;10:421.

76. Haas BJ, Salzberg SL, Zhu W, Pertea M, Allen JE, Orvis J, et al. Automated eukaryotic gene structure annotation using EVidenceModeler and the Program to Assemble Spliced Alignments. Genome Biol. 2008;9:R7.

77. Jones P, Binns D, Chang H-Y, Fraser M, Li W, McAnulla C, et al. InterProScan 5: genome-scale protein function classification. Bioinformatics. 2014;30:1236–40.

78. Conesa A, Gotz S, Garcia-Gomez JM, Terol J, Talon M, Robles M. Blast2GO: a universal tool for annotation, visualization and analysis in functional genomics research. Bioinformatics. 2005;21:3674–6.

79. Han F, Peng Y, Xu L, Xiao P. Identification, characterization, and utilization of single copy genes in 29 angiosperm genomes. BMC Genomics. 2014;15:504.

80. Kent WJ. BLAT--the BLAST-like alignment tool. Genome Res. 2002;12:656–64.

81. R Core Team. R: A Language and Environment for Statistical Computing [Internet]. Vienna, Austria; 2014. Available from: http://www.R-project.org

82. Edgar RC. MUSCLE: multiple sequence alignment with high accuracy and high throughput. Nucleic Acids Res. 2004;32:1792–7.

83. Kim D, Pertea G, Trapnell C, Pimentel H, Kelley R, Salzberg SL. TopHat2: accurate alignment of transcriptomes in the presence of insertions, deletions and gene fusions. Genome Biol. 2013;14:R36.

84. Li H, Handsaker B, Wysoker A, Fennell T, Ruan J, Homer N, et al. The Sequence Alignment/Map format and SAMtools. Bioinforma Oxf Engl. 2009;25:2078–9.

85. Finn RD, Coggill P, Eberhardt RY, Eddy SR, Mistry J, Mitchell AL, et al. The Pfam protein families database: towards a more sustainable future. Nucleic Acids Res. 2016;44:D279–85.

86. Xu Z, Zhang D, Hu J, Zhou X, Ye X, Reichel KL, et al. Comparative genome analysis of lignin biosynthesis gene families across the plant kingdom. BMC Bioinformatics. 2009;10 Suppl 1:S3.

87. Röther D, Poppe L, Morlock G, Viergutz S, Rétey J. An active site homology model of phenylalanine ammonia-lyase from P. crispum. Eur J Biochem. 2002;269:3065–75.

88. Calabrese JC, Jordan DB, Boodhoo A, Sariaslani S, Vannelli T. Crystal structure of phenylalanine ammonia lyase: Multiple helix dipoles implicated in catalysis. Biochemistry. 2004;43:11403–16.

89. Pilbák S, Tomin A, Rétey J, Poppe L. The essential tyrosine-containing loop conformation and the role of the C-terminal multi-helix region in eukaryotic phenylalanine ammonia-lyases. FEBS J. 2006;273:1004–19.

90. Thompson JD, Higgins DG, Gibson TJ. CLUSTAL W: improving the sensitivity of progressive multiple sequence alignment through sequence weighting, position-specific gap penalties and weight matrix choice. Nucleic Acids Res. 1994;22:4673–80.

91. Kumar S, Stecher G, Tamura K. MEGA7: Molecular Evolutionary Genetics Analysis Version 7.0 for Bigger Datasets. Mol Biol Evol. 2016;33:1870–4.

92. Zhang Z, Wood WI. A profile hidden Markov model for signal peptides generated by HMMER. Bioinforma Oxf Engl. 2003;19:307–8.

93. Shahmuradov IA, Umarov RKh, Solovyev VV. TSSPlant: a new tool for prediction of plant Pol II promoters. Nucleic Acids Res. 2017;gkw1353.

94. Bailey TL, Boden M, Buske FA, Frith M, Grant CE, Clementi L, et al. MEME Suite: tools for motif discovery and searching. Nucleic Acids Res. 2009;37:W202–8.

95. Claeys M, Storms V, Sun H, Michoel T, Marchal K. MotifSuite: workflow for probabilistic motif detection and assessment. Bioinformatics. 2012;28:1931–2.

96. Khan A, Fornes O, Stigliani A, Gheorghe M, Castro-Mondragon JA, van der Lee R, et al. JASPAR 2018: update of the open-access database of transcription factor binding profiles and its web framework. Nucleic Acids Res. 2018;46:D260–6.

97. Russo PST, Ferreira GR, Cardozo LE, Bürger MC, Arias-Carrasco R, Maruyama SR, et al. CEMiTool: a Bioconductor package for performing comprehensive modular co-expression analyses. BMC Bioinformatics [Internet]. 2018 [cited 2018 Aug 16];19. Available from: https://bmcbioinformatics.biomedcentral.com/articles/10.1186/s12859-018-2053-1

98. Alexa A, Rahnenfuhrer J. topGO: Enrichment Analysis for Gene Ontology.

99. Shannon P. Cytoscape: A Software Environment for Integrated Models of Biomolecular Interaction Networks. Genome Res. 2003;13:2498–504.

100. Li H, Durbin R. Fast and accurate long-read alignment with Burrows-Wheeler transform. Bioinforma Oxf Engl. 2010;26:589–95.

101. McCormick RF, Truong SK, Sreedasyam A, Jenkins J, Shu S, Sims D, et al. The Sorghum bicolor reference genome: improved assembly, gene annotations, a transcriptome atlas, and signatures of genome organization. Plant J Cell Mol Biol. 2018;93:338–54.

102. Lê S, Josse J, Husson F. FactoMineR : An R Package for Multivariate Analysis. J Stat Softw [Internet]. 2008 [cited 2017 Nov 30];25. Available from: http://www.jstatsoft.org/v25/i01/

103. Josse J, Husson F. missMDA : A Package for Handling Missing Values in Multivariate Data Analysis. J Stat Softw [Internet]. 2016 [cited 2017 Nov 30];70. Available from: http://www.jstatsoft.org/v70/i01/

104. Souza GM, Van Sluys M-A, Lembke CG, Margarido GRA, et al. Supporting data for "Assembly of the 373K gene space of the polyploid sugarcane genome reveals reservoirs of functional diversity in the world's leading biomass crop." GigaScience Database 2019. http://dx.doi.org/10.5524/100655

105. Souza GM, Van Sluys M-A, Lembke CG, Margarido GRA, et al. Github repository for "Assembly of the 373K gene space of the polyploid sugarcane genome reveals reservoirs of functional diversity in the world's leading biomass crop." Available from: https://github.com/sp80-3280-genome

Table 1 – Genome sequencing: Technology and assembly details and gene prediction features.

	Description	Genomic DNA	BAC clones
Sequencing and assembly data	Sequencing Data	26 Illumina synthetic long-read libraries	Single end Roche 454 of BAC library clones
	Total Sequence	19 Gb	6.6 Gb
	Genome coverage	1.9 x	0.66 x
	Read length Min/Max/Mean	1,500 bp / 22,904 bp / 4,930 bp	8 bp / 2611 bp / 368.5 bp
	Assembler Software	Celera Assembler (Overlap Graph)	PHRAP/CONSED
	Total reads used in assembly	3,857,849	17,894,306
	Total assembly size	4.26 Gb	49.6 Mb
	Number of unitigs/contigs + singletons	450,609	463
	Contigs Length Min/Max/Mean	1,500 bp / 468,011 bp / 9,452 bp	11,723 bp / 235,533 bp / 107,129 bp
	NG50	41,394 bp	109,618 bp
	N50	13,157 bp	N/A
Gene prediction features	# genes	373,869	3,550
	# transcripts	374,774	-
	# exons	1,035,764	13,132
	Average GC content	43.20%	44.99%
	Average # exons per gene	2.8	3.7
	Average exon size [bp]	291	271.8
	Median exon size [bp]	171	154
	Average intron size [bp]	352.6	539.2
	Median intron size [bp]	132	139
	Average gene size [bp] with UTR	1,437.80	2,429.20
	Median gene size [bp] with UTR	806	1,260.50
	Average gene size [bp] without UTR	1,318.80	2,351.30
	Median gene size [bp] without UTR	771	1,199.50
	Average gene density (kb per gene)	11.4	14

Figure captions

Fig. 1 – Frequency histogram of Expressed Sequence Tags (ESTs) and Core Eukaryotic Genes Mapping Approach (CEGMA) regions alignment on Sugarcane genome assembly. For 127,940 aligned ESTs, 106,133 (84.9%) show 2 up to 30 matches on the genome (**A**), while for CEGMA regions, 205 (87.2%) range from 2 to 17 matches on the genome (**B**). SPALN v 2.3.3 [67] was used for alignment.

Fig. 2 – Gene copy number estimation. (**A**) Distribution of copy counts for putative single-copy genes in diploid grasses. From the 2,051 single-copy genes in sorghum, rice and *Brachypodium,* 1,592 single-copy genes matched to at least one sugarcane predicted gene. More than 99.9% of the aligned single-copy genes are present between one and 15 times in the sugarcane assembly. (**B**) Copy differentiation between sugarcane coding sequences (CDS) and upstream regions, based on pairwise sequence alignment of gene clusters. Genetic dissimilarity increases with increasing distance from the translation start site. (**C**) Indel length distribution in sugarcane putative homo(eo)logs. Frame preserving indels are more common than frameshifts for this set of genes.

Fig. 3 – Homo(eo)log expression: The percentage frequency of sugarcane genes plotted against the total number of homo(eo)logs per gene and the number of expressed homo(eo)logs per gene. Genes with cDNAs aligned with FPKM > 1 were considered expressed. Plots show sense (**A**) and antisense (**B**) transcripts. Reads from Ion PGM Sequencing were used and strand orientation is maintained [28].

Fig. 4 – Phylogeny, putative regulatory regions and expression of sucrose synthase (SuSy) and phenylalanine-ammonia lyase (PAL) gene family. Phylogenetic analysis of (**A**) SuSy and (**B**) PAL genes from SP80-3280, R570, *S. spontaneum,* and sorghum. SuSy sequences from *Saccharum* ssp [34] were also included. For both SuSy and PAL, nucleotide sequences (CDS) were aligned with CLUSTALW [90] software in MEGA 7.0 [91] and maximum likelihood trees were constructed with 1,000 bootstraps. Core promoter analysis (gray columns in C and D) using TSSPlant [93] suggests ScSuSy2 (**C**) and most ScPAL (**D**) as TATA-less (absence of black squares). Transcription factor binding sites (TFBS) prediction (colored symbols in **C** and **D**) using MEME [94] and MotifSampler [95] suggest specific motif for each group (ScSuSy1, ScSuSy2, ScSuSy5 and PAL I, PAL III, PAL Va and PAL Vb). The three SP80-3280 PAL genes marked (* in **D**) are present in the same contig. Transposable elements (TEs) were identified within 10 kb upstream from the gene (**C** and **D**). Heatmap analysis of RNA-Seq data [28] (expression profile in **C** and **D**) shows more pronounced expression in SP80-3280 internodes (I1 and I5) of ScSuSy1, ScSuSy2, ScSuSy5 and PAL from group V. RNA-Seq of leaf tissues (L) indicates more pronounced expression of ScPAL from groups II and III. ScSuSy3 presents high numbers of TFBS and TE and low expression in all samples.

Fig. 5 – SNP variants. Alignment of sugarcane contigs to the genic regions of sorghum chromosomes (chromosome 1 is on top and 10 is at the bottom). X and Y axes indicate physical distance on each chromosome (mega base pairs, Mb) and the number of single nucleotide variants compared to the sorghum reference genome, respectively. Each dot indicates sorghum genes matching two or more sugarcane contigs.

Fig. 6 – Pseudoassembly of contigs. Multiple correspondence analysis (MCA) with hierarchical clustering of the SP80-3280 assembly against the *S. spontaneum* tetraploid AP85-441 homo(eo)log-resolved assembly [14] and the R570 [13] monoploid genome. **A**: SP80-3280 contigs best hits against AP85-441 and R579 chromosomes and corresponding size of the preliminary scaffolds; Cluster = hierarchical cluster from the MCA. **B** and **C**: Circos plot of the proportion of proteins from SP80-3280 (classified into one of the 6 clusters or as 'non-clustered') that align to the AP85-441 and R570 putative chromosomes, respectively.

Second revision Figure 1

Click here to access/download;Figure;Second revision Fig 1 new.pdf

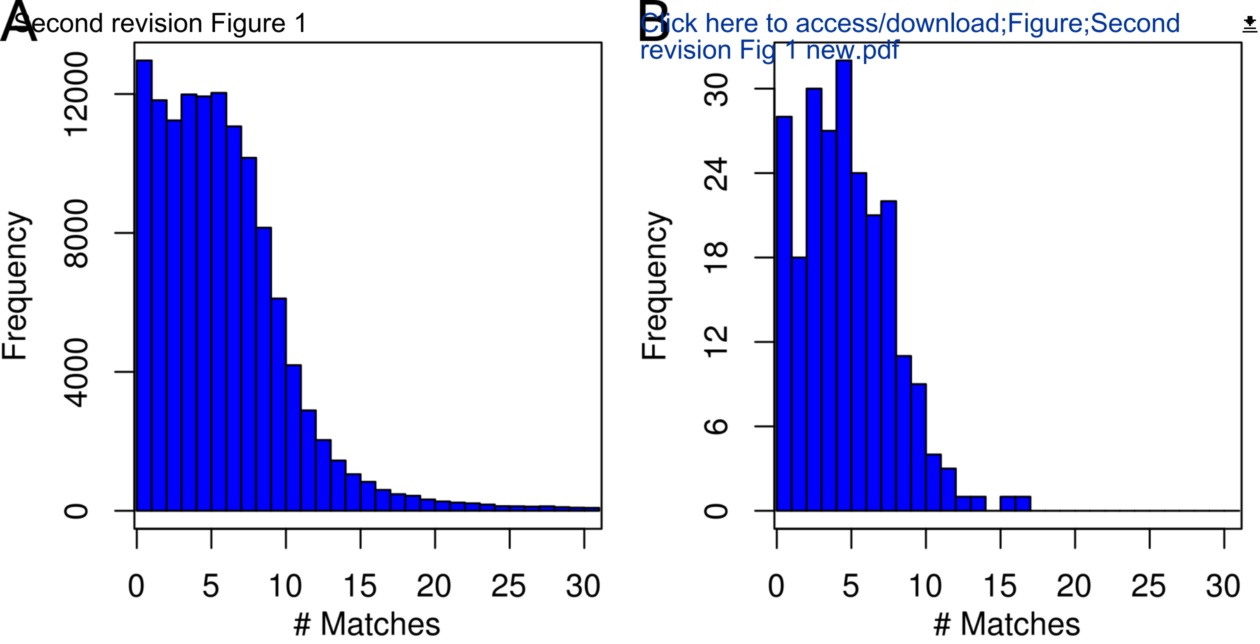

Second revision Figure 2
Click here to access/download;Figure;Second revision Fig 2.pdf

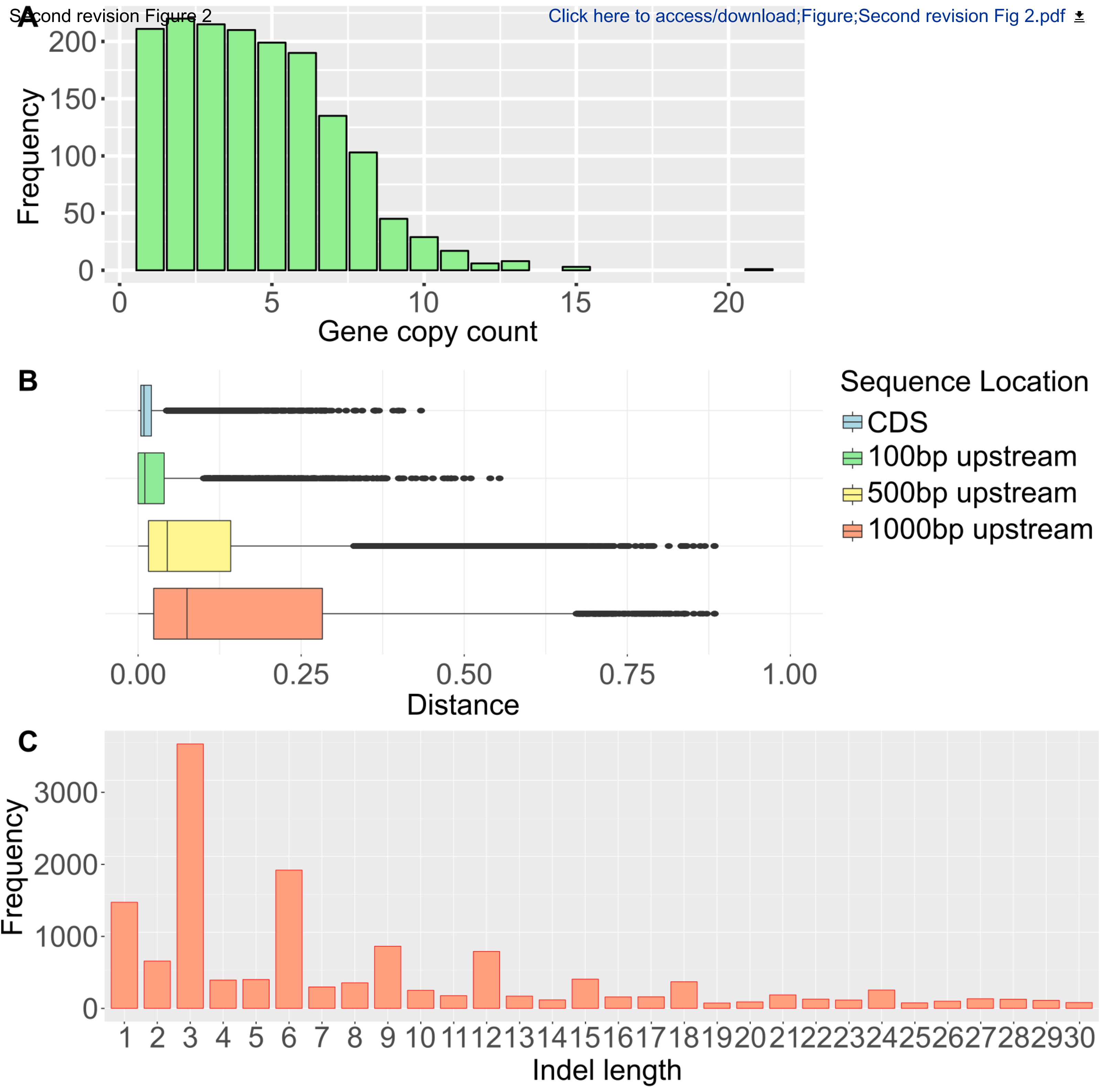

Second revision Figure 3

Click here to access/download;Figure;Second revision Fig 3.pdf

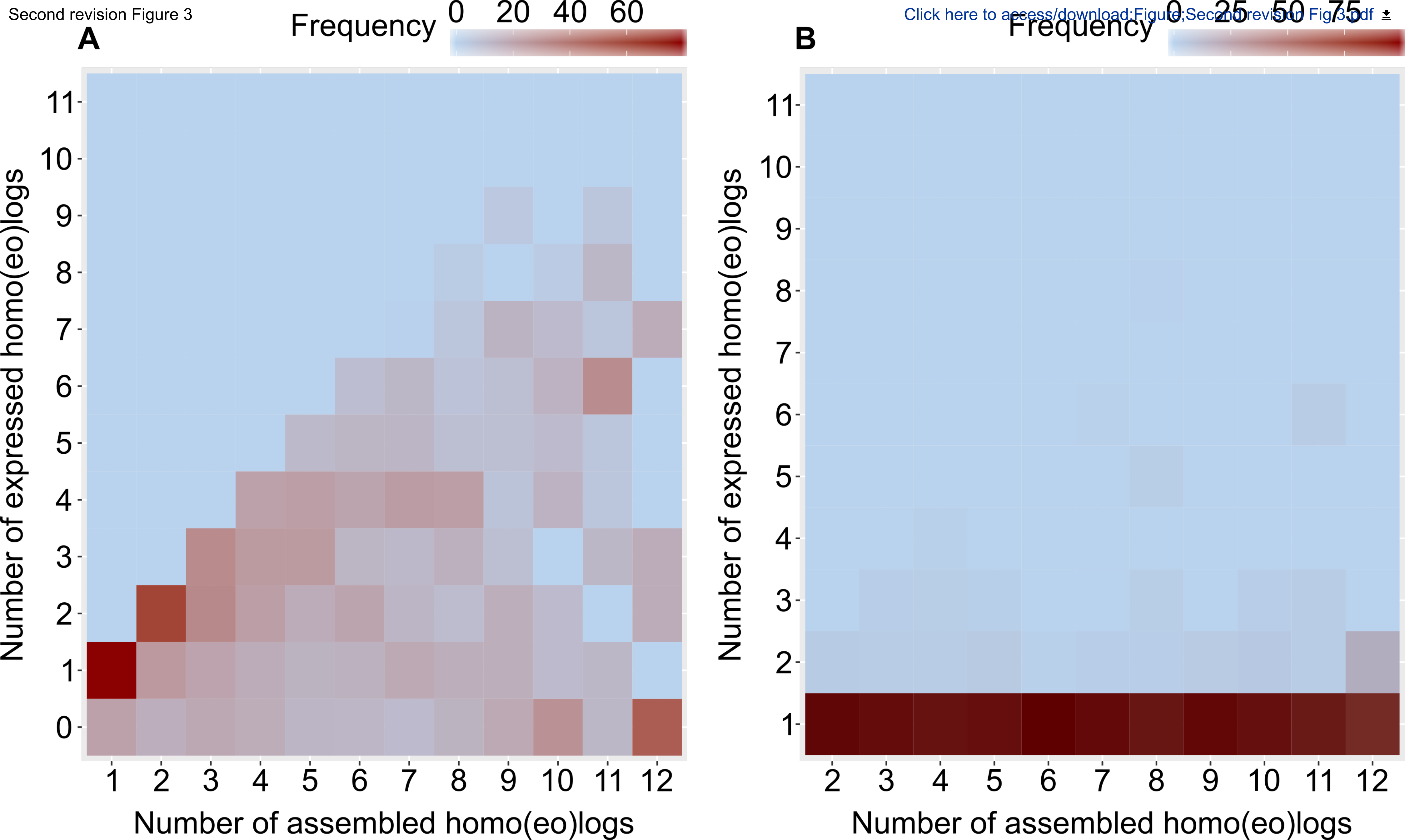

Second revision Figure 4

Click here to access/download;Figure;Second revision Fig 4 new.pdf

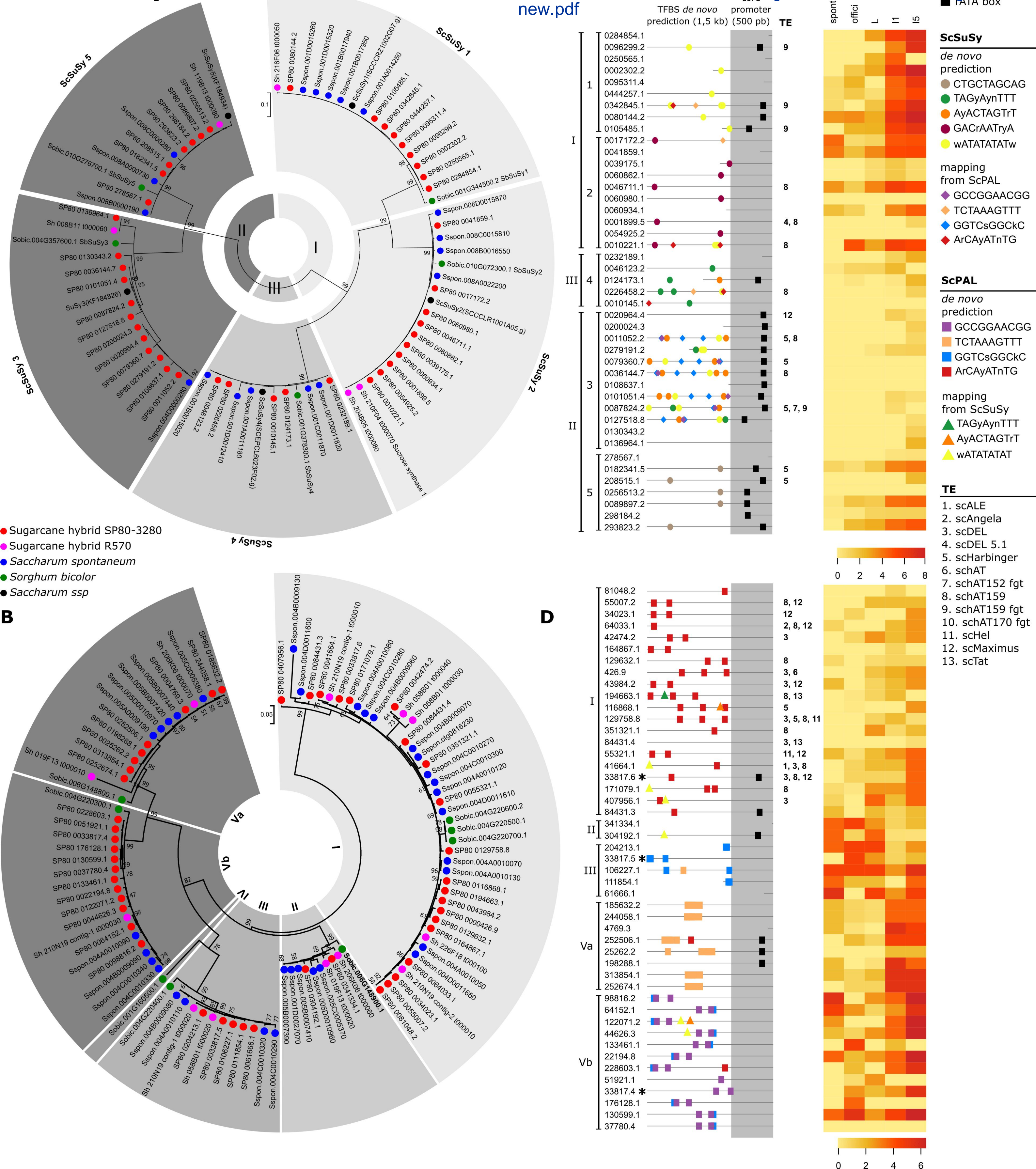

Second revision Figure 5

Click here to access/download;Figure;Second revision Fig 5.pdf

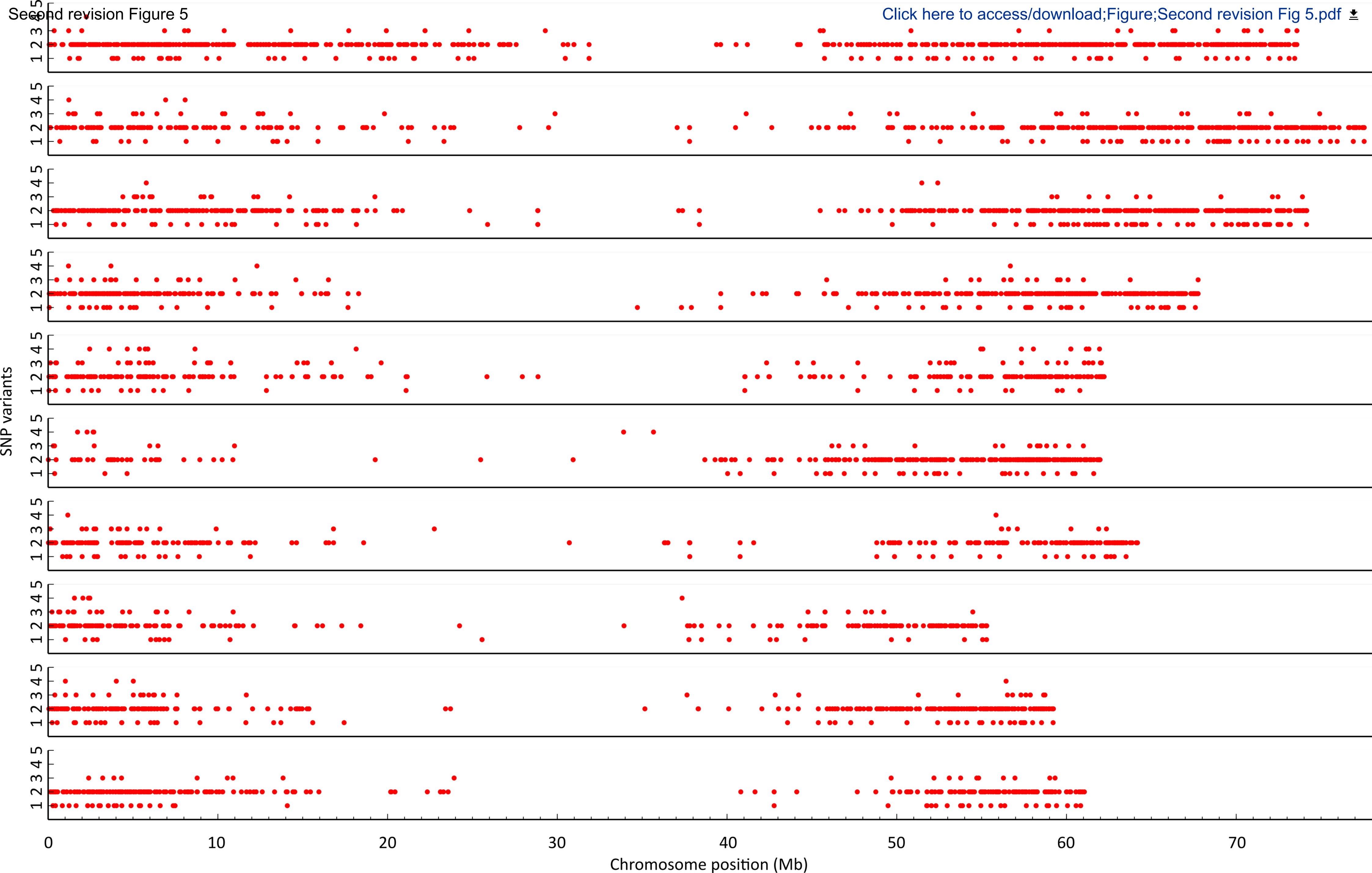

Second revision Figure 6

Click here to access/download;Figure;Second revision Fig 6.pdf

A

Cluster	Number of Contigs	bp	Chromosomal Correspondence	
			S. spontaneum	R570
1	60,150	567,792,642	4	4
2	61,705	574,401,531	1	1
3	87,155	823,254,612	7, 8	8, 9 10
4	90,152	896,362,990	5, 6	5, 6, 7
5	63,996	679,392,733	2	2
6	55,313	565,012,329	3	3
**Total**	**418,471**	**4,106,216,837**	-	-
**Original**	**450,609**	**4,259,506,050**	-	-

B

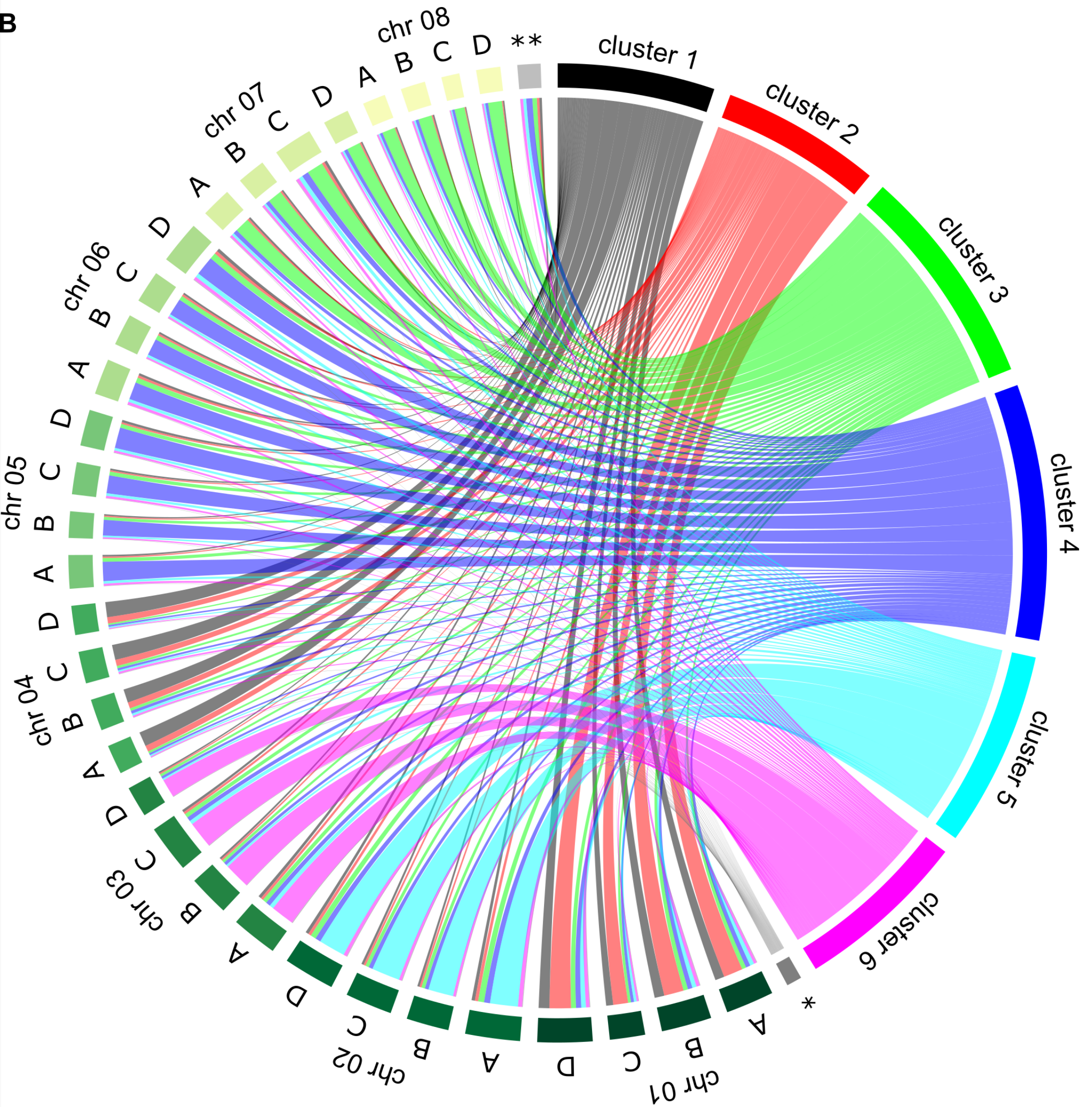

C

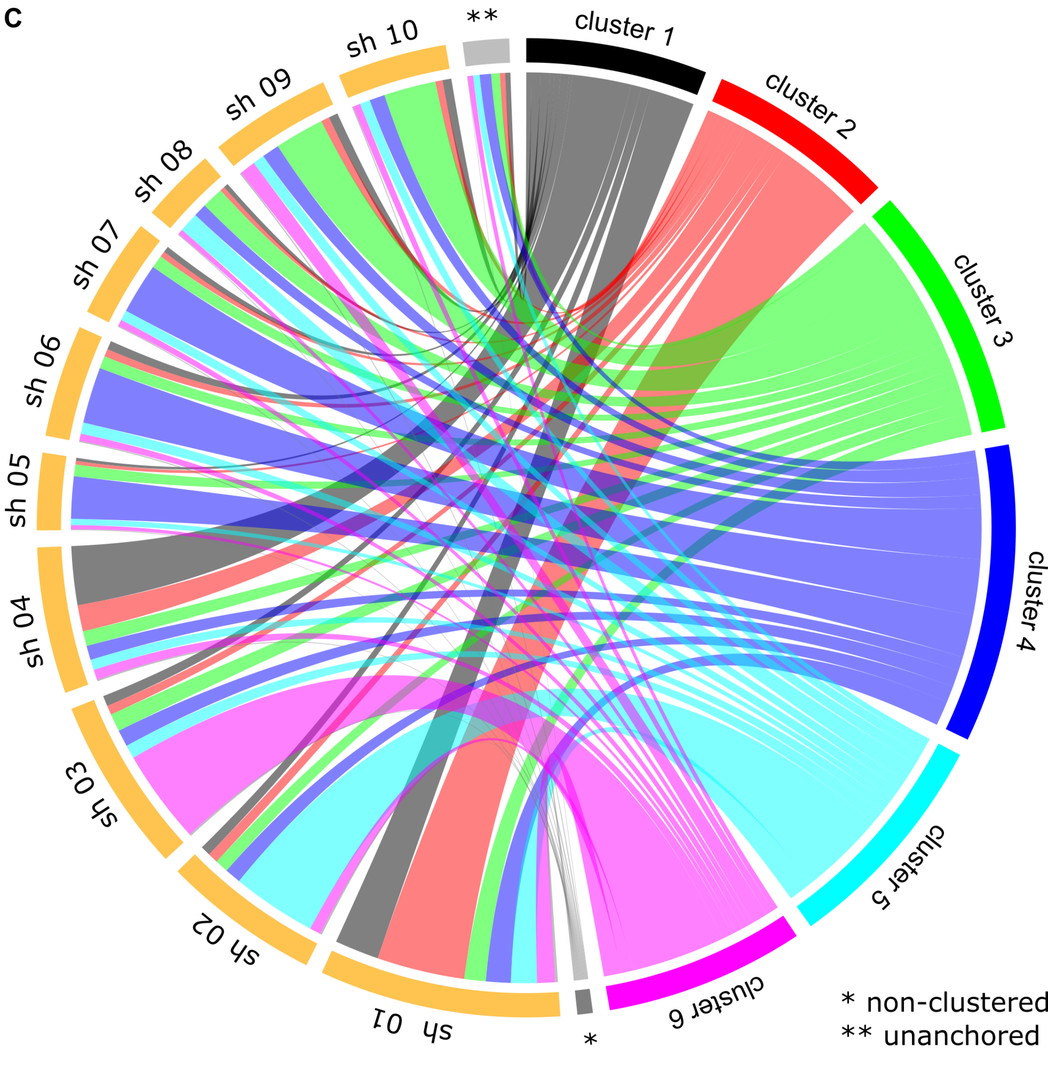

Click here to access/download
Supplementary Material
Second revision GIGA-D-19-00013 Additional file 1.docx

Second revision Response letter
Click here to access/download;Personal Cover;Second revision GIGA-D-19-00013 Response letter.docx

August 20th, 2019

GIGA-D-19-00013

Assembly of the 373K gene space of the polyploid sugarcane genome reveals reservoirs of functional diversity in the world's leading biomass crop

Glaucia Mendes Souza, Ph.D; Marie-Anne Van Sluys, Ph.D; Carolina Gimiliani Lembke, Ph.D; Hayan Lee, Ph.D; Gabriel Rodrigues Alves Margarido, Ph.D; Carlos Takeshi Hotta, Ph.D; Jonas Weissmann Gaiarsa, Ph.D; Augusto Lima Diniz, Ph.D; Mauro de Medeiros Oliveira, Ph.D; Sávio de Siqueira Ferreira, Ph.D; Milton Yutaka Nishiyama-Jr, Ph.D; Felipe ten Caten, Ph.D; Geovani Tolfo Ragagnin, MSc; Pablo de Morais Andrade, Ph.D; Robson Francisco de Souza, Ph.D; Gianlucca Gonçalves Nicastro, Ph.D; Ravi Pandya, BS.c; Changsoo Kim, Ph.D; Hui Guo, Ph.D; Alan Mitchell Durham, Ph.D; Monalisa Sampaio Carneiro, Ph.D; Jisen Zhang, Ph.D; Qing Zhang, Ph.D; Qing Zhang, Ph.D; Ray Ming, Ph.D; Michael Schatz, Ph.D; Bob Davidson; Andrew Paterson, Ph.D; David Heckerman, Ph.D

Dear Dr. Hans Zauner
Assistant Editor
Gigascience

We thank the editor and the reviewer. We declare that we have responded to all suggestions. A point-by-point response to each comment is presented. The revised version of our manuscript, in addition to a new Fig 1 (former Fig S.4), Fig.4 (former Fig.3) and Additional file 1, as well as all revised files (as suggested by the reviewer) have been uploaded.

Sincerely,

Glaucia Mendes Souza
Full Professor
Institute of Chemistry
University of São Paulo

Marie-Anne Van Sluys
Full Professor
Biosciences Institute
University of São Paulo

Editor’s comment:

We have divided the editor’s comments in three parts:

1) In summary, the reviewer and I agree that this work is a big step forward for sugarcane genomics, but I also agree with the reviewer that the completeness for the gene-space assembly should not be overstated. The reviewer makes useful suggestions to correct this, which I support ("1. moving some statements in the results section to the discussion; 2. including Fig S4 into the main body of the manuscript and 3. choose language which is a little less certain about the comprehensiveness/completeness of the gene space.").

2) The reviewer has many other useful comments for further improvement, from which I wish to highlight the practical suggestions to improve data sharing. The reviewer is also correct that, at GigaScience, reviewers need to be given access to all resources before publication, and all data needs to be released publicly at the point of publication, including the data hosted at SUCEST-FUN.

3) The other reviewer, Nils Stein, was unfortunately not available at this time to re-review, but we feel that his questions as to the assembly quality of the 5' and 3' region of genes could be addressed in more detail in the manuscript itself. In particular, the coverage plot placed in the response to reviewers will be useful for readers and should form part of the manuscript/supplementals.

Response: We appreciate the editor's comment and have changed the manuscript accordingly as follows:
1) (*i*) We have moved the suggested statements in the results section to the discussion; (*ii*) have included former Fig S4 as Fig 1 in the main body of the manuscript; (*iii*) and we have accepted the reviewer’s suggestion in “diluting” down our genome completeness statement. None of the words (comprehensiveness/completeness) are mentioned in the revised manuscript.

2) We now provide to the reviewer total access to data hosted at Github and SUCEST-FUN.

3) In the first review, the reviewer requested “an assessment of <u>sequence quality</u> in the 5`and 3`regions”. We did access sequence quality for all bases in all reads and, as presented in L119-120 and Additional file 1: Fig. S1, we declare that >99% of bases have >99% of accuracy. Furthermore, we accept the editor’s suggestion and have added more detail in the manuscript, as follows:

L119-121: "with >99% of bases having >99% accuracy (Additional file 1: Fig. S1), which assure the sequence quality of genes (to be predicted) and intergenic regions (which include the 5' and 3' region of genes). "

We have compared the assembled contigs to several data sets for validation (Sugarcane BACs, Sorghum CDS, CEGMA, and BUSCO), as described in the manuscript, and the data supports the assembly. Finally, we accept the editor's suggestion and have included the coverage plot (previously placed in the response to reviewers) as Fig S11 in the Additional file 1. Therefore, we have included the following sentence in the methods section:

L437-440: “In order to identify problematic regions, after the assembly step, we have assessed the assembled contigs using a read coverage analysis by mapping reads back to contigs. After sorting contigs from highest coverage to lowest, we found that only 0.1 Gbp of contigs had very high coverage (Additional file 1: Fig. S11).”

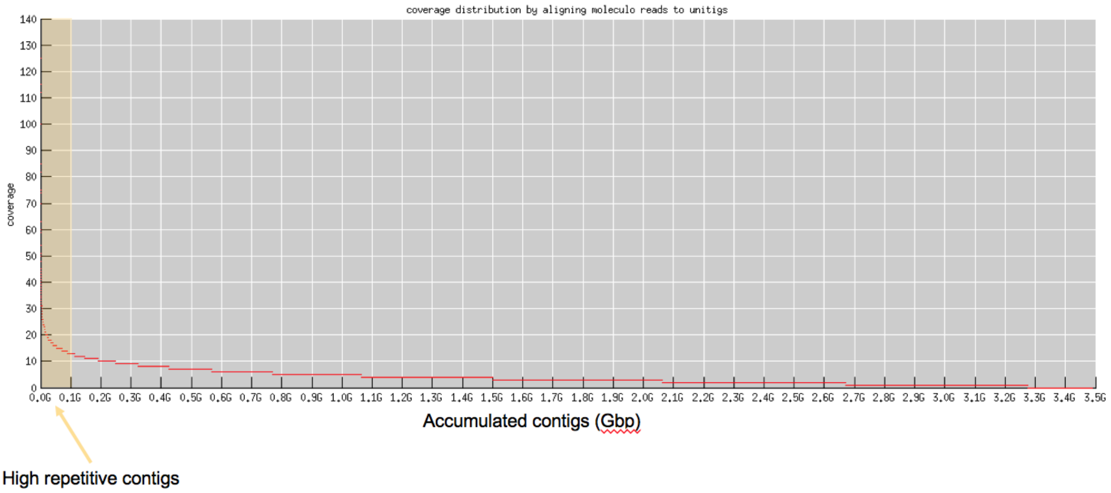

Fig S11 – Synthetic long read coverage plot: The reads were mapped back to the contigs. After sorting contigs from highest coverage to lowest, only 0.1 Gbp of contigs had very high coverage which represents highly repetitive sequences.

Main Concerns

Reviewer: Some of my worries would be allayed if 1) statements in the results section, which draw conclusions from these number, were moved to the discussion; 2) Fig S4 was brought into the main body of the manuscript and 3) choose language which is a little less certain about the comprehensiveness/completeness of the gene space.

Response:
1): We accept the reviewer's suggestion, have revisited the manuscript and moved the statements from the results to the discussion.
2): Fig S4 was brought into the main body of the manuscript and is now Figure 1.
3): We are aware that not all hom(eo)logous were resolved and have made the following changes:

- L59-61: "This assembly represents a large step towards a whole genome assembly of a commercial sugarcane cultivar. It includes a rich diversity of genes and homo(eo)logous resolution for a representative fraction of the gene space, relevant to improve biomass and food production.".
- L114-117: "In the assembly of 4.26 GB, 373,869 putative genes and promotor regions were predicted. For a large fraction of the gene space, an average of 6 sugarcane haplotypes, putatively homo(eo)logs, were identified. This is the first release of an assembly of such a giant hybrid polyploid genome with part of the putatively homo(eo)logs resolved and their potential regulatory regions.
- L368-369: "These differences highlight the importance of our assembly which discriminates homo(eo)logs for most genes".
- L474-475: "The assembly was accessed for the presence of the 1,440 core genes from the Plantae lineage of Benchmarking Universal Single-Copy Orthologs (BUSCO)"

Reviewer: Move the following statements from the results to the discussion:
- L126 "Several indicators support eh comprehensiveness of the SP80-3280 gene space"
- L140-141 - "The number of genes, high quality of alignments, and the following analysis indicates that the assembly provides a high-quality resolution of homo(eo)logous genes."

Response: We removed the first sentence from the revised manuscript. The second sentence was moved to discussion, as follows:
L353-356: "The total number of predicted genes, the high quality of alignments and the detection of more than one copy for single-copy genes in diploid grasses indicates that the assembly provides homo(eo)logous resolution for a large fraction of the gene space (~87%)."

Reviewer: "Dilute" down the following statements in the discussion: L349-L351: "The comparison against different sets of genes (sorghum, CEGMA, BUSCO, mitochondrial and chloroplast) supported the comprehensiveness of the gene space."

Response: We have changed the text as follows:
L347-349: "The comparison against different sets of genes (sorghum, CEGMA, BUSCO, mitochondrial and chloroplast) shows that the gene space assembly contains the majority of the genes queried in at least one copy."

Moreover, we have added the following in line L351-353: "We also detected that single-copy genes in diploid grasses are present in 2-6 and up to 15 copies. These findings agree with the predicted 8 to 14 copies for S. spontaneum, depending on the cytotypes, and for modern sugarcane varieties [53]."

Reviewer: Provide improved consistency/clarity for the following:

- L152 and L360: when referring to the number of homeologs identified in the assembly the authors tend to overstating the number when reporting "up to 15". Be consistent with L49, L159 which more accurately defines this as 2-6 and up to 15.

Response: We accept the reviewer's suggestion and have changed the text as follows:

- L151-154: "84.9% of ESTs (106,133) show 2-8 and up to 30 matches on the genome, reflecting the presence of the majority of putative homo(eo)logs (**Fig. 1A**). This result is similar to the search of CEGMA matches against the genome itself using BLASTn. From 235 sequences completely or partially covering CEGMA proteins, 205 have 2-8 and up to 17 matches on the genome (**Fig. 1B**)."
- L360-362: "Single-copy genes from diploid grasses correspond to mostly 2-6 copies (up to 15) of sugarcane genes in our SP80-3280 assembly and nucleotide differences are present mainly in the upstream regulatory region."

Reviewer: The authors refer to single-copy genes in several places. However, it is hard to know where these were derived and how many there are. L131 and L468 both refer to 1,440 from BUSCO, whereas L541 refers to 2,051 and Fig1A refers to 1,592. I suspect the 1,592 referred to in Fig 1A is the same 2,051 detailed on L541 but excluded single-copy genes with no hits to the assembly (i.e. 459 single-copy genes with no hits). Please clarify, include the number of single-copy genes with no hits and discuss reasons for single-copy genes not hitting the assembly.

Response: The reviewer understood correctly. We have used 'single-copy genes in diploid grasses' every time we refer to the set of genes that are single copy in *Sorghum bicolor*, *Oryza sativa* and *Brachypodium*, as follows:

L48: "The alignment of single-copy genes in diploid grasses to the putative genes, …"
L156-157: "Single-copy genes in diploid grasses (sorghum, rice and Brachypodium) are present in up to 15 copies in sugarcane …"
L163: "The SP80-3280 gene series that correspond to single-copy genes in diploid grasses showed expression of …"
L295-296: "Further, 1,334 SNVs that differentiate sugarcane from sorghum in 585 single-copy genes in diploid grasses include frameshifts"
L351-352: "We also detected that single-copy genes in diploid grasses are present in 2-6 and up to 15 copies."
L354-355: "and the detection of more than one copy for single-copy genes in diploid grasses indicates that"
L360-361: "Single-copy genes from diploid grasses correspond to mostly 2-6 copies (up to 15) of sugarcane genes …"
L594-595: "… find the number of putative expressed homo(eo)logs for each single-copy genes in diploid grasses, …"

We have also edited the Fig. 2 caption to include how many single-copy genes in diploid grasses matched to our assembly, as follows:

L1066-1073: “**Gene copy number estimation.** (**A**) Distribution of copy counts for putative single-copy genes in diploid grasses. From the 2,051 single-copy genes in sorghum, rice and *Brachypodium,* 1,592 single-copy genes matched to at least one sugarcane predicted gene. More than 99.9% of the aligned single-copy genes are present between one and 15 times in the sugarcane assembly. (**B**) Copy differentiation between sugarcane coding sequences (CDS) and upstream regions, based on pairwise sequence alignment of gene clusters. Genetic dissimilarity increases with increasing distance from the translation start site. (C) Indel length distribution in sugarcane putative homo(eo)logs. Frame preserving indels are more common than frameshifts for this set of genes.”

Regarding the number of single-copy genes (459) with no hits in the sugarcane assembly, we have two hypothesis. 1) According to Han et al. [79], the authors identified 6761, 9995 and 3987 single-copy genes for *S. bicolor*, *O. sativa* and *B. distachyon*, respectively. As stated in the methods section, we selected 2051 single-copy genes shared by these species. For instance, a single-copy gene in *S. bicolor* might not be present in *O. sativa* possible due deletion or gene duplication; in this case, it’s no longer considered a single-copy gene. Specifically, genes with no hits in the sugarcane assembly might indicate deletions during evolution. 2) Although we exploited long synthetic reads, it is still a big challenge to assemble one contig per chromosome. So, the gene may be spread to multiple contigs. That is a limitation of the technology at this time.

Reviewer: If sugarcane is an interspecific hybrid between S. officinarum and S. spontaneum then I assume two is the lower-bound for the number of homeologues - one from each parent? Can the authors discuss and cite relevant works regarding the high or low level of hom(oe)oallele conservation expected as well as the expected frequency distribution of number of hom(oe)oalleles and how this compares to what the authors observed in Fig S4.

Response: Sugarcane modern variates are interspecific polyploids and also tolerant to aneuploidy constitution, which makes the chromosome combination in each offspring unique and unpredictable [10,11]. Vieira et al [49] demonstrate that aneuploid gametes resulted from meiotic abnormalities, which included anaphase bridges and laggards, as well as asynchronous meiosis. This may be derived from the wild *S. spontaneum* ancestral (2n = 40–128), which evolved via polyploidy and aneuploidy.

Comprehensiveness vs Completeness

Reviewer: As a native English speaker “completeness” feels the most natural and simpler of the two words to use. Particularly, when quantitative measures are used to qualify the statements. e.g. by being able to identify 87.5% of CEGMA genes or 99.5% of Plantae lineage BUSCO genes within their assembly. However, if the authors insist on the use of the word “comprehensiveness” then please be consistent throughout the manuscript and fix occurrences of “completeness” on L147 and L467.

Response: We have accepted the reviewer’s suggestion in “diluting” down our genome completeness statement. None of the words are mentioned in the revised manuscript.

Conserved Synteny Analyses

Reviewer: Having re-read the sections regarding synteny of the SP80-3280 assembly with Sorghum and the authors responses, I am not convinced these analyses add anything substantial as the authors can only report

on the level of microsynteny due to most contigs containing only a small number of genes against which conserved synteny can be assessed. I would expect microsynteny to be very high and somewhat less interesting/important than more macrosynteny.

Response: We appreciate the reviewer's comment. However, we disagree with the argument that the analysis does not add anything substantial to our work for two reasons: (i) it proves, regardless of any expectation, that microsynteny between SP80-3280 and *S. bicolor* can be detected from our assembly and that it occurs at levels that are similar to those observed in other Saccharum genomes; (ii) as the referee acknowledges, the observation of expected levels of microsynteny suggests that there are no widespread artifacts in the assembly, an important remark if one wants to use this assembly as a reference for future analysis.

Reviewer: Reporting conserved synteny between a genome assembly and a close relative, for which conserved synteny is already assumed to be high (83% for R570 and Sorghum), is one way to provide confidence to the readers that the assembled contigs are accurate (e.g. are not chimeric).

Response: We agree with the reviewer.

Reviewer: However, since the SP80-3280 assembly is highly fragmented the authors can only really comment on the microsynteny involving a small number of genes owing to the fact that only 18% of contigs contain >1 gene per contig.

Response: We disagree and would like to reassure that our proportion of contigs with at least two markers is large enough to infer microsynteny. Sampling theory predicts that the minimum sample size required to estimate an expected proportion of 85% individuals sharing some trait in a population of size 430.000 with a 95% confidence level and a 5% error margin is 196 (Daniel WW, 2009 - ISBN: 978-1-118-30279-8, Chapter 6, 9th ed). The full set of contigs with >= 2 markers in the SP80-3280 assembly is 10.151, which is 500 times greater than the minimum number of contigs required to achieve the same levels of confidence. If we narrow down the error margin to 1% and increase the confidence level to 99% the minimum sample size required is 8.291. This number is still lower than the number of contigs we have used (10.151). Therefore, the number of contigs we have used is large enough to infer, with high level of confidence, the proportion of fully syntenic contigs, which is the measure we are using to access microsynteny conservation.
Additionally, since our markers are randomly spread through sorghum's genome (data not shown), we have no reason to believe that there could be any bias towards regions that deviate from typical levels of microsynteny in these genomes.

Formula to determine the sample size for estimating a proportion p:

$$n = \frac{Nz^2pq}{d^2(N-1) + z^2pq}$$

$$n = \frac{450609 * (1.96)^2 * 0.85 * 0.15}{(0.05)^2 * (450609 - 1) + \ (1.96)^2 * 0.85 * 0.15} = 196$$

$$n = \frac{450609 * (2.575)^2 * 0.85 * 0.15}{(0.01)^2 * (450609 - 1) + \ (2.575)^2 * 0.85 * 0.15} = 8298$$

n = sample size
N = Population size
p = proportion of a population sharing some characteristic
q = (1 - p)
z = value of the standard normal transformation, for choosing the confidence interval (1.96 for 95% confidence and 2.575 for 99%)
d = error, i.e. length of the interval around the estimated p, expressed as a percentage of p (0.01 or 0.05)

Reviewer: The example contig provided in Fig S10b (uti_cns_0054106) appears to contain 8 genes, which would appear to be more of an exception to the rule. Although, without having seen a distribution for the number of genes per contig it is difficult to say for sure.

Response: Indeed, this example is, to some extent, an exception, and we choose it only to demonstrate the ability of our algorithm to detect syntenic blocks. In addition, the number of contigs in our assembly with >= 2 genes is 79094 (17.6%). 10151 (2.3%) of these contigs have at least two marker genes and, within this subset, 3873 contigs (0.9%) have >= 4 genes. If we were to consider this latter subset of 3873 contigs as our sole sample of SP80-3280 contigs, we would still estimate the proportion of fully syntenic contigs, with 95% probability and an error no greater than 5%.

Open Science

Reviewer: While the authors state that resources (GigaDB, GitHub repositories, NCBI, GEO, and SUCESTFUN) will be made available upon publication, this does not abide by the "open science" principles of GigaScience as stated on GigaScience's editorial policies and reporting standards page (https://academic.oup.com/gigascience/pages/editorial_policies_and_reporting_standards). In particular, they place the same level of importance on such citable resources as traditional publications: "Making scientific datasets, protocols and code publicly available as early as possible before associated manuscripts are submitted is strongly recommended, particularly as we require reviewer access before the manuscript can be set out to peer review. These should be considered legitimate, citable products of research, and accorded the same importance in the scholarly record as citations of other research objects, such as publications. Therefore we follow the guidelines of the Data Citation and Software Citation Principles."
While I have access to the data made available through GigaDB, the same cannot be said for the other resources. If these resources cannot be made publicly accessible at this time, I kindly ask that I be added as a collaborator to your GitHub repositories (nathanhaigh) and create a suitable login for SUCEST-FUN. In addition, it would seem to make sense that a single canonical URL is provided for the data hosted at SUCEST-FUN rather than providing two URLs (L122-125, L502-505 and L761-762).

Response: We have provided now public access to GitHub. To access SUCEST-FUN genome browser framework at http://sucest-fun.org/cgi-bin/cane_regnet/gbrowse2/gbrowse/microsoft_genome_moleculo_scga7/ (only this URL is now provided in the manuscript), please use:
User: labuser
Password: s7c3stf7n

Recommendations for GigaDB Files

Reviewer: I make the following recommendations to ensure the published data follows standards expected by the community and is more easily reused, occupies the smallest space on disk and can be more quickly downloaded.

Response: We appreciate the reviewer's suggestions and declare that we have followed all recommendations.

Using All Sites for Phylogenetic Reconstruction

Response: We reconstructed the gene trees with "complete deletion" as suggested by the reviewer. We used both Maximum Likelihood and Neighbor-Joining methods and both presented similar tree topologies, specifically regarding gene family grouping. For PAL, complete deletion generated a tree with 333 positions in the final dataset (please see figure below). When we allowed only fewer than 5% of alignment gaps, missing data, and ambiguous bases at any position (partial deletion 95%), same topology is achieved, with significant increase on number of sites (1620 positions). Importantly, all trees result in the same topology as the one in Figure 4 (former Fig 3) of the manuscript. For SuSy, we had to exclude two partial sequences (SP80_0109792.1 and Sh 204B05 t000070) and rerun the alignment. The Maximum likelihood and Neighbor-Joining trees with complete deletion, as for PAL, have similar topology (see figure below) of the figure in the manuscript, with the same groups being formed composed of the same genes, however, with only 235 nucleotide positions analyzed. Again, when only fewer than 5% of alignment gaps, missing data, and ambiguous bases at any position were allowed (partial deletion 95%), the trees in both methods increased the number of sites analyzed and we still have the tree structure. In conclusion, besides some differences in branch lengths and relationship among different groups, all trees showed the same topology considering the gene family groups, that is, gene clades are the same for all analyses, including the analysis presented in the manuscript using "all sites", thus supporting that the analysis is coherent. Our idea to use gene trees in the manuscript is to present the breadth of the genome information made available and not to resolve the precise evolutionary history of each individual gene. As a result, we decided to keep the original figure in the manuscript for PAL, and replace the SySy tree with the new one after removal of two partial sequences.

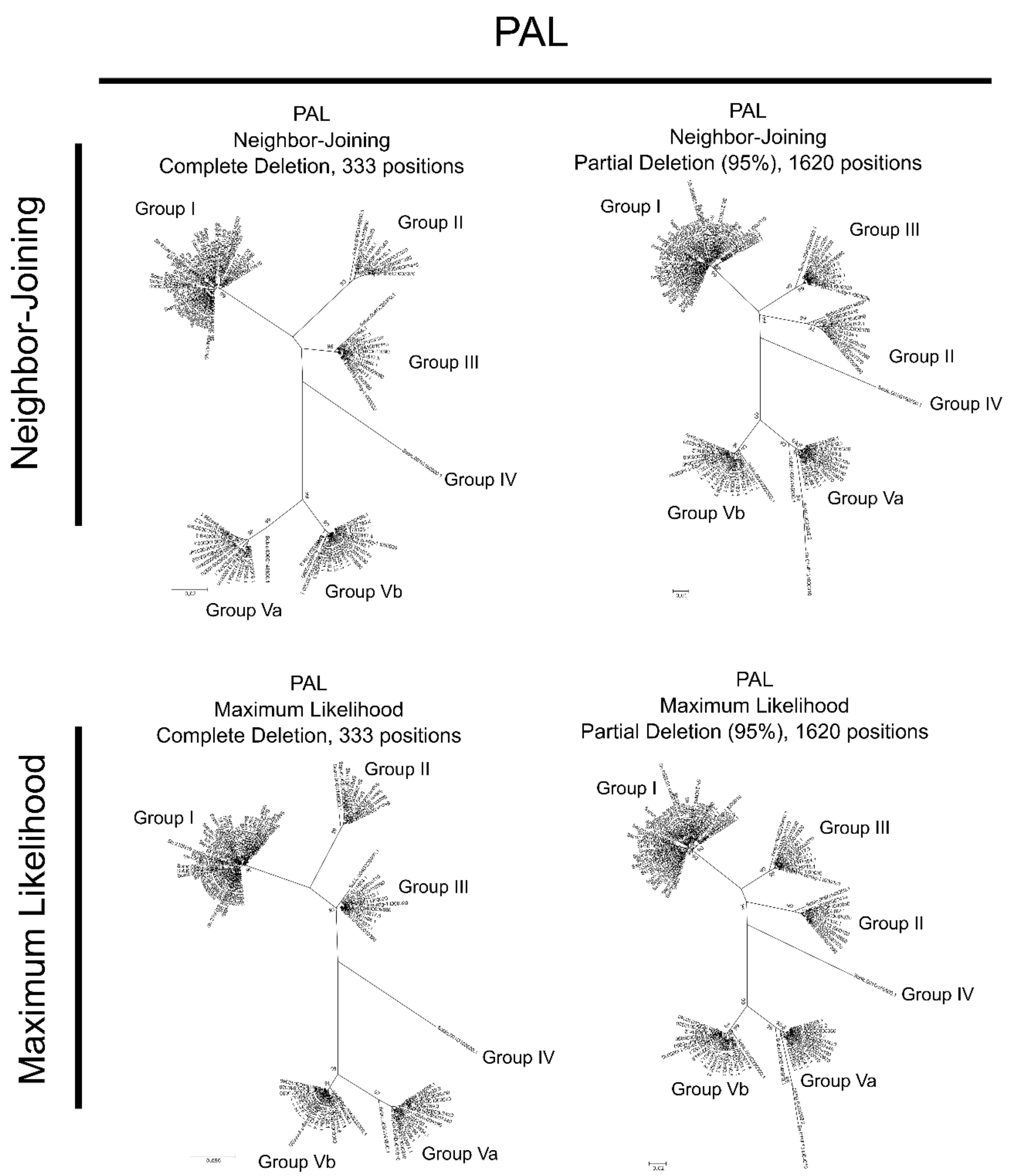
PAL
Neighbor-Joining
Maximum Likelihood
PAL
Neighbor-Joining
Complete Deletion, 333 positions
Group I
Group II
Group III
Group IV
Group Va
Group Vb
PAL
Neighbor-Joining
Partial Deletion (95%), 1620 positions
Group I
Group III
Group II
Group IV
Group Vb
Group Va
PAL
Maximum Likelihood
Complete Deletion, 333 positions
Group I
Group II
Group III
Group IV
Group Vb
Group Va
PAL
Maximum Likelihood
Partial Deletion (95%), 1620 positions
Group I
Group III
Group II
Group IV
Group Vb
Group Va

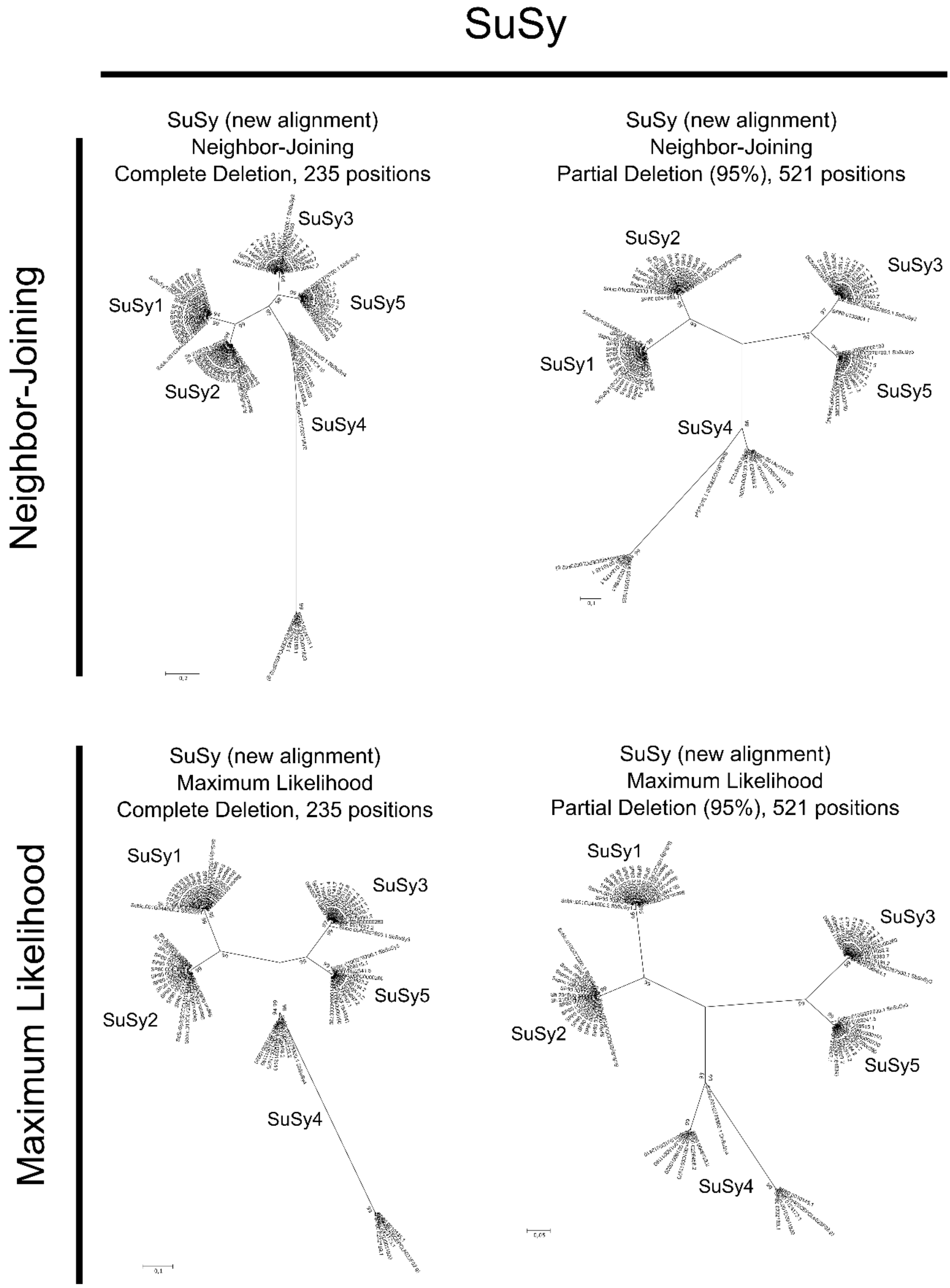

SLR Methods Detail

Reviewer: While I appreciate the authors response states "the number of fragments in each well is relatively low" this statement is not quantitative. I do not know if the standard SLR library prep protocols were designed with much smaller genomes in mind and have a standard dilution series or if the protocol is general purpose enough to have a "target" number of fragments per well irrespective of the genome size. Elsewhere, I have

seen reference to a range of fragments per well from a few thousand to many thousands. I would like top see more quantitative information about the dilution performed and the expected number of fragments per well. I think it is important to understand the expected number of fragments within each well as it impacts on the probability of obtaining chimeras due to fragments from homeoloci (or other high sequence identity loci) ending up in the
same well.
A related issue is the lack of clarity around whether the dilution was done per 384 well plate and this was then replicated 26 times to generate the specified “26 TruSeq Synthetic Long-Read DNA libraries” or if the dilutions were done across all 26 x 384 well plate.

Response: We have contacted Illumina and they stated that the SLR library prep protocol (dilution in the 384 well plate) is irrespective of genome size. Genome size dictates only how many libraries are required. For the Long Read protocol, the intent is to get 3fg of PCR products per well. The PCR products are supposed to be 8-10kb long and each diluted well of the 384 well plate should contain ~325 fragments of 8-10kb on average. In addition, the analysis software deals with “collisions”, which are overlapping genomic fragments or fragments containing homologous regions, by throwing out fragments containing inconsistent bases at a higher rate than expected from sequencing error rate. For instance, an assembled fragment that had 40X coverage at a particular nucleotide, the total of 38 adenosine basecalls and 2 guanine basecalls, would be kept and the quality score at that nucleotide would be adjusted downward to reflect the mismatching basecalls. If that same nucleotide instead had 15 adenosine basecalls and 25 guanine basecalls, the fragment would be thrown out because it is likely to represent either overlapping fragments, or a PCR error in the initial amplification. So, there is no need to estimate a rate of chimeras based on genome size because the software should remove them regardless of genome size.
Regarding the dilution step, each dilution was done per 384 well plate and this was then replicated 26 times to generate the specified “26 TruSeq Synthetic Long-Read DNA libraries”. Finally, we have included such information in the material and methods section, as follows:

L414-423: “Genomic DNA was sheared into 5-10 kb fragments and diluted in a 384-well plate. DNA fragments were ligated with PCR primers and specific sequences, which identify the 5’ and 3’ ends. The fragments from each well were amplified, fragmented and barcoded with unique indices, to create a TruSeq Synthetic Long-Read DNA library. In total, 26 libraries were made. The short fragments created in the second step of fragmentation were pooled and sequenced on the HiSeq instrument at the Illumina Service Genome Network. The reads from each of the 384 wells were pre-processed to correct sequencing and PCR errors. Contigs were produced from the paired-end information and further scaffolded together to resolve repeats and fill in gaps. In this step, the software removes fragments containing inconsistent bases at a higher rate than expected from sequencing error rate. More details on the informatics pipeline for short read scaffolding into long reads are available in the Fast Track Services Long Reads Pipeline User Guide [59].”

Minor Comments

Reviewer: L48 - “Their alignment to single copy genes” implies that a sequence similarity search was performed where the single copy genes were being searched using sugarcane sequences as query. However, I believe the authors performed the opposite.

Response: We thank the reviewer for pointing this out. Indeed, we aligned the sugarcane sequences as queries to the single copy genes. By doing the opposite, the presence of multiple sugarcane gene copies would result in multiple alignments in the vast majority of cases, which could in turn lead to errors in the association of genes from both databases. We have changed the text as follows:

L48: "The alignment of single-copy genes in diploid grasses to the putative genes, indicates that …"

Reviewer: L59, L116 reword to avoid "resolved" as this implies all homeologs have been assembled and a present in the assembly.

Response: In L59, we have changed the sentence for "This assembly represents …". In L115, we have changed the sentence for "For a large fraction of the gene space, an average of 6 sugarcane haplotypes, putatively homo(eo)logs, were identified."

Reviewer: L152 - "up to 15 matches" seems to be inconsistent with the "17 matches" stated in the caption of Fig. S4.

Response: We thank the reviewer for pointing this out and corrected the sentence in Figure 1 caption (previous Fig. S4) as follows:
L1062-1064:"For 127,940 aligned ESTs, 106,133 (84.9%) show 2 up to 30 matches on the genome (A), while for CEGMA regions, 205 (87.2%) range from 2 to 17 matches on the genome (B). SPALN v 2.3.3 [67] was used for alignment."

Reviewer: L428 - "we transformed the quality scores" does not provide any information on how the transformation was performed. e.g. Did the authors simply threshold the quality values to Q40 to Q values > 40 were set to Q40? Did they perform a linear transformation/scaling so the highest Q value became Q40? Something else?

Response: We simply threshold the quality values over Q40 were set to Q40. This does not hurt any CA performance or assembly results since CA did not use quality values to overlap reads. To clarify this issue, we have changed the text as follows:
L429-431: "Since synthetic long reads are very accurate and some of the base qualities exceeded this upper bound, we set the quality scores over Q40 as Q40 to allow them to be appropriately parsed."

Reviewer: L482-483 – The mean length of contigs with good alignments to the publicly available chloroplast/mitochondrial genomes is only 4kb. Can the authors explain why these genomes are so heavily fragmented in their assembly given 1) their higher coverage (>20x) compared to the contigs derived from the nuclear chromosomes and 2) Given the mean SLR length is 4.9kb.

Response: The comparison to mitochondrial and chloroplast genomes was performed after long-read assembly. The fragmented nature of our assembly may be related to nuclear genome complexity and the assembler's difficulty in dealing with polyploidy. We have tried to reassembly both plastid genomes using only the subset of contigs. However, we still get a fragmented assembly, probably due to low sequence input.

Reviewer: L489-490 – Excessive precision on percentages; restrict to 2 decimal places. In addition, swap commas for decimal points.

Response: We apologize for this we have changed the text as follows:
L493: “aligned against the chloroplast genome presented 99.99% and 99.99% of coverage and identity respectively”.
L496-497: “The alignment against mitochondrial chromosomes 1 and 2 presented 99.85% and 99.93% of coverage and 99.90% and 99.94% of identity, respectively”.

Reviewer: L500 – Please specify version of SPALN used.

Response: We apologize for this we have changed the text as follows:
L507: “… contigs sequences using SPALN v 2.3.3 [67] applying …”

Reviewer: L558-564 – I still find this paragraph a little confusing so rewording might be useful. Am I correct in thinking that the upstream regions of homeologs were being analysed and that this analysis was done per homeolog cluster? That the analysis consisted of aligning and then calculating a distance matrix for the upstream region of each homeolog cluster. That this was done by defining the upstream region as either 100, 500 or 1000 bp. If so, it is unclear if the authors have presented information as to the size distribution of these clusters and how the cluster size might affect the distance calculation used for each data-point in Fig1B.

Response: The understanding of the reviewer is correct - upstream regions of each homeolog cluster were analyzed in a pairwise fashion, resulting in a distance matrix for each cluster. We did this separately for three different sequence lengths. The size of the clusters is that shown in Figure 2A (former Fig 1) and we have amended the text to make this clear. Because we calculated pairwise alignments between upstream regions, gene clusters with more copies naturally contributed with more data points in Figure 2B.
L567-572: “Finally, for each distance range, we parsed the alignments and computed the dissimilarity level considering both mismatches and gaps to obtain a distance matrix for the upstream region of each cluster. To avoid partial alignments of the upstream sequences, only alignments up to 20% shorter or longer than the expected sequence length were considered. Note that the dimension of the distance matrix varied between gene clusters, according to the distribution of cluster sizes shown in Fig 2A.”

Reviewer: L164-165 (Fig 2 caption) – Mentions Ion PGM data. This is the only mention of Ion PGM data, is this the same data when “RNA-Seq data” is mentioned in the manuscript (L181, L531, L575, L584, L591, L611, L760, L1078 and L1079)? If so, this needs clarifying since RNA-Seq is now pretty synonymous with Illumina.

Response: The understanding of the reviewer is correct. We have added this information to the first mention in the manuscript, as follows:
L179-180: “RNA-Seq data from leaves and internodes of SP80-3280 (Ion PGM Sequencing) [28] shows expression …”

Reviewer: Fig 2 – Why has the frequency range of Fig2A and 2B changed from approx 160 and 200 respectively in the original submission to approx 80 and 100 respectively in the latest revision? Please also include information in the caption as to how the colour scale is derived.

Response: We have accepted the reviwer’s previous suggestion and have the provided a new figure: colour (heat) were scaled as a percentage of the number of genes with a given total number of homeologous. We now have changed the figure caption as follows:
L1075-1078: “**Fig. 3 – Homo(eo)log expression**: The percentage frequency of sugarcane genes plotted against the total number of homo(eo)logs per gene and the number of expressed homo(eo)logs per gene. Genes

with cDNAs aligned with FPKM > 1 were considered expressed. Plots show sense (**A**) and antisense (**B**) transcripts. Reads from Ion PGM Sequencing were used and strand orientation is maintained [28]."

Reviewer: Fig S4 – Changed "Frequency density" to "frequency histogram". Include some info about the use of SPALN to perform the alignments.

Response: We have changed the text as follows:
L1061-1064: "**Fig. 1 – Frequency histogram of Expressed Sequence Tags (ESTs) and Core Eukaryotic Genes Mapping Approach (CEGMA) regions alignment on Sugarcane genome assembly.** For 127,940 aligned ESTs, 106,133 (84.9%) show 2 up to 30 matches on the genome (**A**), while for CEGMA regions, 205 (87.2%) range from 2 to 17 matches on the genome (**B**). SPALN v 2.3.3 [67] was used for alignment."

Reviewer: Fig S11 – Please provide information regarding the choice of the outgroup RGA2-blb, particularly since it is so distant to the I2C-2 ingroup sequences.

Response: RGA2-blb is the reference gene of I2C-2 class and has been used by Rossi et al (2003) [DOI 10.1007/s00438-003-0849-8] to recover the sugarcane ESTs used as probes for BAC selection.

Reviewer: Where e-value thresholds have been specified, the powers would look better as superscripts. e.g. rather than 1x10-15 use $1x10^{-15}$.

Response: We have changed the text as follows:
L486: "… selected based on cutoff E-value ≤ $1x10^{-15}$"
L530: "… BLASTp (v2.2.30+, -*evalue* $1x10^{-5}$)."
L550: "… using the BLASTn (v2.2.30+, -*evalue* $1x10^{-6}$)."
L623: "… using tBLASTn (v2.2.30+, -*evalue* $1x10^{-6}$)."
L630: "… with e-value smaller than $1x10^{-3}$ were kept."
L700: "… from BLAST searches, with e-value <= 10^{-5},"
L733: "… with BLASTp considering an e-value threshold of $1x10^{-5}$"

Include Detail from Previous Responses into Manuscript

Reviewer: The details included in the author's previous responses, pasted below, should be included in the MS as they would also be beneficial to readers:

Response: We accept the reviewer's suggestion and have included the sentence as follows:
L461-463: "For any CDS with multiple HSPs (High-scoring Segment Pair) against the same contig that passed the filtering criteria, we used the union of such hits, excluding any potential overlap. Given that most contigs contained only one or two genes, we expect very little influence of spurious hits to different gene regions."
