# Supplementary material for: Assembly of the 373k gene space of the polyploid sugarcane genome reveals reservoirs of functional diversity in the world's leading biomass crop
Source: Gigascience. 2019 Nov 29;8(12):giz129. doi: 10.1093/gigascience/giz129 (PMC6884061; doi:10.1093/gigascience/giz129)
Supplement: giz129_Supplemental_Figures_and_Tables [file giz129_supplemental_figures_and_tables.zip › Second revision GIGA-D-19-00013 Additional file 1.docx]

**Additional file 1**

**Supplemental Figures S1 to S11**

Assembly of the 373K gene space of the polyploid sugarcane genome reveals reservoirs of functional diversity in the world’s leading biomass crop

Souza, GM1*; Van Sluys, MA2*; Lembke, CG1; Lee, H3,4; Margarido, GRA5; Hotta, CT1; Gaiarsa, JW2; Diniz, AL1; Oliveira, MM1; Ferreira, SS1,2; Nishiyama-Jr, MY1,6; ten-Caten, F1; Ragagnin, GT2; Andrade, PM1; Souza, RF7; Nicastro, GG7; Pandya, R8; Kim, C9,10; Guo, H9; Durham, AM11; Carneiro, MS12; Zhang, J13; Zhang, X13; Zhang, Q13; Ming, R13,14; Schatz, MC3,15; Davidson, B8; Paterson, A9; Heckerman, D8.

*These authors contributed equally to this work and are co-corresponding authors: glmsouza@iq.usp.br and mavsluys@usp.br


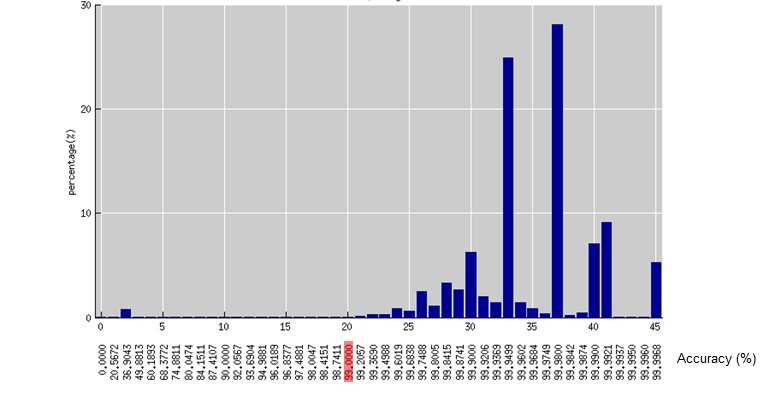


**Fig S1: Illumina long-reads base quality distribution.** The x-axis represents Phred quality score and accuracy converted to percentage scale (from 0.00 to 100.00%). More than 99% of bases have >99% accuracy.


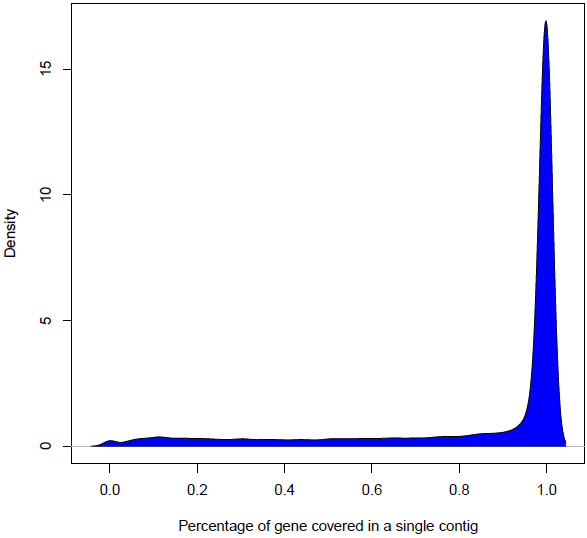


**Fig S2: Distribution of the largest fraction of each sorghum gene contained in a single sugarcane unitig.** Sorghum coding sequences were aligned with BLASTn to the assembled sugarcane contigs and their coverage computed. Only the match with the largest coverage was considered for genes with multiple matches. The density plot represents the frequency of the coverage percentage. Among 39,207 annotated sorghum CDS, 71.1% displayed coverage greater than 90% in a single sugarcane contig, and the median CDS coverage was 99.7%.


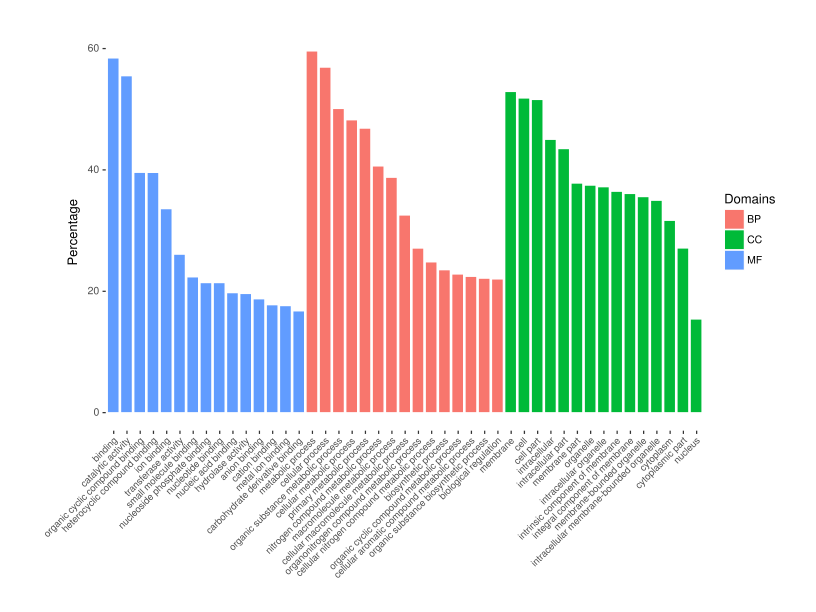
**Fig S3: GO classification of predicted genes**. Proteins sequence from predicted CDS were aligned to UniRef50 [29] and RefSeq non-redundant proteins databases and mapped to GO terms using the Blast2GO framework. 195,651 sugarcane proteins were mapped to 10,362 different GO terms with E-value < 1e-5. In the bar chart, we show the 15 higher level categories more abundant for each domain.


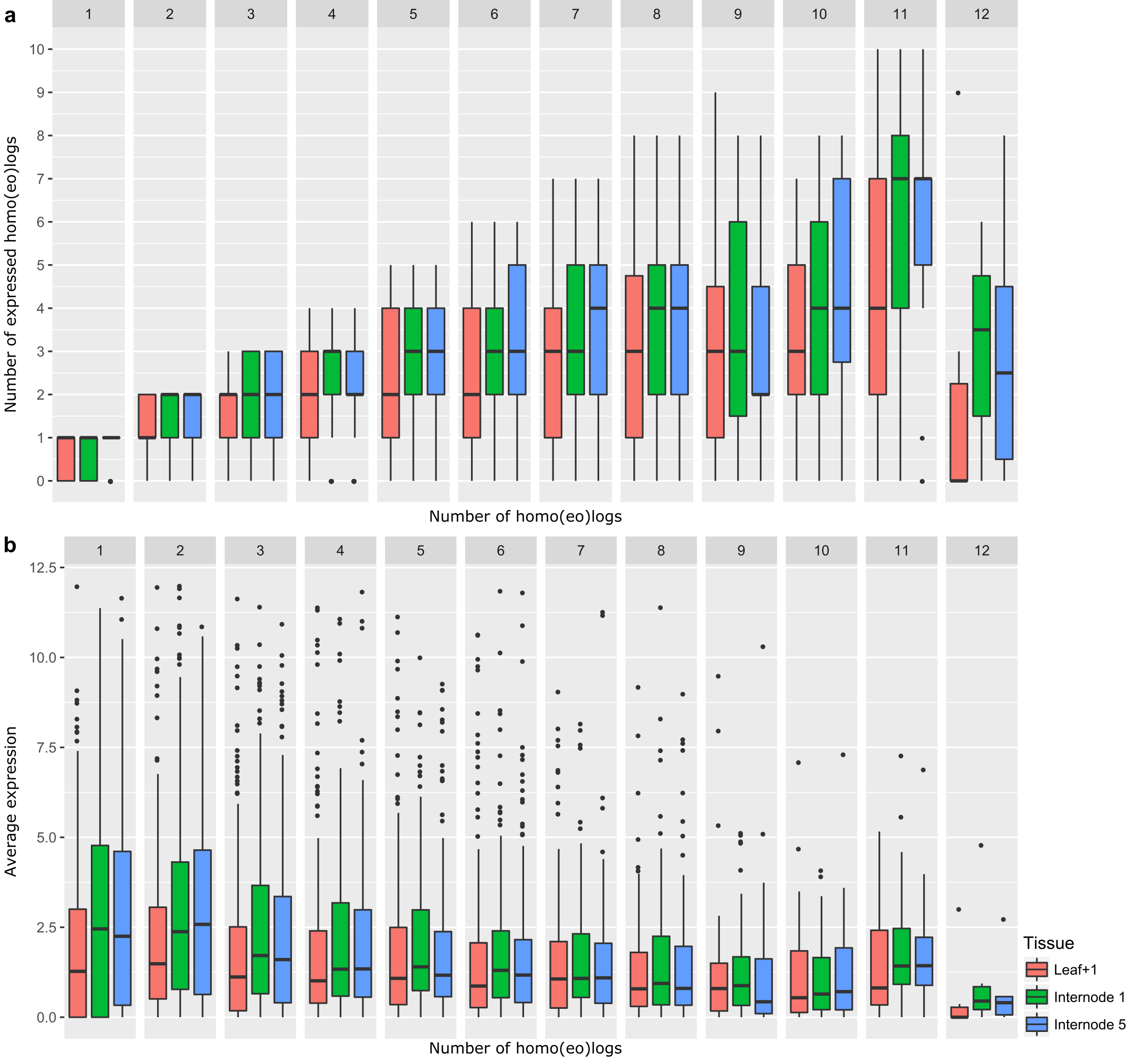


**Fig S4:** **Putative homo(eo)logs expression:** (a) number of expressed homo(eo)logs and (b) average expression of homo(eo)logs by tissue.


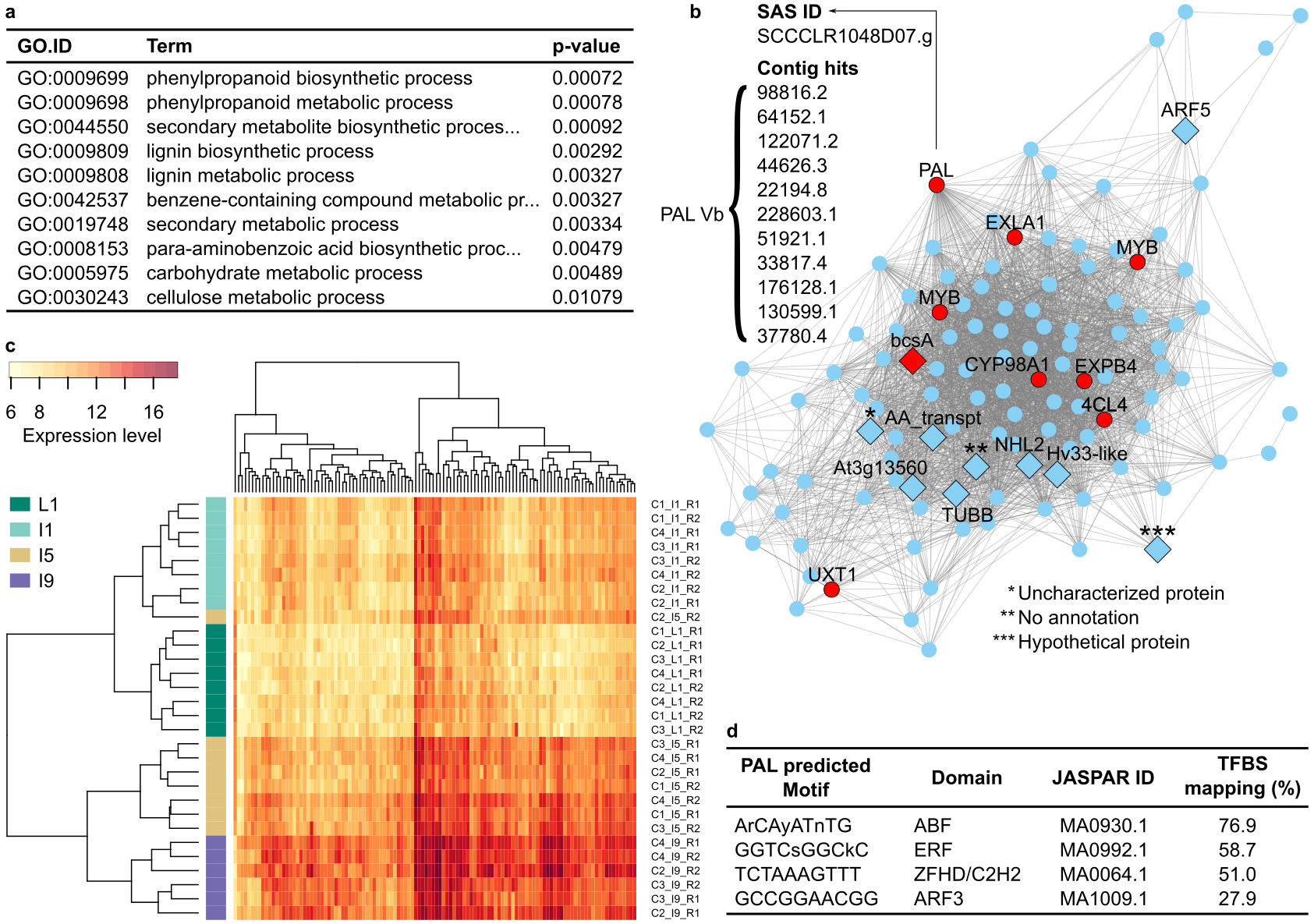


**Fig S5 – Co-expression analysis:** The SP80-3280 transcriptome data was generated using the CaneRegNet oligoarray platform [97]. RNA was extracted from +1 leaf (L1) and immature (I1), intermediate (I5) and mature (I9) internodes, collected at 4 (C1), 8 (C2), 11 (C3) and 13 (C4) months after field planting. Out of 17,476 significantly expressed genes, 1,129 were clustered in 7 co-expression modules. a) Gene ontology enrichment analysis showing that co-expression module M4 (116 SAS) include genes involved in phenylpropanoid metabolism. b) M4 network with top nodes (diamonds) and already known genes involved in lignin biosynthesis (red highlighted). c) M4 gene expression heat map suggests a tissue-specific pattern. d) Out of 116 co-expressed SAS, 104 overlap genes in our assembly which have an upstream region at least 1,500 bp long. Mapping of TFBS, predicted by a de novo approach within upstream region of SP80-3280 PAL genes, suggests that ABF, ERF, ZF-HD/C2H2, and ARF3 may act in the regulation of M4 genes expression.

**Fig S6: Comparative TE genome contribution to gene-space and chromosome level assembly:** Transposable element family counts are represented as log 10 scale to enable comparative analysis. In house dataset composed of manually annotated transposable elements as described previously [44, 97] were mapped in the three genomes assemblies using BLASTn and a 90:90 identity:coverage cut off.

**Fig S7:** Distribution of the number of deleterious variations (1,334) and single copy genes (585) containing such variations based on the alignment of sugarcane (SP80-3280) contigs to the genic regions of sorghum chromosomes.


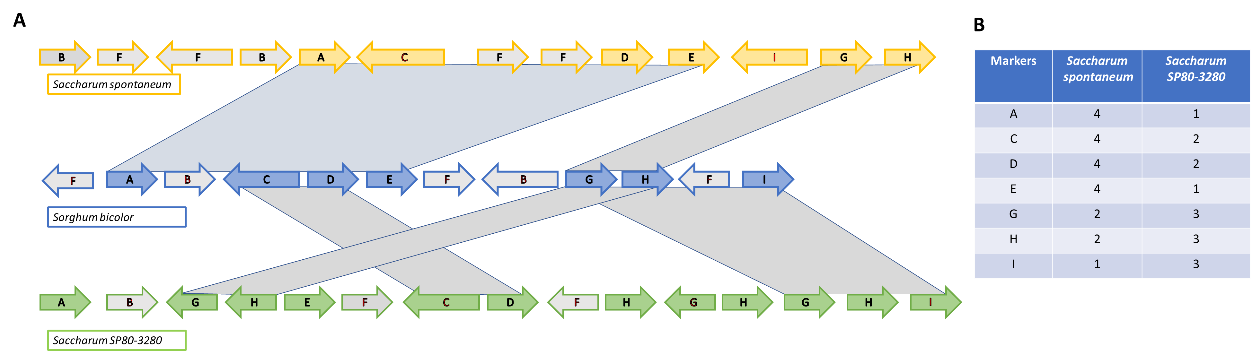


**Fig** **S8:** **Syntenic block assignment.** To identify syntenic blocks a given region in the reference chromosome, containing at least two selected synteny markers, must share homology with a region in the target sequence and homologous marker genes in these regions must be present in the same order relative to one another and be encoded by the same DNA strand. Only genes present as single copies in the reference genome (S. bicolor), and their orthologs in the target genomes (S. spontaneum and SP80-3280), were used as synteny markers. Genes in the reference genome, Sorghum bicolor, are represented as blue arrows, and the two targets genomes are represented by yellow (S. spontaneum) and green (SP80-3280) arrows. The arrows filled with light gray are genes that are duplicated in the reference genome and, therefore, were not used as synteny markers. The gray boxes highlight examples of homologous regions that are considered syntenic blocks, according to our criteria.


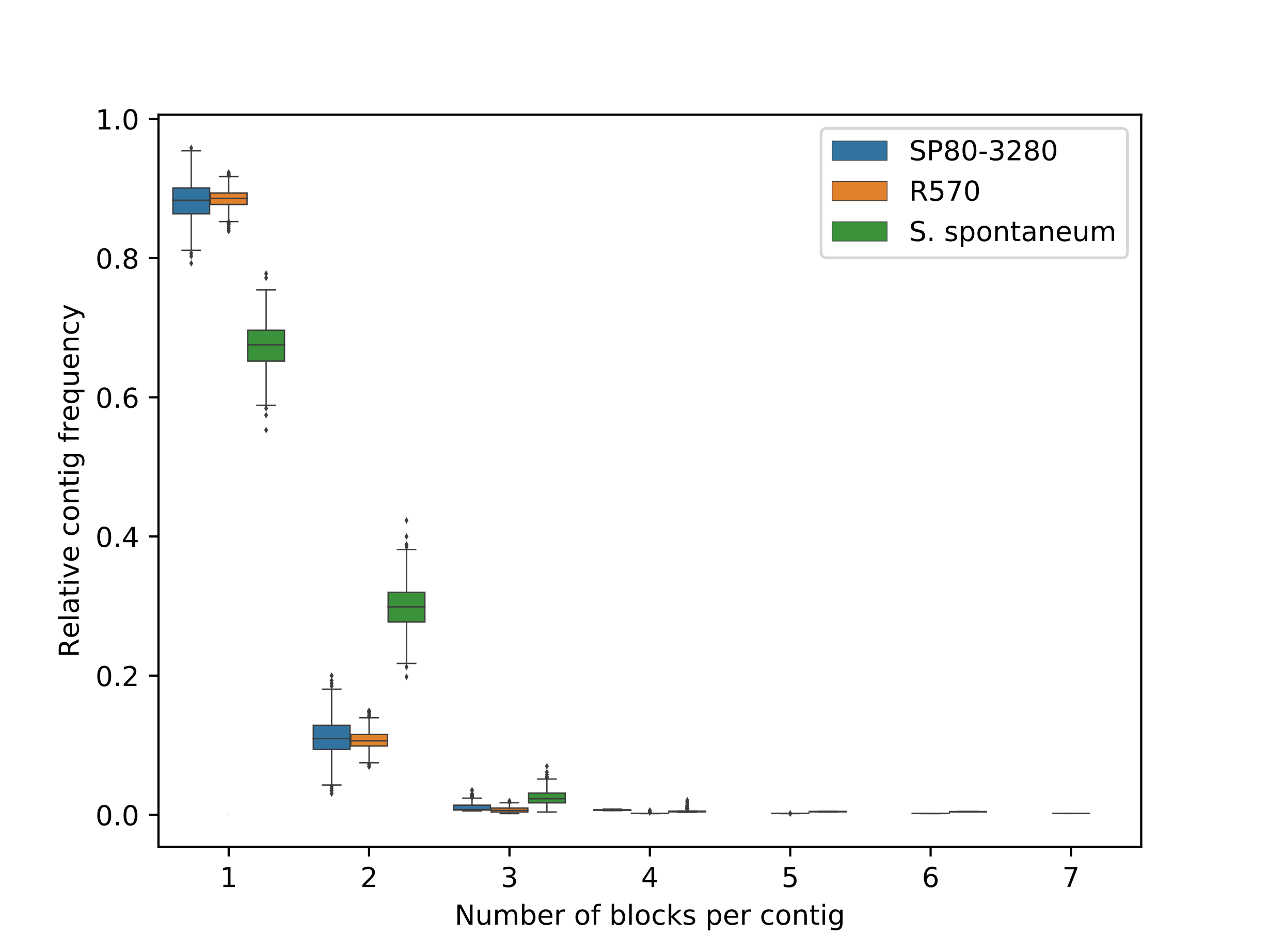


**a**

| Syntenic blocks | Contigs |
| --- | --- |
| 1 | 7906 |
| 2 | 1298 |
| 3 | 90 |
| 4 | 8 |
| 5 | 1 |

**b**

**c**

**b**

**c**

**
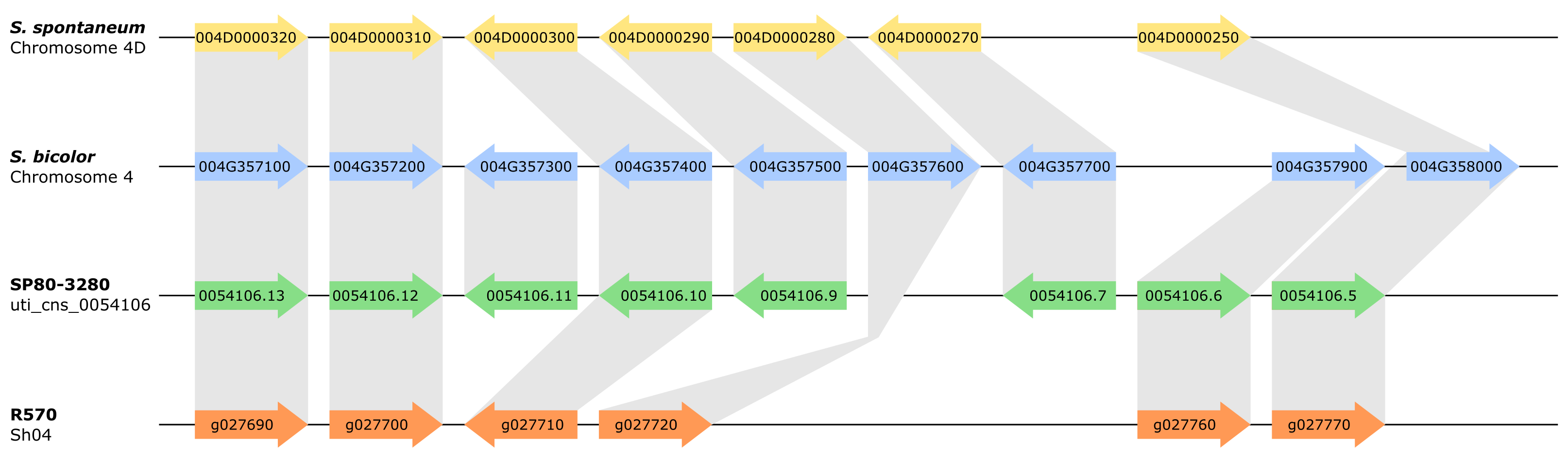
**

**Fig S9:** **Conservation of gene order.** **(a)** Absolute frequency of SP80-3280 contigs versus Sorghum showing the number of syntenic blocks per contig. A syntenic block is a segment of a contig where all genes are present in the same order and orientation as their orthologs in *Sorghum bicolor*. **(b)** An example of a highly colinear region; arrows represent transcription direction. **(c)** Frequency of contigs versus the number of syntenic blocks. Subsets of the contigs of SP80-3280 and fragments of chromosomes from *Saccharum* R570 and *S. spontaneum* were independently sampled according to the abundance and number of genes observed for the contigs from SP80-3280.


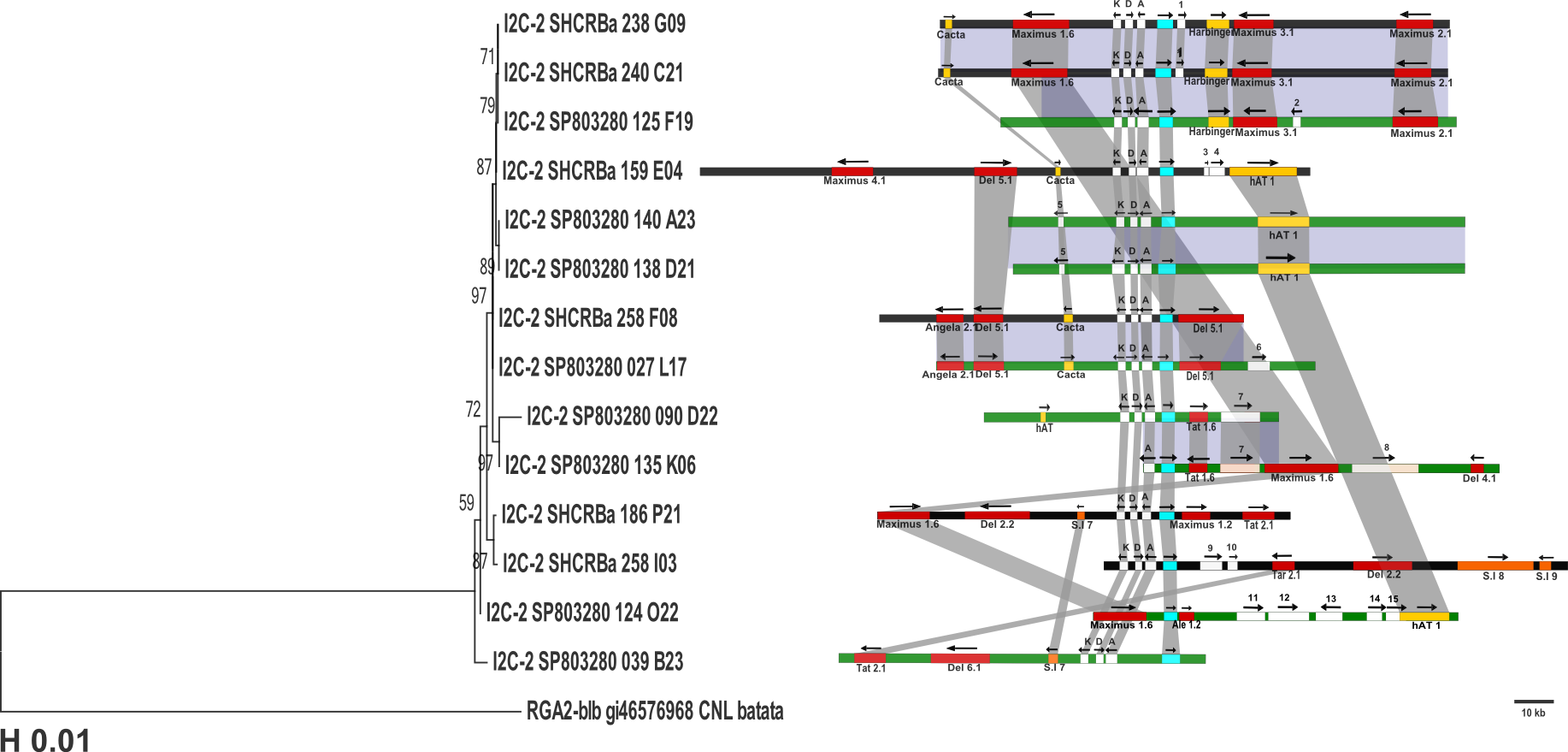


**Fig S10:** **Comparative genomics of *I2C-2* locus**. On the left, is represented an I2C-2 protein tree built with MEGA® using Neighbour-Joining and JTT model with 500 replicates. Black horizontal lines represent R570 genomic BACs while green represents SP80-3280. Shaded areas represent nucleotide similarities across sequenced BAC clones. Colored rectangles within genomic regions represent Genes and Transposable Elements. The central blue gene represents the I2C-2 gene used to select the BAC clones as described in de Setta et al [63]. Three white boxes named K, D, A represent clustered genes found in all clones (K: kinase; D: Dog1; A: Aminotransferase). Red represents Class 1 TE and Yellow Class 2 TE. Arrows represent transcription direction.


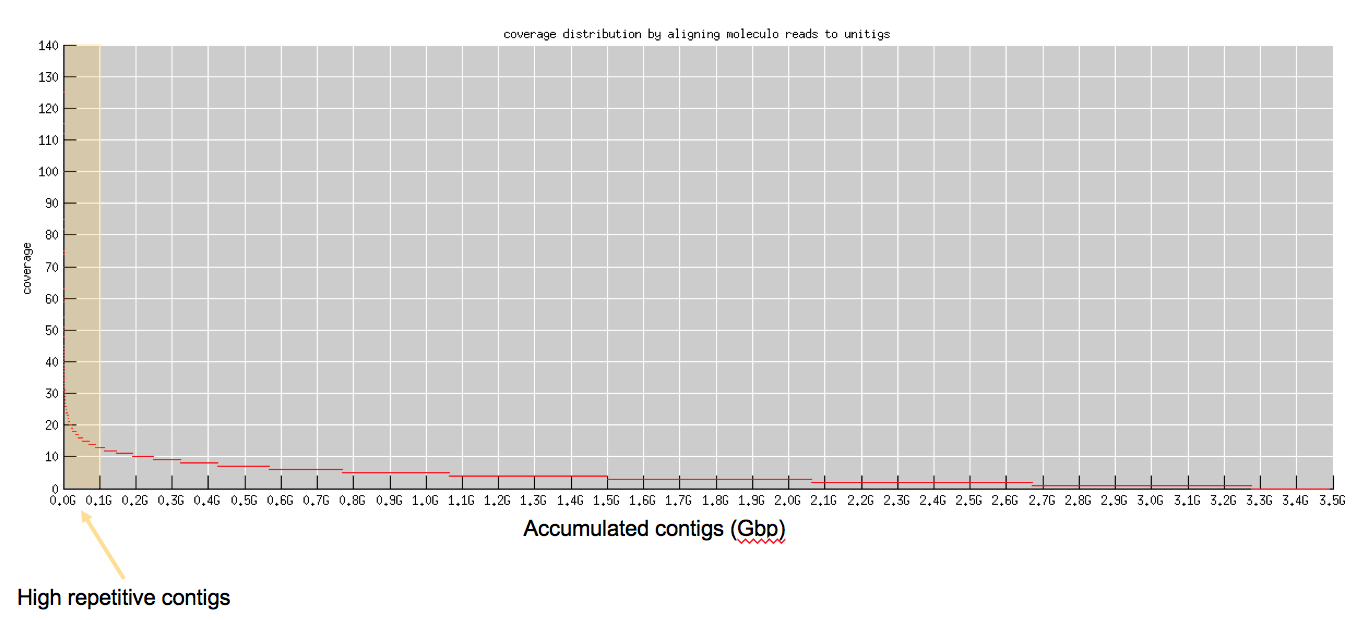


**Fig S11 – Synthetic long read coverage plot:** The reads were mapped back to the contigs. After sorting contigs from highest coverage to lowest, only 0.1 Gbp of contigs had very high coverage which represents highly repetitive sequences.
