# Supplementary material for: Assembly of the 373k gene space of the polyploid sugarcane genome reveals reservoirs of functional diversity in the world's leading biomass crop
Source: Gigascience. 2019 Nov 29;8(12):giz129. doi: 10.1093/gigascience/giz129 (PMC6884061; doi:10.1093/gigascience/giz129)
Supplement: giz129_Response_to_Reviewer_Comments_Original_Submission [file giz129_response_to_reviewer_comments_original_submission.pdf]

Author's Response To Reviewer Comments

Close

Dear Dr. Hans Zauner

Firstly, we would like to thank the editor and reviewers for the valuable comments on our submitted manuscript. We have responded to all comments and criticisms and have revised the paper in light of them. A point-by-point response to these concerns is provided in the 'Response letter GIGA-D-19-00013.docx' file. We reinforce the high value of the presented sugarcane hybrid gene space assembly, not only for the sugarcane community, but also for those interested in unraveling genomics of complex polyploid crops. The revised version of our manuscript (in addition to Fig.2 and Additional files 1 and 2) has been uploaded.

Close
