# Supplementary material for: Assembly of the 373k gene space of the polyploid sugarcane genome reveals reservoirs of functional diversity in the world's leading biomass crop
Source: Gigascience. 2019 Nov 29;8(12):giz129. doi: 10.1093/gigascience/giz129 (PMC6884061; doi:10.1093/gigascience/giz129)
Supplement: giz129_Response_to_Reviewer_Comments_Revision_1 [file giz129_response_to_reviewer_comments_revision_1.pdf]

Author's Response To Reviewer Comments

Close

We thank the editor and the reviewer. We declare that we have responded to all suggestions. A point-by-point response to each comment is presented. The revised version of our manuscript, in addition to a new Fig 1 (former Fig S.4), Fig.4 (former Fig.3) and Additional file 1, as well as all revised files (as suggested by the reviewer) have been uploaded.

Close
