# Supplementary material for: Assembly of the 373k gene space of the polyploid sugarcane genome reveals reservoirs of functional diversity in the world's leading biomass crop
Source: Gigascience. 2019 Nov 29;8(12):giz129. doi: 10.1093/gigascience/giz129 (PMC6884061; doi:10.1093/gigascience/giz129)
Supplement: giz129_Response_to_Reviewer_Comments_Revision_2 [file giz129_response_to_reviewer_comments_revision_2.pdf]

Author's Response To Reviewer Comments

Close

We have uploaded our final version of the manuscript with data citation and updated funding information.

Close
