# Supplementary material for: Assembly of the 373k gene space of the polyploid sugarcane genome reveals reservoirs of functional diversity in the world's leading biomass crop
Source: Gigascience. 2019 Nov 29;8(12):giz129. doi: 10.1093/gigascience/giz129 (PMC6884061; doi:10.1093/gigascience/giz129)
Supplement: giz129_Reviewer_1_Report_Original_Submission — Nils Stein -- 3/10/2019 Reviewed [file giz129_reviewer_1_report_original_submission.pdf]

**Reviewer Report**

**Title: Assembly of the 373K gene space of the polyploid sugarcane genome reveals reservoirs of functional diversity in the world's leading biomass crop**

**Version: Original Submission** **Date:** 3/10/2019

**Reviewer name: Nils Stein**

**Reviewer Comments to Author:**

Souza et al. report a gene-space assembly of the polyploid sugarcane genome. They tackled the difficulty of assembling a highly redundant gene-space by relying on the Illumina Long Read technology and could report an impressive gene count of over 370,000 gene models, which can be expected for a genome that is expected to contain between 8 and 13 sets of chromosomes.
The gene-space assembly will provide an important resource for establishing more functional genomic studies in sugarcane, eventually leading also to new approaches of sugarcane crop improvement through genomics-based crop improvement, most likely, however, through transgenic approaches including gene editing.
The manuscript needs to be corrected for typos: homo(eo)logs genes and homo(eo)logs alleles is a recurring mistake - it should either read homo(eo)logs or homo(eo)logous genes / alleles.
The paper reports a sequence resource which is used for sequence analysis and interpretation of a few gene families - while this is of interest in respect of the economic use of the crop, the analysis remains speculative and descriptive thus could be presented in a more concise way. motif analysis results and SNV density are reported only or again in the discussion. I highly recommend to shorten the manuscript by merging results and discussion or shorten significantly the discussion and conclusion section, which will increase the readability of the manuscript.
I would have appreciated a stronger attempt of using independent evidence for the redundancy of the gene set. Especially in regard of the promoter analysis it would be more intuitive to see an assessment of sequence quality in the 5`and 3`regions. This might be hidden in the supplemental material, which I could not access. It remains elusive to me if the authors have assessed their assembly by a read coverage analysis in order to identify problematic regions in the contigs/unitigs.
The authors report the use of custom scripts in their analyses. These must be submitted to a public repository, e.g. Github or others.
Table 1 only reports 454 data for BAC clones while the methods mention also PACbio data - please comment.
To my opinion section "Sugarcane and sorghum polymorphisms support recent allotetraploidy" and Figure 4 require more explanation. Neither the paragraph nor the figure provide any conclusion that would justify the title of this section. I assume the authors interprete the predominant occurance of biallelic SNPs as an indication of allotatraploidy? but is this really unexpected to have a predominance of biallelic SNPs anyway? It is also unclear what is the context of the 4750 SNP between sorghum and sugarcane. Only the SuSy genes or all 30,000 / 300,000 sorghum/sugarcane homologs? Again I couldn`t access the supplements for this section, however, I recommend to present this part in a more tangible

manner.
BUSCO analysis asks for the presence of 1400 single copy genes highly conserved among higher organisms. In theory each of the 8-13 sets of chromosomes is expected to carry a full set of BUSCO genes, at least shortly after the polyploidisation event. so, how conclusive is a report of "5.4% of conserved genes could not be identified" in this context?

**Methods**

Are the methods appropriate to the aims of the study, are they well described, and are necessary controls included? Choose an item.

**Conclusions**

Are the conclusions adequately supported by the data shown? Choose an item.

**Reporting Standards**

Does the manuscript adhere to the journal's guidelines on minimum standards of reporting? Choose an item.

Choose an item.

**Statistics**

Are you able to assess all statistics in the manuscript, including the appropriateness of statistical tests used? Choose an item.

**Quality of Written English**

Please indicate the quality of language in the manuscript: Choose an item.

**Declaration of Competing Interests**

Please complete a declaration of competing interests, considering the following questions:

- Have you in the past five years received reimbursements, fees, funding, or salary from an organisation that may in any way gain or lose financially from the publication of this manuscript, either now or in the future?
- Do you hold any stocks or shares in an organisation that may in any way gain or lose financially from the publication of this manuscript, either now or in the future?
- Do you hold or are you currently applying for any patents relating to the content of the manuscript?
- Have you received reimbursements, fees, funding, or salary from an organization that holds or has applied for patents relating to the content of the manuscript?

- Do you have any other financial competing interests?
- Do you have any non-financial competing interests in relation to this paper?

If you can answer no to all of the above, write 'I declare that I have no competing interests' below. If your reply is yes to any, please give details below.

I declare that I have no competing interests

I agree to the open peer review policy of the journal. I understand that my name will be included on my report to the authors and, if the manuscript is accepted for publication, my named report including any attachments I upload will be posted on the website along with the authors' responses. I agree for my report to be made available under an Open Access Creative Commons CC-BY license (http://creativecommons.org/licenses/by/4.0/). I understand that any comments which I do not wish to be included in my named report can be included as confidential comments to the editors, which will not be published.

Choose an item.

To further support our reviewers, we have joined with Publons, where you can gain additional credit to further highlight your hard work (see: https://publons.com/journal/530/gigascience). On publication of this paper, your review will be automatically added to Publons, you can then choose whether or not to claim your Publons credit. I understand this statement.

Yes Choose an item.
